# Towards the targeted protein degradation of CK2: design and synthesis of CAM4066-based PROTACs

**DOI:** 10.3762/bjoc.22.47

**Published:** 2026-04-22

**Authors:** Sophie Day-Riley, Sona Krajcovicova, Aryaman Raj Sokhal, Jan L Venne, Paul Brear, Marko Hyvönen, Benjamin C Whitehurst, Jason S Carroll, David R Spring

**Affiliations:** 1 Yusuf Hamied Department of Chemistry, University of Cambridge, Lensfield Road, CB2 1EW, Cambridge, United Kingdom; 2 Department of Organic Chemistry, Faculty of Science, Palacky University, Tr. 17. Listopadu 12, Olomouc, 77900, Czech Republic; 3 Department of Biochemistry, University of Cambridge, Tennis Court Road, Cambridge, CB2 1GA, United Kingdom; 4 Hit Discovery, Discovery Sciences, R&D, AstraZeneca, Cambridge, CB2 0AA, United Kingdom; 5 Cancer Research UK Cambridge Institute, Robinson Way, Cambridge, CB2 0RE, United Kingdom

**Keywords:** CAM4066, casein kinase 2 (CK2), PROTACs, targeted protein degradation

## Abstract

Human protein kinase CK2 is a constitutively active serine/threonine kinase implicated in numerous cancers. Although ATP-competitive inhibitors such as CX-4945 show therapeutic potential, they are limited by off-target effects and incomplete or transient CK2 suppression. PROTACs offer an alternative strategy by inducing proteasome-mediated degradation, with potential advantages in potency, selectivity, and duration of action. Herein, a series of CK2-targeting PROTACs has been designed and synthesised. By conjugating a CAM4066-derived warhead to CRBN or VHL ligands, four VHL-recruiting PROTACs, were prepared using PEG and alkyl linkers, alongside two CRBN-recruiting analogues featuring constrained linkers. A ligand–linker analogue in which a linker is projected from the solvent-exposed region of CK2α retained binding affinity comparable to CAM4066, confirming that linker installation is tolerated and preserves key interactions in the αD and ATP sites.

## Introduction

Protein kinases form a large family of more than 500 enzymes that regulate key cellular processes through the phosphorylation of protein substrates. Dysregulation of kinase activity, often through mutation or altered expression, is associated with numerous malignancies [1]. CK2 (previously called casein kinase 2), a constitutively active serine/threonine kinase, functions as a heterotetramer composed of two catalytic (α or α′) and a dimeric regulatory (β) subunit [2]. Unlike most kinases, CK2 does not require upstream activation [3], a property that contributes to its pleiotropic role in cell signalling. Elevated CK2 expression correlates with enhanced cell survival across several cancer types, and its downregulation impairs viability in models of glioblastoma, medulloblastoma, cholangiocarcinoma, breast and renal cancers [4].

These features have established CK2 as a compelling therapeutic target. In 2016, structural studies revealed a cryptic αD pocket adjacent to the ATP-binding site of CK2α [5–6]. Fragment-based ligand discovery subsequently enabled the development of CAM4066, a selective inhibitor that simultaneously engages the αD and ATP sites. CAM4066 validated the αD region as a tractable and selective binding pocket for CK2 kinase inhibition. Despite successes with ATP-competitive inhibitors, small-molecule kinase inhibitors can be limited by reversible target engagement, off-target effects, and susceptibility to resistance mechanisms [7]. These challenges have motivated the exploration of alternative therapeutic modalities that move beyond occupancy-driven pharmacology.

Proteolysis-targeting chimeras (PROTACs) represent a rapidly advancing strategy for induced protein degradation. By transiently engaging both a target protein and an E3 ubiquitin ligase, PROTACs promote proteasomal elimination of the target rather than sustained binding [8]. This event-driven mode of action enables catalytic turnover, can reduce off-target toxicities associated with high inhibitor doses, and does not require a deep or well-defined binding pocket, allowing potential access to targets considered “undruggable” by conventional small molecules [8]. In 2018, a PROTAC targeting CK2 was reported in the literature. This used CX-4945, a potent CK2 inhibitor, as the CK2 warhead [9–10]. However, as CX-4945 targeted the ATP-binding site of CK2, it also displayed nanomolar affinity for the ATP-binding sites of other kinases, like CLK2 [11]. Therefore, finding a potent and selective PROTAC targeting CK2 might be valuable in the evaluation of specific degradation of CK2 as a therapeutic approach.

Herein, we report the design and synthesis of a series of CK2-targeting PROTACs. CAM4066 was selected as the warhead to enable CK2-specific target engagement. We conjugated CAM4066-derived ligands to both CRBN or VHL E3-ligase recruiters. We created VHL-recruiting PROTACs for CK2 using PEG and alkyl linkers, while the CRBN-recruiting analogues featured more constrained linkers. A ligand–linker analogue bearing a linker projected from the solvent-exposed region of the CK2 ligand retained binding affinity comparable to CAM4066, demonstrating that linker installation is well tolerated and preserves key interactions in both the αD and ATP sites.

## Results and Discussion

CAM4066 (**1**) was selected as the CK2-targeting ligand for PROTAC development owing to its high selectivity and well-characterised bivalent binding mode across the ATP and αD pockets. Because a successful degrader must first engage the protein of interest with sufficient affinity, a PROTAC derived from CAM4066 was expected to retain CK2 specificity and achieve kinase-directed degradation rather than broader kinome perturbation. The first design objective was therefore to identify a suitable exit vector for linker attachment that would allow productive E3-ligase recruitment without compromising the established binding geometry of CAM4066.

**Figure 1:**
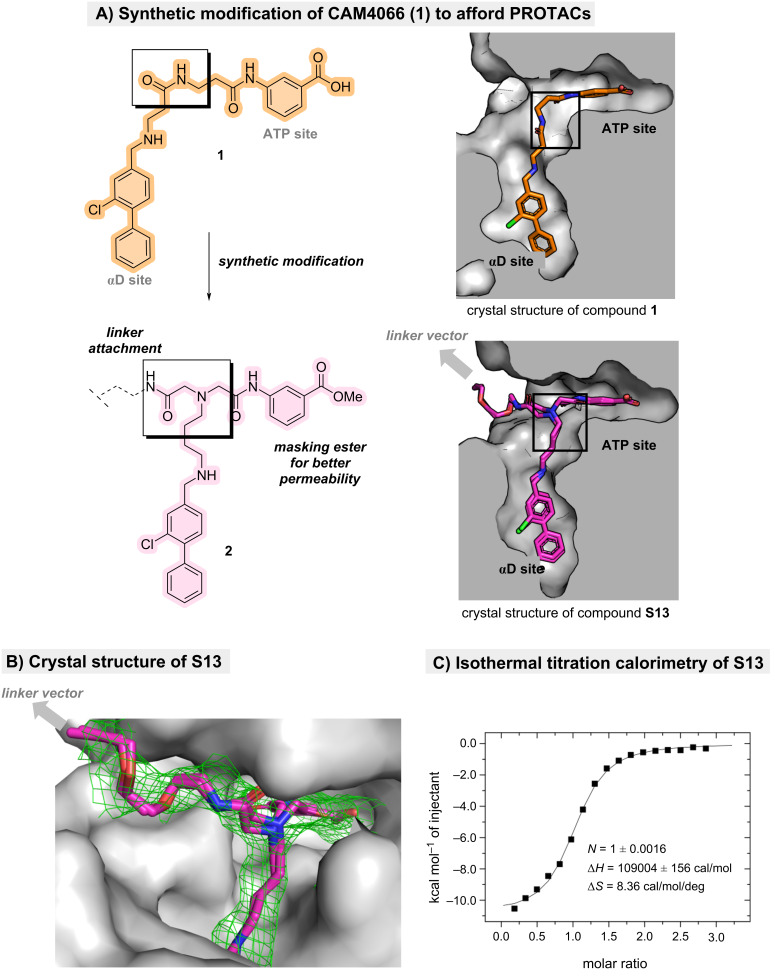
Design strategy and validation. A) Structure of CAM4066 (**1**) that served as a model design for the development of CK2 targeted PROTACs with a general structure **2**. Crystal structures showcased on **1** (PDB: 5CU4) and **S13** (PDB: 9TTA; please see Supporting Information File 1, section 1.4.18 for the full structure). B) Crystal structure of **S13**. The map is Fo-Fc contoured at 1.5 σ. C) Isothermal titration calorimetry (ITC) reveals the suitable position for linker vector to attach the E3 ligases (showcased on compound **S13**).

Inspection of the co-crystal structure of **1** bound to CK2α revealed a small solvent-accessible channel adjacent to the amide linking the ATP- and αD-binding fragments (Figure 1). This channel represented a promising region from which to project a linker, as it was oriented toward solvent and spatially removed from residues critical for **1** binding to CK2α. The amide closest to this opening was therefore identified as the optimal site for structural modification. To allow more flexible functionalisation, the linking region was redesigned to introduce a secondary amine adjacent to the original amide position. This amine maintained the length and orientation of the native framework while serving as a more accessible synthetic handle for downstream coupling chemistry. As **1** itself was known not to have any effect on cell viability while its uncharged methyl ester analogue did, the neutralised pro-drug analogue of the ATP-site binder was employed to prevent such problems [6]. The ATP-site binder and αD-site binder fragments were prepared using literature procedures from the original development of **1**. The modified linking portion containing the new secondary amine was incorporated into the general assembly **2** to afford a CAM4066-derived scaffold suitable for PROTAC construction.

**Scheme 1:**
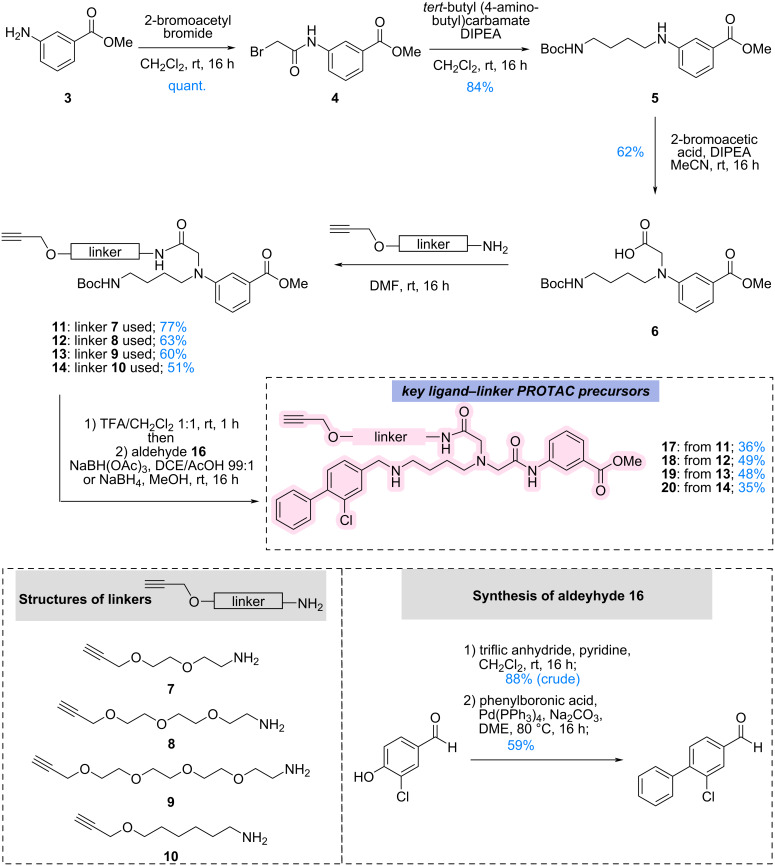
Synthesis of the key ligand-linker PROTAC precursors. For compound **14**, HATU/DIPEA has been used instead of HOBt/DIC. Abbreviations: DCE = 1,2-dichloroethane; DIC = *N*,*N*′-diisopropylcarbodiimide; DIPEA = *N*,*N*-diisopropylethylamine; DME = 1,2-dimethoxyethane; DMF = dimethylformamide; HOBt = 1-hydroxybenzotriazole; TFA = trifluoroacetic acid.

The synthesis of the ATP-site binder began with the alkylation of commercially available methyl 3-aminobenzoate (**3**) using bromoacetyl bromide. Although initial purification via silica gel chromatography led to substantial product loss, isolation by simple aqueous workup afforded the intermediate **4** cleanly and in quantitative yield. This intermediate enabled straightforward elaboration of the ATP-binding fragment, as *N*-Boc-1,4-diaminobutane was subsequently alkylated with intermediate **4** to generate the chain-extended linker precursor **5**. The use of DIPEA instead of triethylamine suppressed undesired alkylation of the base and significantly improved the isolated yield up to 84%. Introduction of an acetic acid handle was then achieved via alkylation with bromoacetic acid under DIPEA mediation, giving intermediate **6**, which served as the common attachment point for linkers **7**–**10** (for full synthesis see Supporting Information File 1, section 1.3). In parallel, PEG-based linkers bearing terminal alkyne handles were synthesised for modular CuAAC-based PROTAC assembly. The αD binder **16** had been synthesised via a two-step reaction. First, the phenolic group of **15** was converted into the corresponding triflate using triflic anhydride, followed by Suzuki–Miyaura cross-coupling with phenylboronic acid to give **16** in 59% yield (Scheme 1).

Boc removal from intermediates **11**–**14** enabled attachment of the αD binder aldehyde **16** via reductive amination. Interestingly, NaBH(OAc)_3_ had performed well in the synthesis of the ligand–linker analogue **17** using intermediate **11**, however, use of intermediates **12**–**14** were consistently giving low yields. Therefore, for those, methanolic reductive amination with NaBH_4_ was pursued. Successful optimisation of reductive amination conditions yielded the key ligand–linker PROTAC precursors **17**–**20** in moderate to good yields (35–49%; Scheme 1).

Before progressing to full PROTACs, it was essential to confirm that the modified CAM4066 scaffold tolerated linker installation. For this, the methyl ester of the key ligand–linker precursor **17** was hydrolysed using aqueous LiOH to yield acid **S13**. Isothermal titration calorimetry (ITC) revealed that **S13** bound CK2α with a dissociation constant (*K*_d_ = 600 nM) comparable to that of CAM4066 (350 nM; Figure 1C).

This indicated that the exit vector modification and linker installation were well tolerated. Co-crystallisation of **S13** with CK2α further confirmed, that both the ATP-binding and αD-binding motifs retained their expected hydrogen-bonding and hydrophobic interactions, and the linker projected cleanly toward solvent-exposed region (Figure 1). Importantly, the absence of the terminal alkyne substituent in the electron density map revealed that it remained solvent-exposed and sterically accessible, validating the design for subsequent attachment of E3-ligase ligands.

**Scheme 2:**
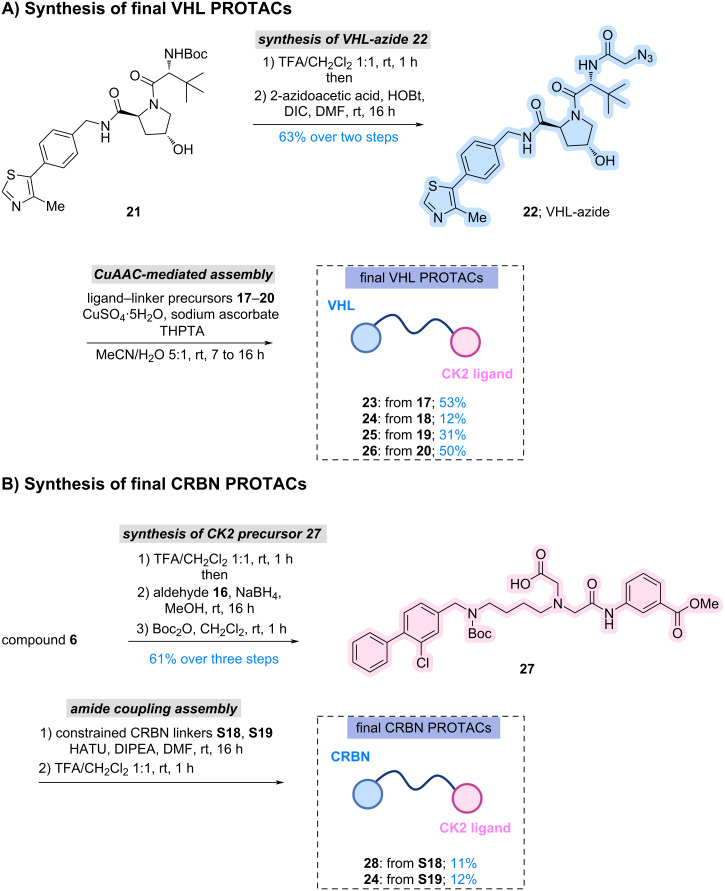
(A) Synthesis of final VHL PROTACs; (B) Synthesis of final CRBN PROTACs. Abbreviations: Boc = *tert*-butoxycarbonyl; CRBN = cereblon; DIPEA = *N*,*N*-diisopropylethylamine; DMF = dimethylformamide; HATU = *O*-(7-azabenzotriazol-1-yl)-*N*,*N*,*N*′,*N*′-tetramethyluronium hexafluorophosphate; THPTA = tris(3-hydroxypropyltriazolylmethyl)amine; TFA = trifluoroacetic acid; VHL = von Hippel–Lindau.

**Figure 2:**
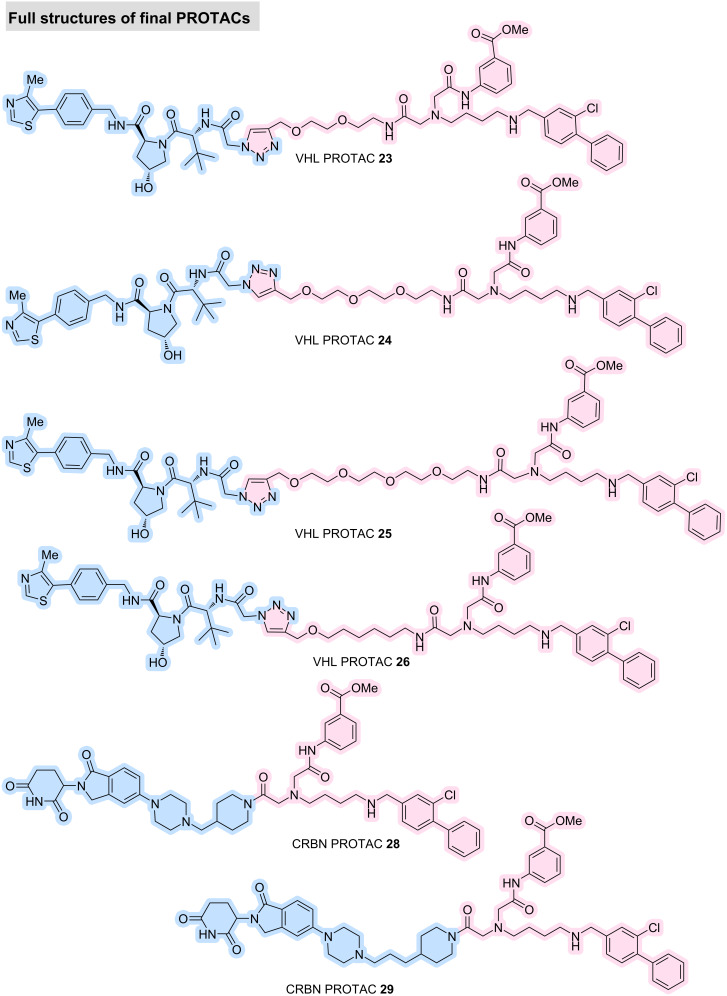
Full structures of final VHL PROTACs **23**–**26** and CRBN PROTACs **28** and **29**.

With the exit vector validated, the synthesis of full PROTACs was pursued. The VH032-derived VHL ligand **21** (for full synthesis see Supporting Information File 1, section 1.4) was prepared according to literature and functionalised with 2-azidoacetic acid to introduce an azido-handle suitable for CuAAC-mediated triazole formation. CuAAC coupling of these alkyne precursors **17**–**20** with the azido-VHL ligand **22** afforded four final VHL-based PROTACs **23**–**26** in moderate to good yields (Scheme 2A, Figure 2). It is important to mention that LCMS analysis indicated complete consumption of the alkyne-bearing intermediates and clean formation of the triazole-linked products after 16 hours, although isolated yields varied due to product loss during preparative RP-HPLC. Two CRBN-based degraders **28** and **29** were synthesised using constrained linker–ligand constructs **S18** and **S19**. Because the steric environment imposed by these linkers differed from that of the VHL system, a modified synthetic sequence was adopted. The αD-site binder **16** was first introduced onto the intermediate **6**, which upon Boc protection yielded intermediate **27**. Installation of CRBN linker–ligands proceeded smoothly under standard coupling conditions using HATU/DIPEA, and Boc removal afforded CRBN PROTACs **28** and **29** (Scheme 2B, Figure 2). Their low isolated yields were ultimately traced to poor solubility in MeCN/H_2_O systems used for preparative purification; the compounds dissolved only in DMSO, explaining the diminished recovery. Nevertheless, the synthetic route successfully delivered two structurally distinct CRBN-recruiting CK2 PROTACs.

All synthesised PROTACs **23**–**26**, **28**, and **29** were evaluated for their ability to induce degradation of CK2α and CK2α′ in cell-based assays. Western blot profiling across six colorectal cancer cell lines revealed heterogeneous expression of both CK2 isoforms. HCT116 cells exhibited robust expression of CK2α and CK2α′, with CK2α predominating, and were therefore selected as the primary model for degradation studies. The MDA-MB-231 breast cancer line was also included due to its prior use in published evaluations of a CK2-targeting PROTAC [10]. The four VHL-based PROTACs **23**–**26** were tested at concentrations up to 10 μM. However, no measurable degradation of CK2α or CK2α′ under these conditions was observed, as indicated by unchanged band intensities relative to controls. At the highest concentration (10 μM), PROTAC **24** caused a loss of both CK2α and vinculin bands, suggesting cytotoxicity rather than target-specific degradation. The two CRBN-based PROTACs **28** and **29** were tested under identical conditions but likewise failed to induce degradation of CK2α or CK2α′ in HCT116 or MDA-MB-231 cells (see Supporting Information File 1, section 2.4). Upon biological evaluation, ITC analysis of **24**, selected as a representative PROTAC, revealed no detectable productive binding event (see Supporting Information File 1, section 2.1). This may have resulted from several factors, including the limited solubility of **24** under the conditions required for the assay and the requirement for cooperative ternary complex formation with the E3 ligase, as the isolated warhead–linker construct **S13** displayed measurable binary binding in the absence of the degrader architecture. Although the ligand–linker analogue showed encouraging biophysical and structural properties, the six full PROTACs did not induce detectable CK2 degradation under the conditions tested. Several mechanistic factors may underlie this outcome. PROTACs frequently fail because the protein of interest and E3 ligase cannot adopt a productive orientation for ubiquitin transfer. Steric constraints, linker vector misalignment, or insufficient linker flexibility may prevent formation of a catalytically competent ternary complex. The azide–alkyne-derived triazole linkage may also impose a rigid orientation not compatible with CK2’s topology. Moreover, PROTACs typically display reduced cellular permeability owing to their high molecular weight and polar surface area [12]. This is particularly relevant given that CAM4066 itself is a moderately polar molecule, and linker addition further increases polarity. Collectively, these proof-of-concept results highlight the complexity of designing bivalent molecules for CK2 degradation and underscore the need for further optimisation of warhead potency, linker length, exit vector geometry, and E3 ligase selection.

## Conclusion

In summary, we have developed a modular synthetic platform for the construction of CK2-targeting degraders **23**–**26**, **28**, and **29** based on the selective bivalent inhibitor CAM4066. Structure-guided design enabled identification of a solvent-accessible exit vector suitable for linker installation, and introduction of a secondary amine at this position provided a versatile handle for downstream functionalisation. The resulting CAM4066-derived ligand–linker analogue **S13** retained high-affinity binding to CK2α and preserved the characteristic bivalent binding mode in co-crystal structures, confirming that the exit vector and linker trajectory were well tolerated. These data established a robust foundation for the subsequent development of PROTACs.

Using this scaffold, we successfully synthesised a set of PROTAC precursors and completed six full degraders recruiting either VHL or CRBN. Although none of the prepared PROTACs induced degradation of CK2α or CK2α′ in cellular assays, the work provides valuable insights into the challenges associated with degrading compact kinases such as CK2. The modest potency of the modified CAM4066 warheads, potential geometric incompatibility within the ternary complex, and the inherent permeability limitations of high-molecular-weight degraders likely contributed to the lack of observed activity.

Despite these limitations, the study offers important lessons for future design. The validated exit vector and structural understanding of warhead tolerance provide a strong basis for further optimisation, including exploration of alternative warheads with improved potency, such as recently developed APL-5125 [13], a larger set of varied linker architectures [14–15], and engagement of additional E3 ligases [16]. More broadly, the synthetic strategy and the determination of tolerance of exit vector position developed here will support continued efforts to achieve targeted degradation of CK2.

Taken together, this work establishes the key design principles, synthetic routes, and structural requirements necessary for next-generation CK2 degraders and lays the groundwork for future optimisation toward achieving effective and selective CK2 degradation.

## Supporting Information

File 1 Experimental part and copies of NMR spectra.

## Data Availability

All data that supports the findings of this study is available in the published article and/or the supporting information of this article.
