# Supplementary material for: Towards the targeted protein degradation of CK2: design and synthesis of CAM4066-based PROTACs
Source: Beilstein J Org Chem. 2026 Apr 22;22:611–9. doi: 10.3762/bjoc.22.47 (PMC13159317; doi:10.3762/bjoc.22.47)
Supplement: File 1 — Experimental part and copies of NMR spectra. [file Beilstein_J_Org_Chem-22-611-s001.pdf]

BEILSTEIN JOURNAL OF ORGANIC CHEMISTRY

Supporting Information

for

Towards the targeted protein degradation of CK2: design and synthesis of CAM4066-based PROTACs

Sophie Day-Riley, Sona Krajcovicova, Aryaman Raj Sokhal, Jan L. Venne, Paul Brear, Marko Hyvönen, Benjamin C. Whitehurst, Jason S. Carroll and David R. Spring

Beilstein J. Org. Chem. **2026,** *22,* 611–619. doi:10.3762/bjoc.22.47

Experimental part and copies of NMR spectra

License and Terms: This is a supporting information file under the terms of the Creative Commons Attribution License (https://creativecommons.org/licenses/by/4.0). Please note that the reuse, redistribution and reproduction in particular requires that the author(s) and source are credited and that individual graphics may be subject to special legal provisions.
The license is subject to the *Beilstein Journal of Organic Chemistry* terms and conditions: (https://www.beilstein-journals.org/bjoc/terms)

Table of contents

Table of contents S1
1. Experimental procedures S2
1.1 General information S2
1.2 Instrumentation S2
1.3 General procedures S4
1.3.1 General procedure A: *tert*-butyloxycarbonyl (Boc) protection S4
1.3.2 General procedure B: Boc-deprotection followed by HOBt/DIC amide coupling S4
1.3.3 General procedure C: reductive amination S5
1.3.4 General procedure D: one pot CuAAC “click” reaction, followed by Boc deprotection to the PROTACs S5
1.4 Experimental S5
1.4.1 Synthesis of compound **4** S5
1.4.2 Synthesis of compound **5** S6
1.4.3 Synthesis of compound **6** S7
1.4.4 Synthesis of compound **S2** S7
1.4.5 Synthesis of compound **S3** (Boc-protected linker **7**) S8
1.4.6 Synthesis of compound **11** S9
1.4.7 Synthesis of compound **S5** S9
1.4.8 Synthesis of compound **S6** (Boc-protected linker **8**) S10
1.4.9 Synthesis of compound **12** S10
1.4.10 Synthesis of compound **S8** S11
1.4.11 Synthesis of compound **S9** (Boc-protected linker **9**) S12
1.4.12 Synthesis of compound **13** S12
1.4.13 Synthesis of compound **S11** (Boc-protected linker **10**) S13
1.4.14 Synthesis of compound **14** S14
1.4.15 Synthesis of compound **S12** S15
1.4.16 Synthesis of αD binder aldehyde **16** S15
1.4.17 Synthesis of ligand–linker precursor **17** S16
1.4.18 Synthesis of compound **S13** S17
1.4.19 Synthesis of ligand–linker precursor **18** S18
1.4.20 Synthesis of ligand–linker precursor **19** S19
1.4.21 Synthesis of ligand–linker precursor **20** S20
1.4.22 Synthesis of compound **S15** S21
1.4.23 Synthesis of compound **S16** S21
1.4.24 Synthesis of compound **S17** S22
1.4.25 Synthesis of VHL Boc **21** S23
1.4.26 Synthesis of VHL azide **22** S23
1.4.27 Synthesis of final VHL PROTAC **23** S24
1.4.28 Synthesis of final VHL PROTAC **24** S25
1.4.29 Synthesis of final VHL PROTAC **25** S26
1.4.30 Synthesis of final VHL PROTAC **26** S26
1.4.31 Synthesis of CK2 precursor **27** S27
1.4.32 Synthesis of final CRBN PROTAC **28** S28
1.4.33 Synthesis of final CRBN PROTAC **29** S29
2. Biological and biophysical evaluation S31
2.1 Isothermal titration calorimetry (ITC) S31
2.2 Cell lines S31
2.3 Cell culture S31
2.4 Treatment with PROTACs S32
2.5 SDS-PAGE followed by Western blot S32
2.5.1 Lysate preparation S32
2.5.2 SDS-PAGE S32
2.5.3 Western blot preparation S32
2.5.4 Western blot results S33
2.5.5 Uncropped Western blots S34
2.6 Sources of antibodies and reagents used S37
3. References S38
4. Copies of NMR spectra S39

1. Experimental procedures

1.1 General information

Room temperature (rt) refers to ambient temperature. Temperatures of −10 °C were maintained using an acetone–ice bath. All temperatures below −10 °C were maintained using an acetone–cardice bath. Temperatures of 0 °C were maintained using an ice–water bath. Reactions were carried out under a stream of nitrogen using oven-dried glassware unless otherwise stated. Heating was provided by conventional stirrer hotplates unless otherwise stated. Anhydrous solvents were stored as received from commercial suppliers. All other solvents and reagents were obtained from commercial suppliers and used without further purification from Sigma-Aldrich (US), Acros Organics (BE), or Fluorochem (UK). Software used: chemical structures (Chemdraw), NMR (MestReNova and TopSpin), Figures (created with BioRender or MS Powerpoint), citations (Zotero).

1.2 Instrumentation

UV–LCMS analysis was carried out using a Waters ACQUITY H-Class UPLC with an ESCi Multi-Mode Ionisation Waters SQ Detector 2 spectrometer using ACQUITY UPLC® CSH C18 (2.1 mm × 50 mm, 1.7 μM, 130 Å) column at 40 °C and flow rate 0.6 mL/min. Mobile phase was (A) 2 mM ammonium acetate in water/MeCN 95:5, (B) MeCN and (C) 2% aq. formic acid, linearly programmed from 5–95% B over 5 min with constant 5% C over 1 min. The ESI source operated at a discharge current of 5 μA, vaporiser temperature of 350 °C and capillary temperature of 200 °C. Chromatographs were monitored by UV absorbance using a photodiode array detector at a wavelength range of 210–800 nm, interval 1.2 nm.

Preparative HPLC was carried out on an Agilent 1260 Infinity using a Supelcosil ABZ+PLUS column (250 mm × 21.2 mm, 5 μM) eluting with a linear gradient system with mobile phases (A) 0.1% TFA in water (v/v) and (B) 0.05% TFA in MeCN (v/v) over 20 min at a flow rate of 20 mL/min.

Mass-directed automatic purification (MDAP) HPLC was carried out on a Waters AutoPurification mass-directed HPLC system with photodiode array detector, SQ Detector 2 and at-column dilution. The analytical runs used an X-Select CSH C18 column (4.6 × 150mm, 5 μm particle size) and flow rate of 1.2 mL/min. The preparative runs used an X-Select CSH Prep C18 OBD column (19 × 150mm, 5 μM particle size) and flow rate of 20 mL/min. The machine used a linear gradient of solvent B in solvent A run over 15 min with 1 min isocratic hold before the gradient (analytical) or over 15 min with 1.4 min isocratic hold (preparative). For acidic runs, solvent A was water with 0.1% (v/v) formic acid and solvent

B was MeCN with 0.1% (v/v) formic acid. For neutral runs solvent A was water (no additives) and solvent B was MeCN (no additives).

Proton nuclear magnetic resonance (^{1}H NMR) spectra were recorded at ambient temperature (unless otherwise stated) on a Bruker DPX-400 (400 MHz), Bruker Avance 400 QNP (400 MHz), Bruker Avance 500 Cryo Ultrashield (500 MHz) or Bruker TXO Cryo Probe (700 MHz). ^{1}H NMR chemical shifts are reported in parts per million (ppm), to the nearest 0.01 ppm and are referenced to the residual non-deuterated solvent peak ($CDCl_3$: 7.26, MeOD: 3.31, DMSO-d_6: 2.50 ppm). Coupling constants (J) are reported in hertz (Hz) to the nearest 0.1 Hz. Data are reported as follows: chemical shift, multiplicity (app = apparent; s = singlet; d = doublet; t = triplet; q = quartet; qn = quintet; m = multiplet; or as a combination of these), coupling constant(s), integration, and assignment. Carbon NMR (^{13}C NMR) spectra were recorded at ambient temperature on a Bruker DPX-400 (101 MHz), Bruker Avance 400 QNP (101 MHz), Bruker Avance 500 Cryo Ultrashield (126 MHz) or Bruker TXO Cryo Probe (176 MHz). Chemical shifts are quoted in ppm, to the nearest 0.1 ppm, and are referenced to the residual non-deuterated solvent peak ($CDCl_3$: 77.2, MeOD: 49.0, DMSO-d_6: 39.50 ppm). ^{1}H NMR and ^{13}C NMR spectra assignments were supported by DEPT–135, COSY, ^{1}H−^{13}C HSQC, ^{1}H−^{13}C HMBC, and NOESY where appropriate.

Liquid chromatography mass spectrometry (LCMS) was carried out using a Waters ACQUITY HClass UPLC with an ESCi Multi-Mode Ionisation Waters SQ Detector 2 spectrometer using MassLynx 4.1 software; ESI refers to the electrospray ionisation technique; LC system: solvent A: 2 mM NH_4OAc in H_2O/MeCN 95:5; solvent B: MeCN; solvent C: 2% formic acid (aq); column: ACQUITY UPLC CSH C18 (2.1 mm × 50 mm, 1.7 µm, 130 Å) at 40 °C; gradient: 5–95% B with constant 5% C, over 1 minute at a flow rate of 0.6 mL/minute; detector: PDA eλ Detector 220–800 nm, interval 1.2 nm.

Infrared spectroscopy (IR) was carried out using an Agilent Cary 630 FTIR. Major peaks are reported.

High-resolution mass spectrometry (HRMS) measurements were recorded with an Agilent 6230 LC/TOF HRMS system. Mass values are reported within the 5 ppm error limit.

Thin layer chromatography (TLC) was performed using pre-coated Merck glass backed silica gel 60 F254 plates and visualised by UV fluorescence (λ_{max} = 254 nm) or by staining with potassium permanganate. Retention factors (R_f) are quoted to 0.01.

Flash column chromatography was carried out using Merck 9385 Kieselgel 60 SiO_2 (230–400 mesh) under a positive pressure of air unless otherwise stated. Automated flash column chromatography was carried out on a Combiflash Rf200 automated chromatography system with Redisep® reverse-phase

C18-silica flash columns (20–40 μm). Analytical high-performance liquid chromatography (HPLC) was carried out using an Agilent 1260 Infinity system with a reversed-phase Supelcosil™ ABZ+PLUS column (150 mm × 4.6 mm, 3 μm) eluting with a gradient system (solvent A: 0.05% (v/v) TFA in H_2O, solvent B: 0.05% (v/v) TFA in MeCN; 5% to 95% A to B) over 15 minutes, unless otherwise stated, at a flow rate of 1 mL/min. HPLC was monitored by UV absorbance at 220 and 254 nm. Mass-directed HPLC was carried out on a Waters Mass Directed Autopurification System using an SQD2 detector and XSelect® CSH (C18, 5 μM, 19 × 150 mm) column.

1.3 General procedures

1.3.1 General procedure A: tert-butyloxycarbonyl (Boc) protection

To a stirring solution of the corresponding amine (1 equiv) in CH_2Cl_2 at room temperature was added a solution of di-*tert*-butyl-dicarbonate (1.2 equiv) in CH_2Cl_2. The reaction mixture was stirred for 16 hours at room temperature. Upon completion (monitored by TLC CH_2Cl_2/MeOH 10:1), the reaction mixture was concentrated in vacuo and the crude product was purified via silica gel flash column chromatography (5% MeOH in CH_2Cl_2) to yield the desired product.

1.3.2 General procedure B: Boc-deprotection followed by HOBt/DIC amide coupling

Boc-protected amine linker (1.2 equiv) was dissolved in a mixture of TFA/CH_2Cl_2 1:1 (v/v) at 0 °C. The reaction mixture was stirred for 1 hour after which LC–MS confirmed removal of the Boc protecting group. The residual solvents were evaporated under a stream of nitrogen, the crude amine linker-TFA salt was redissolved in DMF (2.5 mL), and DIPEA (4.0 equiv) was added. To a solution of **6** (1.0 equiv) in DMF (2.5 mL) HOBt (1.3 equiv) was added at room temperature and the mixture was stirred for 10 min before DIC (1.3 equiv) was added. The reaction mixture was stirred for a further 10 min before the solution of the amine was added dropwise. The reaction mixture was left to stir for 16 hours at room temperature. The mixture was quenched with 10% aqueous citric acid solution (5 mL) and extracted with EtOAc (3 × 5 mL). Organic extracts were combined, dried over $MgSO_4$, filtered, and concentrated in vacuo. The crude product was purified by reversed phase column chromatography (C_{18} silica gel, 10–100% solvent B in solvent A. Solvent A: 5% formic acid solution (aq). Solvent B: MeCN) to yield the desired product.

1.3.3 General procedure C: reductive amination

Boc-protected amine (1 equiv) was dissolved in a mixture of TFA/CH_2Cl_2 1:1 (v/v) at 0 °C. The reaction mixture was stirred for 1 hour at room temperature, after which LC–MS confirmed removal of the Boc protecting group. The residual solvents were evaporated under stream of nitrogen, the residue was redissolved in methanol (5 mL) and the pH was adjusted to 7 using DIPEA. Then, aldehyde **16** (1.2 equiv) was added, and the reaction mixture was stirred at room temperature for 16 hours. After that, $NaBH_4$ (1.2 equiv) was added, and the reaction mixture was stirred for another hour after which LC–MS confirmed formation of desired product. Following evaporation of residual solvents and purification by reversed phase column chromatography (C_{18} silica gel, 10-100% solvent B in solvent A. Solvent A: 0.1 M ammonia solution (aq). Solvent B: MeCN), the desired product was obtained.

1.3.4 General procedure D: one pot CuAAC "click" reaction, followed by Boc deprotection to the PROTACs

A stirring solution of the respective ligand–linker PROTAC precursors (1.0 equiv) and VHL-azide **22** (1.0 equiv) in MeCN/H_2O 5:1 (v/v, 1 mL) was degassed with a stream of N_2 for 15 min. Tris(3-hydroxypropyltriazolylmethyl)amine (THPTA; 2.0 equiv) and sodium ascorbate (6.0 equiv) were added, followed by $Cu_2SO_4{\cdot}5H_2O$ (2.0 equiv) and the reaction was stirred at room temperature for 7–16 hours, after which LC–MS confirmed formation of desired product. The reaction was concentrated in vacuo. The residue was dissolved in TFA/CH_2Cl_2 1:1 (v:v) and stirred at room temperature for 2 hours after which LC–MS confirmed formation of the Boc-deprotected products. The reaction was concentrated in vacuo and purified via the reversed phase HPLC to yield the desired VHL-PROTAC.

1.4 Experimental

1.4.1 Synthesis of compound 4

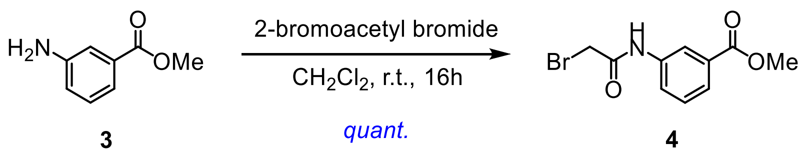

3 *quant.* 4

To a stirring solution of methyl 3-aminobenzoate (**3**, 3 g, 20 mmol, 1.0 equiv) in CH_2Cl_2 (15 mL) at 0 °C was added 2-bromoacetyl bromide (1.7 mL, 20 mmol, 1.0 equiv) dropwise. The mixture was allowed to stir for 1 hour at 0 °C then at room temperature for 16 hours. Upon completion (monitored by TLC, 5% MeOH/CH_2Cl_2), the reaction mixture was diluted with CH_2Cl_2 (150 mL) and extracted with aqueous 3 M

NH_4Cl solution (3 × 150 mL). The organic layers were combined, dried over $MgSO_4$, filtered, and concentrated in vacuo to yield the title compound **4** as a brown oil (5.4 g, 20 mmol, quant.) with no need for further purification.

R*f* : 0.71 (5% MeOH/ CH_2Cl_2)

LC-MS (ESI): $[M-H]^-$ calc. for $[C_{10}H_9BrNO_3]^-$: 271.0, found 270.0

^{1}**H NMR** (500 MHz, $CDCl_3$): δ 8.24 (s, 1H), 8.06–8.05 (m, 1H), 7.91–7.89 (m, 1H), 7.84–7.82 (m, 1H), 7.45–7.42 (m, 1H), 4.04 (s, 2H), 3.93 (s, 3H) ppm

^{13}**C NMR** (126 MHz, $CDCl_3$): δ 166.5, 163.7, 137.2, 131.0, 129.3, 126.2, 124.4, 120.9, 52.4, 29.3 ppm

Note: Data were in accordance with those reported previously in literature.[1] *CH_2Cl_2, used as a transferring agent, present in ^{1}H NMR at 5.30 (s) ppm and ^{13}C NMR at 53.46 ppm.*

1.4.2 Synthesis of compound 5

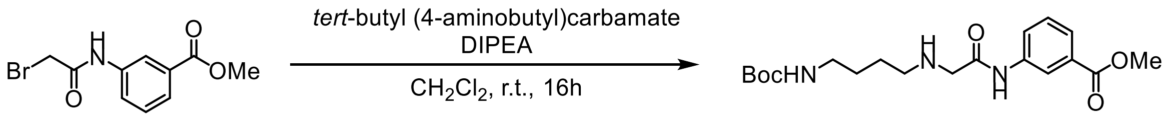

4 **5**

To a stirring solution of **4** (4.2 g, 15 mmol, 1.0 equiv) in CH_2Cl_2 (40 mL) were added *tert*-butyl (4-aminobutyl)carbamate (3.8 mL, 20 mmol, 1.3 equiv) and DIPEA (3.9 mL, 22 mmol, 1.5 equiv). The mixture was stirred at room temperature for 16 hours. Upon completion (monitored by TLC, 5% MeOH in CH_2Cl_2) the reaction mixture was diluted with CH_2Cl_2 (60 mL) and extracted with saturated aqueous $NaHCO_3$ solution (3 × 100 mL), dried over $MgSO_4$, filtered, and concentrated in vacuo. The crude residue was purified by reversed phase column chromatography (C_{18} silica gel, 10- 90% solvent B in solvent A; Solvent A: 0.5% formic acid (aq). Solvent B: MeCN) to afford the title compound **5** as a brown oil (4.8 g, 13 mmol, 84%).

R*f* : 0.28 (5% MeOH in CH_2Cl_2)

HRMS (ESI): $[M+H]^+$ calc. for $C_{19}H_{30}O_5N_3^+$: 380.2180, found 380.2165.

ν_{max} (neat)/cm^{-1}: 3323 (m, N-H) , 2932 (m, C-H), 2110 (w, alkyne), 1683 (s, C=O), 1593 (m, C=C), 1524 (m, C=C), 1440 (s, C-H), 1365 (s, C-H), 1287 (m, N-H), 1247 (m, C-O), 1166 (s, C-O), 757 (s, C-H).

^{1}**H NMR** (500 MHz, $CDCl_3$): δ 8.06–8.05 (m, 1H), 8.00–7.98 (m, 1H), 7.78–7.76 (m, 1H), 7.42–7.38 (m, 1H), 3.91 (s, 3H), 3.39 (s, 2H), 3.16–3.15 (m, 2H), 2.72–2.69 (m, 2H), 1.57–1.55 (m, 4H), 1.43 (s, 9H) ppm.

^{13}C NMR (126 MHz, $CDCl_3$): δ 170.1, 166.7, 156.0, 137.8, 130.9, 129.2, 125.1, 123.9, 120.2, 79.2, 52.9, 52.2, 49.9, 40.3, 27.8, 27.2 ppm.

1.4.3 Synthesis of compound 6

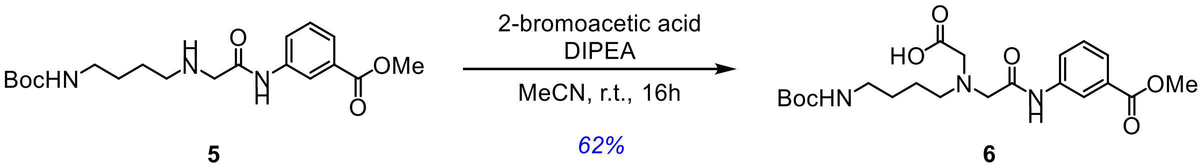

5 **6**

To a stirring solution of **5** (2.4 g, 6.3 mmol, 1.0 equiv) in acetonitrile (80 mL) was added DIPEA (1.4 mL, 8.0 mmol, 1.3 equiv). The mixture was stirred for 30 min at room temperature before 2-bromoacetic acid (1.2 g, 8.6 mmol, 1.4 equiv) was added and the reaction mixture was stirred at room temperature for 16 hours. Upon completion (monitored by TLC, 5% MeOH in CH_2Cl_2), the mixture was concentrated in vacuo and purified by reverse phase column chromatography (C_{18} silica gel, 10–100% solvent B in solvent A. Solvent A: 5% formic acid solution (aq). Solvent B: MeCN over 20 min) to yield the title compound **6** as a white solid (1.70 g, 3.9 mmol, 62% yield).

R_f : 0.20 (5% MeOH in CH_2Cl_2)

HRMS (ESI): $[M+H]^+$ calc. for $C_{21}H_{32}N_3O_7^+$: 438.2235, found 438.2232

ν_{max} (neat)/cm^{-1}: 3386 (br, O-H), 2979 (C-H), 1696 (C=O), 1623 (C=C), 1597 (C=C), 1569 (C=C), 1445 (C-H), 1391 (C-H), 1367 (C-H), 1291 (N-H), 1287 (C-O), 1167 (C-O), 760 (C-H)

^{1}H NMR (500 MHz, $CDCl_3$): δ 8.16 (br s, 1H), 7.90–7.89 (m, 1H), 7.73–7.72 (m, 1H), 7.36–7.33 (m, 1H), 3.90 (s, 3H), 3.39 (s, 2H), 3.67 (s, 2H), 3.11 (s, 2H), 3.09–2.89 (m, 2H), 1.61–1.46 (m, 4H), 1.43 (s, 9H) ppm

^{13}C NMR (126 MHz, $CDCl_3$): δ 172.7, 168.2, 166.8, 156.2, 137.9, 130.7, 129.1, 125.3, 124.2, 120.5, 79.4, 58.6, 56.6, 55.6, 52.2, 39.9, 28.3, 27.4, 23.7 ppm

1.4.4 Synthesis of compound S2

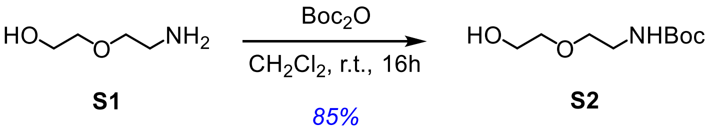

S1 **S2**

Prepared by *General Procedure A* with 2-(2-aminoethoxy)ethan-1-ol (**S1**, 0.50 g, 4.8 mmol, 1.0 equiv), di-*tert*-butyl dicarbonate (1.24 g, 5.7 mmol, 1.2 equiv) and CH_2Cl_2 (12 mL). The crude product was

purified by silica gel flash column chromatography (5% MeOH in CH_2Cl_2) to afford the title compound **S2** as a yellow oil (0.85 g, 4.1 mmol, 85%).

R_f : 0.44 (5% MeOH in CH_2Cl_2).

***v*$_{max}$** (neat)/cm^{-1}: 3675 (br, O-H), 3366 (w, N-H), 2973 (m, C-H), 2901 (m, C-H), 1687 (s, C=O).

^{1}H NMR (700 MHz, $CDCl_3$): δ 3.66 (t, *J* = 4.5, 2H, H), 3.50 (t, *J* = 4.5 Hz, 2H, H), 3.48 (t, *J* = 5.2 Hz, 2H, H), 3.25 (t, *J* = 4.8 Hz, 2H, H), 1.37 (s, 9H, H) ppm.

^{13}C NMR (176 MHz, $CDCl_3$): δ 156.2 (C^3), 79.3 (C^2), 72.2 (C^6), 70.2 (C^5), 61.4 (C^7), 30.3 (C^4), 28.3 (C^1) ppm.

Note: Data are in accordance with those reported previously in literature.[2]

1.4.5 Synthesis of compound S3 (Boc-protected linker 7)

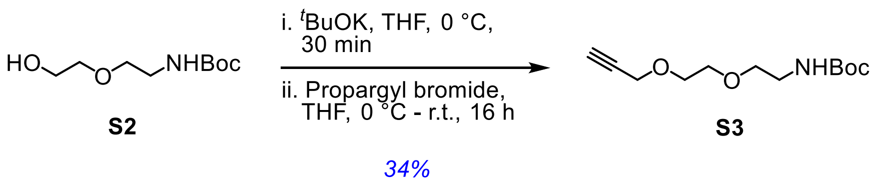

To a stirring solution of *tert*-butyl (2-(2-hydroxyethoxy)ethyl)carbamate (**S2**, 200 mg, 0.97 mmol, 1.0 equiv) in THF (6 mL) at 0 °C was added KO*t*-Bu (168 mg, 1.50 mmol, 1.5 equiv). The solution was allowed to stir for 30 min before propargyl bromide (80% w/v, 104 µL, 0.97 mmol, 1.0 equiv) was added, the solution was warmed to room temperature and stirred for 16 hours. Upon completion (monitored by TLC, 5% MeOH in CH_2Cl_2) the residual base was filtered off and residual solvents were evaporated. The crude mixture was purified by silica gel flash column chromatography (0 to 50% EtOAc in hexane) to afford the title compound **S3** as a yellow oil (80 mg, 0.33 mmol, 34%).

R_f : 0.58 (5% MeOH in CH_2Cl_2)

^{1}H NMR (500 MHz, $CDCl_3$): δ 4.14 (d, *J* = 2.3, 2H), 3.64–3.57 (m, 4H) 3.48 (t, *J* = 5.2 Hz, 2H), 3.28 (t, *J* = 5.2 Hz, 2H), 2.40 (t, *J* = 2.4 Hz, 2H), 1.30 (s, 9H) ppm

^{13}C NMR (126 MHz, $CDCl_3$): δ 159.9, 79.5, 79.1, 74.6, 70.2, 70.0, 68.9, 58.3, 40.3, 28.3 ppm

Note: Data are in accordance with those reported previously in literature.[2]

1.4.6 Synthesis of compound 11

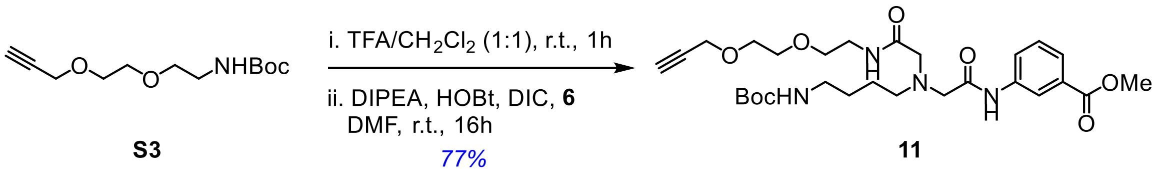

Prepared by *General Procedure B* with **6** (131 mg, 0.30 mmol, 1.0 equiv), **S3** (88 mg, 0.36 mmol, 1.2 equiv), HOBt (60 mg, 0.39 mmol, 1.3 equiv) and DIC (62 µL, 0.40 mmol, 1.3 equiv). The crude reaction mixture was purified via reversed phase column chromatography (C_{18} silica gel, 10–100% solvent B in solvent A. Solvent A: 5% formic acid solution (aq). Solvent B: MeCN) to yield the desired product to yield the title compound **11** as a yellow oil (130 mg, 0.23 mmol, 77%).

R_f : 0.21 (5% MeOH in CH_2Cl_2)

HRMS (ESI): $[M+H]^+$ calc. for $C_{28}H_{43}N_4O_8^+$: 563.3081, found 563.3088

$\boldsymbol{v}_{\mathbf{max}}$ (neat)/cm^{-1}: 3283 (m N-H), 2930 (m, C-H), 2869 (m, C-H), 1714 (s, C=O), 1661 (s, C=C), 1593 (m, C=O), 1530 (m, C=C), 1440 (m, C-H), 1365 (m, C-H), 1287 (s, C-O), 1248 (C-N), 1165 (s, C-H)

^{1}H NMR (500 MHz, $CDCl_3$): δ 8.26–8.25 (m, 1H), 7.96–9.94 (m, 1H), 7.78–7.56 (m, 1H), 7.41–7.38 (m, 1H), 4.15 (d, *J* = 2.4 Hz, 2H), 3.91 (s, 3H), 3.68–3.63 (m, 4H), 3.60–3.58 (m, 2H), 3.54–3.51 (m, 2H), 3.33 (s, 2H), 3.27 (s, 2H), 2.69–2.66 (m, 2H), 2.67 (t, *J* = 6.9 Hz, 2H), 2.44 (t, *J* = 2.4 Hz, 1H), 1.54–1.51 (m, 4H), 1.41 (s, 9H) ppm

^{13}C NMR (126 MHz, $CDCl_3$): δ 170.8, 169.7, 166.8, 156.2 , 138.2, 130.8, 129.0, 125.2, 124.3, 120.7, 79.4, 79.3, 74.8, 69.9, 69.5, 69.0, 59.7, 58.9, 58.4, 55.4, 52.1, 39.9, 39.1, 28.4, 27.5, 24.1 ppm

1.4.7 Synthesis of compound S5

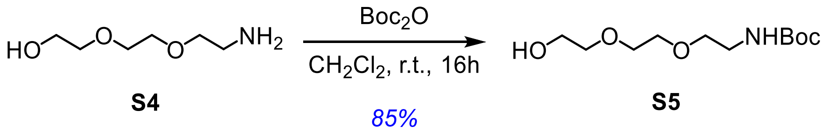

Prepared by *General Procedure A* with 2-(2-(2-aminoethoxy)ethoxy)ethan-1-ol (**S4**, 385 mg, 2.6 mmol, 1.0 equiv), di-*tert*-butyl dicarbonate (675 mg, 3.1 mmol, 1.2 equiv) and CH_2Cl_2 (12 mL). The crude product was purified by silica gel flash column chromatography (5% MeOH in CH_2Cl_2) to afford the title compound **S5** as a yellow oil (560 mg, 2.2 mmol, 8%).

R_f : 0.40 (5% MeOH in CH_2Cl_2)

ν_{max} (neat)/cm^{-1}: 3684 (br O-H), 3374 (w, N-H), 2987 (m, C-H), 2901 (m, C-H), 1240 (s, C-O), 1691 (C=O)

^{1}H NMR (500 MHz, $CDCl_3$): δ 3.73–3.71 (m, 2H), 3.65–3.58 (m, 6H), 3.53 (t, *J* = 5.2 Hz, 2H), 3.29 (t, *J* = 5.1 Hz, 2H), 1.42 (s, 9H) ppm

^{13}C NMR (126 MHz, $CDCl_3$): δ 156.0, 79.3, 72.6, 70.4, 70.3, 61.7, 40.4, 28.4 ppm

Note: Data are in accordance with those reported previously in literature.[3]

1.4.8 Synthesis of compound S6 (Boc-protected linker 8)

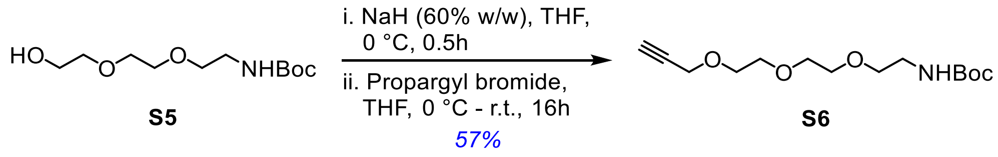

To a stirring solution of *tert*-butyl (2-(2-(2-hydroxyethoxy)ethoxy)ethyl)carbamate (**S5**, 100 mg, 0.40 mmol, 1.0 equiv) in THF (6 mL) at 0 °C was added NaH (60% w/w, 20 mg, 0.50 mmol, 1.3 equiv). The solution was allowed to stir for 30 min before propargyl bromide (80% w/v, 43 µL, 0.40 mmol, 1.0 equiv) was added, the solution was warmed to room temperature and stirred for 16 hours. Upon completion (monitored by TLC CH_2Cl_2/MeOH 10:1), the reaction mixture was diluted with saturated aqueous NH_4Cl solution (3 mL) and extracted with EtOAc (3 × 3 mL). The organic layers were combined, dried over $MgSO_4$, filtered and concentrated in vacuo. The crude mixture was purified by silica gel flash column chromatography (0 to 50% EtOAc in hexane) to afford the title compound **S6** as a yellow oil (66 mg, 0.23 mmol, 57%).

R_f : 0.47 (5% MeOH in CH_2Cl_2)

^{1}H NMR (700 MHz, $CDCl_3$): δ 4.15 (d, *J* = 2.4 2H), 3.70–3.65 (m, 4H), 3.63–3.59 (m, 4H), 3.56 (t, *J* = 5.1 Hz, 2H), 3.48 (t, *J* = 5.2 Hz, 2H), 3.24 (t, *J* = 4.7 Hz, 2H), 2.40 (t, *J* = 2.3 Hz, 1H), 1.38 (s, 9H) ppm

^{13}C NMR (176 MHz, $CDCl_3$): δ 156.0, 79.6, 79.2, 74.5, 70.5, 70.4, 70.2, 69.1, 58.3, 53.4, 40.4, 28.4 ppm

Note: Data are in accordance with those reported previously in literature.[3]

1.4.9 Synthesis of compound 12

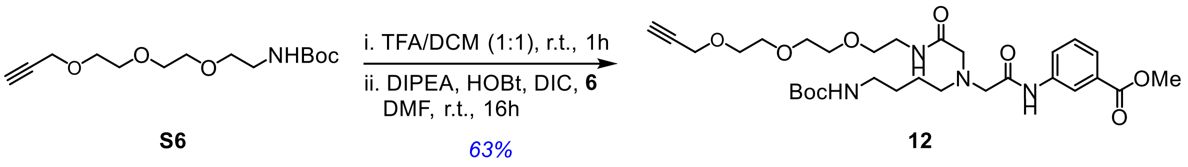

Prepared by *General Procedure B* with **6** (87 mg, 0.20 mmol, 1.0 equiv), **S6** (69 mg, 0.24 mmol, 1.2 equiv), HOBt (40 mg, 0.26 mmol, 1.3 equiv) and DIC (41 μL, 0.26 mmol, 1.3 equiv). The crude product was purified by reversed phase column chromatography (C_{18} silica gel, 10–100% solvent B in solvent A. Solvent A: 5% formic acid solution (aq). Solvent B: MeCN) to yield the title compound **12** as a yellow oil (76 mg, 0.13 mmol, 63%).

R_f : 0.43 (5% MeOH in CH_2Cl_2)

HRMS (ESI): $[M+H]^+$ calc. for $C_{30}H_{47}O_9N_4^+$: 607.3338, found 607.3312

ν_{max} (neat)/cm^{-1}: 3298 (m, N-H), 2931(m, C-H), 2873 (m, C-H), 2090 (w, alkyne), 1712 (s, C=O), 1661 (s, C=C), 1593 (m, C=C), 1531 (s, C=C), 1440 (m, C-H), 1365 (m, C-H), 1287 (s, C-O), 1248 (s, C-N), 1165 (s, C-O), 1150 (s, C-O), 757 (s, C-H)

^{1}H NMR (400 MHz, $CDCl_3$): δ 8.27–8.26 (m, 1H), 7.95–7.93 (m, 1H), 7.70–7.74 (m, 1H), 7.40–7.36 (m, 1H), 4.16 (d, *J* = 2.4 Hz, 2H), 3.90 (s, 3H), 3.68–56 (m, 10H), 3.52–3.49 (m, 2H), 3.33 (s, 2H), 3.28 (s, 2H), 3.11–3.09 (m, 2H), 2.68–2.65 (m, 2H), 2.44 (t, *J* = 2.4 Hz, 1H), 1.53–1.50 (m, 4H), 1.41 (s, 9H) ppm

^{13}C NMR (101 MHz, $CDCl_3$): δ 170.9, 169.8, 166.8, 156.2, 138.3, 130.8, 128.9, 125.1, 124.3, 120.7, 79.3, 79.2, 74.9, 70.4, 70.2, 70.1, 69.5, 69.1, 59.7, 58.8, 58.3, 55.5, 52.1, 40.0, 39.1, 28.4, 27.5, 24.2 ppm

1.4.10 Synthesis of compound S8

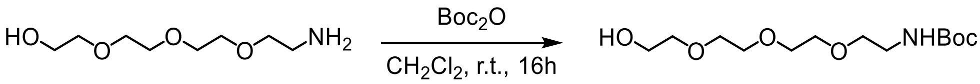

S7 *91%* **S8**

Prepared by *General Procedure A* with 2-(2-(2-(2-aminoethoxy)ethoxy)ethoxy)ethan-1-ol (**S7**, 500 mg, 2.6 mmol, 1.0 equiv), di-*tert*-butyl dicarbonate (675 mg, 3.1 mmol, 1.2 equiv) and CH_2Cl_2 (12 mL). The crude product was purified by silica gel flash column chromatography (5% MeOH in CH_2Cl_2) to afford the title compound **S8** as a yellow oil (695 mg, 2.4 mmol, 91%).

R_f : 0.33 (5% MeOH in CH_2Cl_2)

^{1}H NMR (500 MHz, $CDCl_3$): δ 3.73–3.68 (m, 4H), 3.66–3.59 (m, 8H), 3.52 (t, *J* = 5.1 Hz, 2H), 3.30 (t, *J* = 5.1 Hz, 2H), 1.30 (s, 9H) ppm

^{13}C NMR (126 MHz, $CDCl_3$): δ 156.2, 79.1, 72.6, 70.6, 70.5, 70.4, 70.2, 70.1, 61.6, 40.5, 28.4 ppm

Note: Data are in accordance with those reported previously in literature.[4]

1.4.11 Synthesis of compound S9 (Boc-protected linker 9)

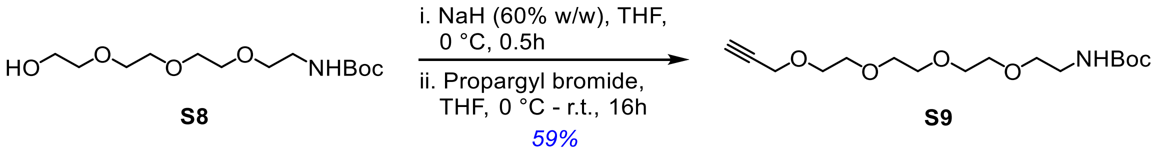

To a stirring solution of *tert*-butyl (2-(2-(2-(2-hydroxyethoxy)ethoxy)ethoxy)ethyl) carbamate (**S8**, 100 mg, 0.34 mmol, 1.0 equiv) in THF (6 mL) at 0 °C was added NaH (60% w/w, 20 mg, 0.51 mmol, 1.5 equiv). The solution was allowed to stir for 30 minutes before propargyl bromide (80% w/v, 36 μL, 0.34 mmol, 1.0 equiv) was added, the solution was warmed to room temperature and stirred for 16 hours. Upon completion (monitored by TLC CH_2Cl_2/MeOH 10:1) the reaction mixture was diluted with saturated aqueous NH_4Cl solution (10 mL) and extracted with EtOAc (3 × 10 mL). The organic layers were combined, dried over $MgSO_4$, filtered and concentrated in vacuo. The crude mixture was purified by silica gel flash column chromatography (0 to 50% EtOAc in hexane) to afford the title compound **S9** as a yellow oil (67 mg, 0.20 mmol, 59%).

R_f : 0.56 (5% MeOH in CH_2Cl_2)

^{1}H NMR (500 MHz, $CDCl_3$): δ 4.20 (d, *J* = 2.4 Hz, 2H), 3.72–3.59 (m, 14H), 3.49 (t, *J* = 5.4 Hz, 2H), 2.42 (t, *J* = 2.4 Hz, 1H), 1.46 (s, 9H) ppm

^{13}C NMR (126 MHz, $CDCl_3$): δ 156.0, 79.6, 79.1, 74.6, 70.5, 70.3, 70.2, 70.1, 69.0, 58.4, 40.3, 28.4 ppm

Note: Data are in accordance with those reported previously in literature.[4]

1.4.12 Synthesis of compound 13

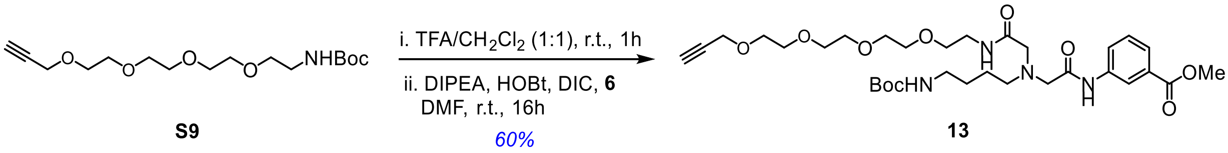

Prepared by *General Procedure B* with **6** (87 mg, 0.20 mmol, 1.0 equiv), **S9** (80 mg, 0.24 mmol, 1.2 equiv), HOBt (40 mg, 0.26 mmol) and DIC (41 μL, 0.26 mmol). The crude product was purified by reversed phase column chromatography (C_{18} silica gel, 10–100% solvent B in solvent A. Solvent A: 5% formic acid solution (aq). Solvent B: MeCN) to yield the title compound **13** as a yellow oil (79 mg, 0.12 mmol, 60%).

R_f : 0.48 (5% MeOH in CH_2Cl_2).

HRMS (ESI): $[M+H]^+$ calc. for $C_{32}H_{51}N_4O_{10}^+$: 561.3600, found 651.3577

ν_{max} (neat)/cm^{-1} : 3290 (m, N-H), 2869 (m, C-H), 2865 (m, C-H), 2115 (w, alkyne), 1712 (s, C=O), 1663 (s, C=C), 1593 (m, C=C), 1531 (s, C=C), 1440 (m, C-H), 1288 (s, C-O), 1248 (m, C-N), 1166 (s, C-O), 1150 (s, C-O), 757 (s, C-H).

^{1}H NMR (400 MHz, $CDCl_3$): δ 8.29–8.28 (m, 1H), 7.96–7.94 (m, 1H), 7.77–7.75 (m, 1H), 7.40–7.37 (m, 1H), 4.17 (d, J = 2.4 Hz, 2H), 3.90 (s, 3H), 3.70–3.56 (m, 14H), 3.53–3.50 (m, 2H), 3.34 (s, 2H), 3.30 (s, 2H), 3.11–3.10 (m, 2H), 2.70–2.67 (m, 2H), 2.44 (t, J = 2.4 Hz, 1H), 1.54–1.52 (m, 4H), 1.42 (s, 9H) ppm

^{13}C NMR (101 MHz, $CDCl_3$): δ 171.1, 170.0, 166.9, 156.2, 138.3, 130.7, 128.9, 125.1, 124.3, 120.7, 79.3, 79.2, 74.8, 70.4, 70.4, 70.3, 70.2, 70.0, 69.6, 69.0, 59.7, 58.8, 58.3, 55.6, 52.1, 40.0, 39.2, 28.4, 28.4, 24.5 ppm

1.4.13 Synthesis of compound S11 (Boc-protected linker 10)

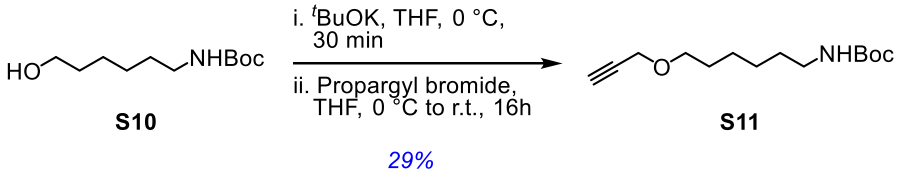

To a stirring solution of *tert*-butyl (6-hydroxyhexyl)carbamate (**S10**, 2.0 g, 9.2 mmol, 1.0 equiv) in THF (60 mL) at 0 °C was added KO*t*-Bu (1.5 g, 13.4 mmol, 1.5 equiv). The solution was allowed to stir for 30 min before propargyl bromide (80% w/v, 1.0 mL, 9.2 mmol, 1.0 equiv) was added, the solution was warmed to room temperature and stirred for 16 hours. Upon completion (monitored by TLC CH_2Cl_2/MeOH 10:1) was the residual base filtered off and residual solvents were evaporated. The crude mixture was purified by silica gel flash column chromatography (0 to 50% EtOAc in hexane) to afford the title compound **S11** as a yellow oil (680 mg, 2.7 mmol, 29%).

R_f : 0.33 (5% MeOH in CH_2Cl_2)

ν_{max} (neat)/cm^{-1}: 3307 (m, N-H), 2934 (s, C-H), 2858 (s, C-H), 2115 (w, alkyne), 1692 (s, C=O), 1365 (C-H), 1248 (C-O)

^{1}H NMR (500 MHz, $CDCl_3$): δ 4.11 (d, J = 2.5 Hz, 2H), 3.49 (t, J = 6.5 Hz, 2H), 3.08, (t J = 7.5 Hz), 2.40 (t, J = 2.4 Hz, 1H), 1.60–1.55 (m, 2H), 1.49–1.35 (m, 2H), 1.42 (s, 9H), 1.38–1.29 (m, 4H) ppm

^{13}C NMR (126 MHz, $CDCl_3$): δ 156.0, 80.0, 79.0, 74.1, 70.0, 58.0, 40.5, 30.0, 29.3, 28.4, 26.5, 25.7 ppm

Note: Residual EtOAc was presented in ^{1}H NMR at 4.11 (q) (overlapping with 4.11 (d)), 2.02 (s) and 1.24 (t) ppm and in ^{13}C NMR at 171.1, 60.3, 21.0, 14.2 ppm; residual CH_2Cl_2 (transferring solvent) was presented in ^{1}H NMR at 5.28 (s) ppm and ^{13}C NMR at 53.4 ppm.

1.4.14 Synthesis of compound 14

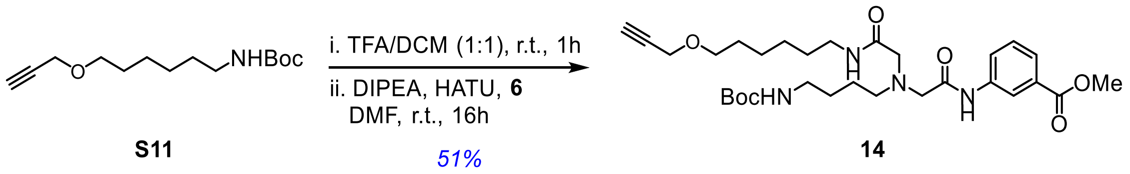

S11 **14**

tert-Butyl (6-(prop-2-yn-1-yloxy)hexyl)carbamate (**S11**, 200 mg, 0.78 mmol, 1.0 equiv) was dissolved in a mixture of TFA/CH_2Cl_2 1:1 (v/v, 4 mL) at 0 °C and stirred at room temperature for 1 hour. Upon completion (monitored by TLC, 5% MeOH in CH_2Cl_2) residual solvents were evaporated under stream of nitrogen, the residue was redissolved in DMF (5 mL) and the pH was adjusted to 7 using DIPEA. HATU (355 mg, 0.93 mmol, 1.2 equiv), DIPEA (289 µL, 1.68 mmol, 2.1 equiv) and **6** (348 mg, 0.80 mmol, 1.0 equiv) were added and the reaction mixture was stirred at room temperature for 16 hours. Upon completion (monitored by TLC, CH_2Cl_2/MeOH 10:1), the reaction mixture was diluted with CH_2Cl_2 (5 mL) and extracted with 10% w/v aqueous citric acid solution (3 × 3 mL), 0.3 M aqueous lithium chloride solution (3 × 3 mL) and brine (2 × 3 mL). The organic layer was combined, dried over $MgSO_4$, filtered and concentrated in vacuo. The crude product was purified by flash column chromatography (5% MeOH in CH_2Cl_2) to yield the title compound **14** as a yellow oil (229 mg, 0.40 mmol, 51% yield*).

R_f : 0.20 (5% MeOH in CH_2Cl_2)

HRMS (ESI): $[M+H]^+$ calc. for $C_{30}H_{47}O_7N_4^+$: 575.3439, found 575.3424

ν_{max} (neat)/cm^{-1}: 3289 (m, N-H), 2933 (m, C-H), 2860 (m, C-H), 2120 (w, alkyne), 1700 (s, C=O), 1660 (s, C=C), 1593 (m, C=C), 1530 (s, C=C), 1437 (m, C-H), 1365 (m, C-H) 1287 (m, C-O), 1248 (m, C-N), 1165 (w, C-O), 760 (s, C-H)

^{1}H NMR (500 MHz, $CDCl_3$): δ 8.28–8.27 (m, 1H), 7.91–7.89 (m, 1H), 7.79–7.77 (m, 1H), 7.40–7.37 (m, 1H), 4.12 (d, *J* = 2.4 Hz, 2H), 3.90 (s, 3H), 3.52–3.49 (m, 2H) **or** 3.53–3.50 (m, 2H), 3.32–3.28 (m, 2H) **or** 3.20–3.17 (m, 2H), 3.14–3.10 (m, 2H), 2.88 (s, 2H), 2.80 (s, 2H), 2.42 (t, *J* = 2.4 Hz, 1H), 1.70–1.52 (m, 16H), 1.41 (s, 9H), 1.38–1.34 (m, 6H) ppm

Note:* *Reported yield higher than actual. Residual DMF from the reaction is presented in ^{1}H NMR at 8.02 (s), 2.96 (s) and 2.88 (s) ppm. The unreacted linker* ***10* *is present in the ^{1}H NMR at 4.11 (d, C**H₂**-alkyne), 3.52–3.49 (m, CH₂-)* ***or*** *3.53-3.50 (m, CH₂-), 3.32–3.28 (m, -CH₂-)* ***or*** *3.20-3.17 (m, 2H, -CH₂), 2.41 (t, alkyne) 1.70-1.52 (m, -CH₂-) and 1.38-1.34 (m, -CH₂-). Route was abandoned following difficulties with purification.*

1.4.15 Synthesis of compound S12

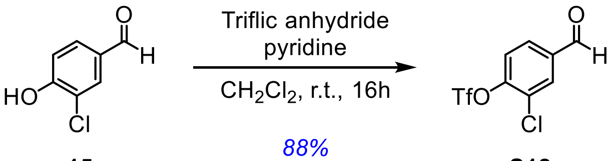

15 **S12**

To a stirring solution of 3-chloro-4-hydroxybenzaldehyde (**15**, 2.0 g, 13 mmol, 1.0 equiv) in anhydrous CH_2Cl_2 (100 mL) under nitrogen at 0 °C was added pyridine (1.7 mL, 21 mmol, 1.6 equiv). Trifluoromethanesulfonic anhydride (2.8 mL, 16 mmol, 1.2 equiv) was added dropwise over a period of 30 minutes. The solution was stirred at room temperature for 16 hours and upon completion (monitored by TLC, 20% EtOAc in hexane) was cooled down to 0 °C, quenched with ice water and basified to pH 8 with saturated $NaHCO_3$ solution. The organic layer was separated, and the aqueous layer was washed with CH_2Cl_2 (3 × 100 mL). The organic layers were combined, washed with brine (100 mL), and dried over $MgSO_4$ before being concentrated in vacuo to afford the title compound **S12** as a brown oil (3.3 g, 11 mmol, 88%) without further purification.

R_f : 0.42 (20% EtOAc in hexane)

LRMS (ESI): $[M+H]^+$ calc. for $C_8H_5ClF_3O_4S^+$: 288.6, found 288.4

^{1}H NMR (500 MHz, $CDCl_3$): δ 10.00 (s, 1H), 8.05 (d, J = 2.0 Hz, 1H), 7.88 (dd, J = 8.4, 2.0 Hz, 1H), 7.56 (d, J = 8.5 Hz, 1H) ppm

^{13}C NMR (126 MHz, $CDCl_3$): δ 188.9, 149.3, 136.5, 132.0, 129.4, 128.7, 123.8, 118.5 (q, $^1J_{C\text{-}F}$ = 320.7 Hz) ppm

^{19}F NMR (376 MHz, $CDCl_3$): δ −78.91 (s) ppm

Note: 10 µL trifluoroacetic acid used as ^{19}F NMR standard (75.55 (s)). Data were in accordance with those reported previously in literature.[5]

1.4.16 Synthesis of αD binder aldehyde 16

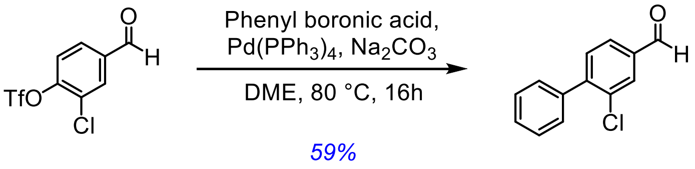

S12 **16**

To a stirring solution of 2-chloro-4-formylphenyl trifluoromethanesulfonate (**S12**, 3.3 g, 11 mmol, 1.0 equiv) in 1,2-dimethoxyethane (70 mL) were added phenylboronic acid (1.7 g, 14 mmol, 1.3 equiv) and Na_2CO_3 (3.5 g, 33 mmol, 3.0 equiv). The solution was degassed for 30 minutes under a stream of

N_2 before $Pd(PPh_3)_4$ (64 mg) was added and heated to reflux for 16 hours. The reaction mixture was cooled to room temperature, quenched with water (30 mL), and extracted with EtOAc (3 × 50 mL). The organic layers were combined, dried over $MgSO_4$, filtered, and concentrated in vacuo. The residue was purified by silica gel column chromatography (10% EtOAc in PE 40–60) to yield the title compound **16** as a white solid (1.4 g, 6.5 mmol, 59%).

R_f : 0.33 (10% EtOAc in PE 40-60)

HRMS (ESI): $[M+H]^+$ calc. for $C_{13}H_{10}OCl^+$: 217.0415, found 217.0407

^{1}H NMR (500 MHz, $CDCl_3$): δ 10.00 (s, 1H), 7.99 (d, *J* = 1.6 Hz, 1H), 7.83 (dd, *J* = 7.8, 1.6 Hz, 1H), 7.53 (d, *J* = 7.8 Hz, 1H), 7.48–7.42 (m, 5H) ppm

^{13}C NMR (126 MHz, $CDCl_3$): δ 190.6, 146.4, 138.2, 136.4, 133.7, 132.1, 131.1, 129.2, 128.4, 128.2, 127.8 ppm

Note: Data were in accordance with those reported previously in literature.[6]

1.4.17 Synthesis of ligand–linker precursor 17

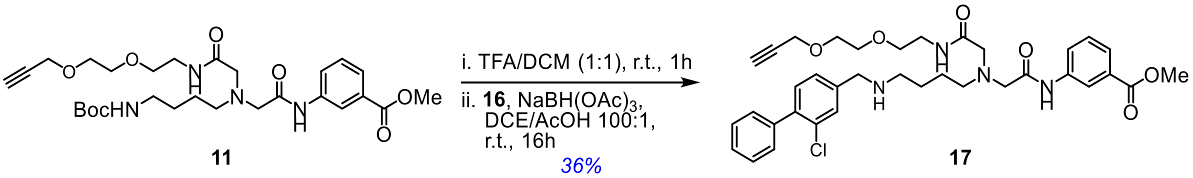

Methyl 3-(3-(4-((*tert*-butoxycarbonyl)amino)butyl)-5-oxo-9,12-dioxa-3,6-diazapentadec-14-ynamido)benzoate (**11**, 28 mg, 50 μmol, 1.0 equiv) was dissolved in a mixture of TFA/CH_2Cl_2 1:1 (v/v, 0.6 mL) at 0 °C for 1 hour. Upon completion (monitored by TLC, 5% MeOH in CH_2Cl_2), the residual solvents were evaporated under stream of nitrogen, the residue was redissolved in DCE (1 mL) and adjusted to pH 7 with DIPEA. A solution of **16** (14 mg, 64 μmol, 1.3 equiv) in DCE (1 mL) and acetic acid (10 μL) was stirred for 10 minutes at room temperature before the amine solution was added. The mixture was stirred for 2 hours, before $NaBH(OAc)_3$ (32 mg, 150 μmol, 3.0 equiv) was added in two portions over 30 minutes. The mixture was stirred for additional 16 hours. Upon completion (monitored by TLC, 5% MeOH in CH_2Cl_2) the reaction mixture was diluted with CH_2Cl_2 (5 mL) and extracted with 2 M aqueous $NaHCO_3$ solution (3 × 3 mL). The organic layers were combined, dried over $MgSO_4$, filtered and concentrated in vacuo. The crude product was purified by reversed phase column chromatography (C_{18} silica gel, 10–100% solvent B in solvent A. Solvent A: 5% formic acid solution (aq). Solvent B: MeCN) to yield the title compound **17** as a yellow oil (12 mg, 18 μmol, 36%).

R_f : 0.33 (5% MeOH in CH_2Cl_2)

HRMS (ESI): $[M+H]^+$ calc. for $C_{36}H_{44}O_6N_4Cl^+$: 663.2944, found 663.2933

$\boldsymbol{\nu}_{max}$ (neat)/cm^{-1}): 3287 (m, N-H), 3067 (w, N-H), 2924 (s, C-H), 2865 (s, C-H), 2115 (w, alkyne), 1719 (s, C=O), 1655 (s, C=C), 1592 (s, C=C), 1525 (s, C=C), 1479 (m C-H), 1442 (s, C-H), 1347 (w, C-H), 1288 (s, C-O), 1214 (s, C-O), 1101 (s, C-N), 1033 (w, C-O), 1007 (w), 753 (s, C-H)

^{1}H NMR (500 MHz, $CDCl_3$): δ 8.28–8.27 (m, 1H), 7.98–7.96 (m, 1H), 7.77–7.51 (m ,1H), 7.42–7.35 (m, 7H), 7.28–7.27 (m, 1H), 7.24–7.22 (m, 1H), 4.14 (d, *J* = 2.4 Hz, 2H), 3.89 (s, 3H), 3.77 (s, 2H), 3.66–3.62 (m, 4H), 3.58–3.56 (m, 2H), 3.53–3.50 (m, 2H), 3.34 (s, 2H), 3.29 (s, 2H), 2.70–2.64 (m, 4H), 2.43 (t, *J* = 2.4 Hz, 1H), 1.58–1.57 (m, 4H) ppm

^{13}C NMR (126 MHz, $CDCl_3$): δ 170.7, 169.7, 166.8, 141.0, 139.2, 139.09, 138.3, 132.3, 131.3, 130.8, 129.4, 129.4, 129.0, 128.0, 127.5, 126.6, 125.1, 124.1, 120.5, 79.4, 74.9, 69.9, 69.5, 69.1, 60.0, 59.0, 58.4, 56.2, 53.0, 52.1, 48.9, 39.1, 27.6, 25.2 ppm

1.4.18 Synthesis of compound S13

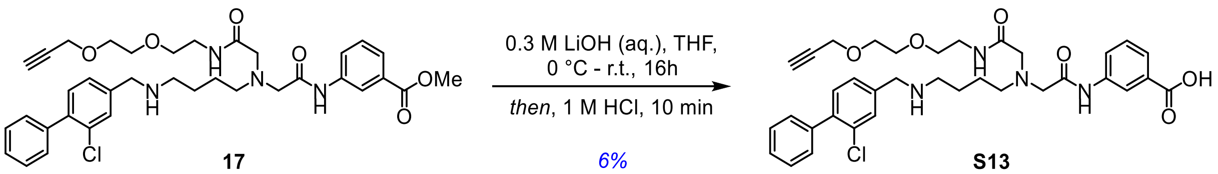

17 (12 mg, 26 µmol, 1.0 equiv) was dissolved in tetrahydrofuran (THF, 380 µL) and 0.3 M aqueous LiOH solution (290 µL, 87 µmol, 3.3 equiv) was added at 0 °C. The reaction mixture was stirred at room temperature for 16 hours before 1 M HCl (224 µL, 224 µmol) was added. The reaction was stirred for 10 min, concentrated in vacuo and purified by preparative HPLC to yield the title compound **S13** as a white solid (1 mg, 1.5 µmol, 6%).

R*f* : 0.02 (5% MeOH in CH_2Cl_2)

HRMS (ESI): $[M+H]^+$ calc. for $C_{35}H_{42}O_6N_4Cl^+$: 649.2787, found 649.2800

$\boldsymbol{\nu}_{max}$ (neat)/cm^{-1} : 3481 (br s, O-H), 2521 (m, C-H), 2159 (w, alkyne), 1735 (s, C=O), 1678 (s, C=C), 1374 (m, C-H), 1239 (s, C-O), 1204 (m, C-N), 1044 (s, C-O), 800 (s, C-H)

^{1}H NMR (500 MHz, d_6-DMSO): δ 8.35–8.32 (m, 1H), 8.19–8.12 (m, 2H), 7.87–7.85 (m, 1H), 7.67–7.57 (m, 2H), 8.13–7.08 (m, 6H), 4.06–4.03 (m, 2H), 3.83–3.78 (m, 4H), 3.10–3.06 (m, 4H), 2.75 (t, *J* = 6.8 Hz, 1H), 2.59–2.50 (m, 2H), 1.28–1.13 (m, 12H) ppm

^{13}C NMR (126 MHz, d$_6$-DMSO): δ 170.8, 170.4, 170.8, 145.3, 139.5, 130.6, 129.8, 125.9, 125.0, 124.49, 121.47, 120.3, 119.2, 116.6, 114.3, 81.1, 77.2, 59.5, 57.7, 55.8, 55.4, 52.7, 39.2, 29.5, 29.3, 26.3, 25.8, 24.2, 22.7 ppm

Crystalographic data

**PDB code**	9TTA
**Ligand**	S13
**Protein**	K74A
**Data Collection**	
Beamline	DIAMOND BEAMLINE i04-1
Wavelength (Å)	0.9179
Resolution range (Å)	58.17 - 1.91 (1.94-1.91)
Space group	P 1 21 1
Cell (a b c) (Å)	57.626 46.103 62.648
Cell (α β γ)(°)	90.0 111.8 90.0
Total reflections	408838 (18780)
Unique reflections	23838 (1098)
Multiplicity	17.2 (17.1)
Completeness (%)	99.7 (93.1)
Mean I/sigma(I)	6.1 (0.3)
R-merge	0.23146 (4.705)
R-pim	0.062 (1.161)
CC-half	0.9982 (0.320)
**Refinement**	
Refinement resolution range (Å)	58.17 - 1.91 (1.96-1.91)
No. reflections	22012 (1146)
No. reflections (Rfree)	1100 (53)
R-factor	0.22889 (0.428)
Rfree	0.28411 (0.452)
Number of total atoms	2975
atoms for macromolecules	2748
atoms for ligands	48
atoms for waters	179
Average B-factor	51.844
RMS(bonds) (Å)	0.01
RMS(bond angles) (°)	0.97
RMS(dihedral angles) (°)	3.29

1.4.19 Synthesis of ligand–linker precursor 18

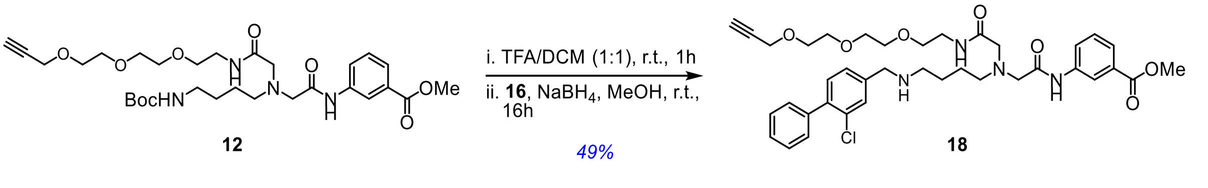

Prepared by *General Procedure C* using **12** (64 mg, 105 µmol, 1.0 equiv), TFA/CH_2Cl_2 1:1 (v/v, 1.5 mL), **16** (28 mg, 126 µmol, 1.2 equiv), $NaBH_4$ (5 mg, 137 µmol, 1.3 equiv) and MeOH (5 mL). The crude product was purified via reversed phase flash column chromatography (C_{18} silica gel, 10–100% solvent

B in solvent A. Solvent A: 5% formic acid solution (aq). Solvent B: MeCN) to yield the title compound **18** as a yellow oil (36 mg, 51 µmol, 49%).

R*f* : 0.21 (5% MeOH in CH_2Cl_2)

HRMS (ESI): $[M+H]^+$ calc. for $C_{38}H_{48}O_7N_4Cl^+$: 707.3206, found 707.3195

***v*max** (neat)/cm^{-1}: 3288 (m, N-H), 3063 (w, N-H), 2928 (s, C-H), 2863 (s, C-H), 2115 (w, alkyne), 1719 (s, C=O), 1659 (s, C=C), 1592 (s, C=C), 1536 (s, C=C), 1479 (m C-H), 1442 (s, C-H), 1346 (w, C-H), 1288 (s, C-O), 1215 (s, C-O), 1100 (s, C-N), 1033 (w, C-O), 753 (s, C-H)

^{1}H NMR (500 MHz, $CDCl_3$): δ 8.66 (s, 1H), 8.29–8.27 (t, 1H), 7.98–7.96 (m, 1H), 7.77–-7.75 (m, 1H), 7.43–7.35 (m, 7H), 7.29–7.27 (m, 1H), 7.25–7.24 (m, 1H), 6.89 (t, *J* = 5.0 Hz, 1H), 4.17 (d, *J* = 2.4 Hz, 2H), 3.89 (s, 3H), 3.79 (s, 2H), 3.69–3.55 (m, 12H), 3.34 (s, 2H), 3.30 (s, 2H), 2.70–2.66 (m, 4H), 2.43 (t, *J* = 2.4 Hz, 1H), 1.60–1.57 (m, 4H) ppm

^{13}C NMR (126 MHz, $CDCl_3$): δ 170.9, 169.9, 166.9, 140.7, 139.1, 138.8, 138.4, 132.4, 131.3, 130.8, 129.5, 129.4, 129.0, 128.0, 127.5, 126.7, 125.0, 124.2, 120.6, 79.3, 74.9, 70.5, 70.2, 70.0, 69.6, 69.1, 60.0, 58.9, 58.4, 56.2, 52.9, 52.1, 48.9, 39.1, 27.4, 25.2 ppm

1.4.20 Synthesis of ligand–linker precursor 19

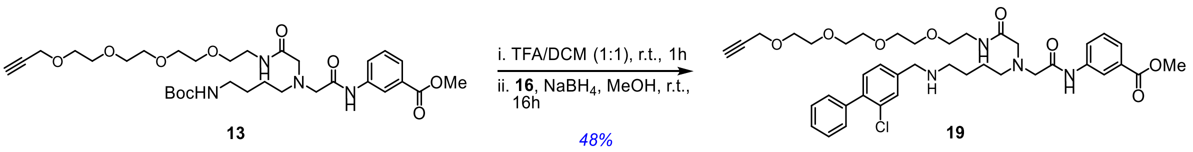

Prepared by *General Procedure C* using **13** (50 mg, 77 µmol, 1.0 equiv), TFA/CH_2Cl_2 1:1 (v/v, 1 mL), **16** (20 mg, 92 µmol, 1.2 equiv), $NaBH_4$ (4 mg, 100 µmol, 1.3 equiv) and MeOH (5 mL). The crude product was purified by reversed phase column chromatography (C_{18} silica gel, 10–100% solvent B in solvent A. Solvent A: 5% formic acid solution (aq). Solvent B: MeCN) to yield the title compound **19** as a yellow oil (28 mg, 37 µmol, 48%).

R*f* : 0.23 (5% MeOH in CH_2Cl_2)

HRMS (ESI): $[M+H]^+$ calc. for $C_{40}H_{52}O_8N_4Cl^+$: 751.3456, found 751.3468

***v*max** (neat)/cm^{-1}: 3296 (m, N-H), 2866 (s, C-H), 2115 (w, alkyne), 1719 (s, C=O), 1660 (s, C=C), 1593 (s, C=C), 1479 (m C-H), 1442 (s, C-H), 1347 (w, C-H), 1288 (s, C-O), 1215 (s, C-O), 1101 (s, C-N), 1033 (w, C-O), 753 (s, C-H)

^{1}H NMR (400 MHz, $CDCl_3$): δ 8.30–8.29 (m, 1H), 7.98–7.95 (m, 1H), 7.77–7.74 (m, 1H), 7.43–7.34 (m, 7H), 7.29–7.226 (m, 1H), 7.24–7.22 (m, 1H), 7.12-7.09 (m, 1H), 4.16 (d, *J* = 2.4 Hz, 2H), 3.89 (s, 3H),

3.77 (s, 2H), 3.69–3.54 (m, 14H), 3.52–3.48 (m, 2H), 3.34 (s, 2H), 3.30 (s, 2H), 2.70–2.63 (m, 4H), 2.43 (t, J = 2.4 Hz, 1H), 1.59–1.56 (m, 4H) ppm

^{13}C NMR (101 MHz, $CDCl_3$): δ 171.0, 170.0, 166.9, 141.1, 139.2, 139.0, 138.5, 132.3, 131.3, 130.8, 129.4, 128.9, 128.0, 127.5, 126.5, 124.9, 124.2, 120.6, 79.4, 74.8, 70.5, 70.4, 70.2, 69.7, 69.0, 60.0, 58.9, 58.3, 56.2, 53.0, 52.1, 49.0, 39.2, 27.6, 25.2 ppm

1.4.21 Synthesis of ligand–linker precursor 20

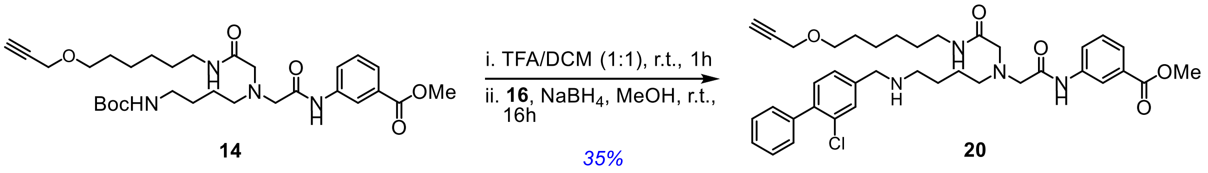

Prepared by *General Procedure C* using **14** (25 mg, 43 µmol, 1.0 uiv, TFA/CH_2Cl_2 1:1 (v/v, 0.5 mL), **16** (11 mg, 52 µmol, 1.2 equiv), $NaBH_4$ (2 mg, 56 µmol, 1.3 equiv) and MeOH (5 mL). The crude product was purified by reversed phase column chromatography (C_{18} silica gel, 10–100% solvent B in solvent A. Solvent A: 5% formic acid solution (aq). Solvent B: MeCN) to yield the title compound **20** as a yellow oil (10 mg, 15 µmol, 35%).

R_f : 0.11 (5% MeOH in CH_2Cl_2)

HRMS (ESI): $[M+H]^+$ calc. for $C_{38}H_{48}ClN_4O_5^+$: 675.3394, found 675.3391

***ν*$_{max}$** (neat)/cm^{-1}: 3292 (m, N-H), 2860 (m, C-H), 2115 (w, alkyne), 1734 (s, C=O), 1696 (s, C=O), 1694 (s, C=C), 1676 (m, C=C), 1577 (s, C=C), 1423 (m, C-H), 1373 (m, C-H) 1236 (m, C-O), 1236 (m, C-N), 1123 (w, C-O), 760 (s, C-H)

^{1}H NMR (500 MHz, MeOD): δ 8.36–8.31 (m, 1H), 7.87–7.23 (m, 2H), 7.49–7.31 (m, 9H), 4.12–4.05 (m, 2H), 3.91–3.85 (m, 3H), 3.51–3.41 (m, 4H), 3.26–3.22 (m, 2H), 2.94–2.91 (m, 1H) 2.81–2.78 (m, 1H), 2.71–2.67 (m, 2H), 1.80–1.29 (m, 18H) ppm

^{13}C NMR (126 MHz, $CDCl_3$): δ 172.8, 171.6, 166.7, 142.7, 139.3, 139.2, 138.3, 132.2, 131.4, 131.2, 130.4, 129.0, 128.9, 127.7, 127.7, 127.4, 127.2, 125.0, 124.9, 124.1, 120.3, 79.5, 74.2, 69.4, 67.4, 59.1, 58.3, 55.8, 51.3, 39.0, 29.4, 29.0, 28.9, 26.4, 26.2, 25.5, 24.6, 22.3 ppm

1.4.22 Synthesis of compound S15

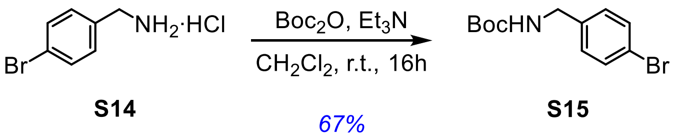

To a stirring suspension of 4-bromobenzylamine hydrochloride (**S14**, 3.0 g, 13.5 mmol, 1.0 equiv) in CH_2Cl_2 (30 mL), Et_3N (3.8 mL, 27.0 mmol, 2.0 equiv) was added, followed by Boc_2O (3.25 g, 14.9 mmol, 1.1 equiv). The resulting solution was stirred for 16 hours at room temperature, after which LC–MS analysis indicated absence of the starting material. The reaction mixture was diluted with CH_2Cl_2 (30 mL), and the organic layer was washed with aqueous saturated NH_4Cl solution (2 × 40 mL) followed by brine (40 mL). The organic layer was then dried over anhydrous $MgSO_4$, filtered, and the solvent was removed in vacuo to yield the title compound **S15** as a white solid (2.54 g, 8.88 mmol, 67%).

R_f : 0.45 (20% EtOAc in PE)

^{1}H NMR (500 MHz, $CDCl_3$): δ 7.41 (d, J = 8.4 Hz, 1H), 7.13 (d, J = 8.3 Hz, 2H), 5.00 (br s, 1H), 4.23 (d, J = 5.6 Hz, 2H), 1.43 (s, 9H) ppm

^{13}C NMR (126 MHz, $CDCl_3$): δ 155.9, 138.1, 131.6, 129.1, 121.1, 79.7, 44.0, 26.41 ppm

Note: Data are in accordance with those reported previously in literature.[7]

1.4.23 Synthesis of compound S16

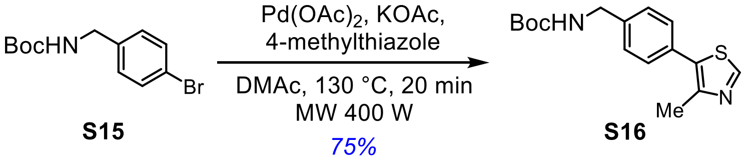

A suspension of **S15** (1.00 g, 3.49 mmol, 1.0 equiv), $Pd(OAc)_2$ (79 mg, 0.35 mmol, 0.1 equiv), 4-methylthiazole (637 µL, 7.00 mmol, 2.0 equiv) and KOAc (687 mg, 7.00 mmol, 2.0 equiv) in *N,N*-dimethylacetamide (3.5 mL), was prepared in a 2.0–5.0 mL Biotage® Microwave Reaction Vial. The solution was purged with dry N_2 for 1 minute before the tube was sealed. The sample was then placed inside a Biotage® Initiatior$^+$ microwave unit and irradiated at 400 W and 130 °C for 20 min. Then, the mixture was cooled to room temperature and diluted with H_2O (15 mL). The aqueous layer was extracted with CH_2Cl_2 (3 × 15 mL). The combined organic layer was washed with brine (2 × 15 mL). The organic layer was then dried over anhydrous $MgSO_4$, filtered, and concentrated in vacuo. The crude product was purified by flash column chromatography (SiO_2, 0–50% EtOAc in PE 40–60) to give the title compound **S16** as a yellow solid (840 mg, 2.76 mmol, 75%).

^{1}H NMR (400 MHz, MeOD): δ 8.78 (1H, s), 7.33–7.27 (4H, app. q, J = 8.5 Hz, 4H), 4.18 (s, 2H), 2.37 (s, 3H), 1.36 (s, 9H) ppm

^{13}C NMR (101 MHz, MeOD): δ 157.2, 151.5, 147.6, 140.0, 132.1, 130.0, 129.4, 127.3, 78.9, 43.3, 27.4, 14.5 ppm

Note: Data are in accordance with those reported previously in literature.[7]

1.4.24 Synthesis of compound S17

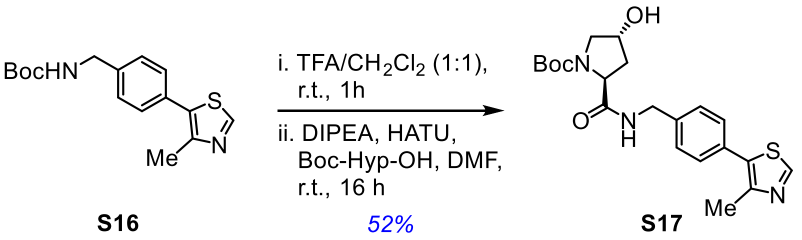

S16   **S17**

S16 (800 mg, 2.63 mmol, 1.0 equiv) was dissolved in a solution of and TFA/CH_2Cl_2 1:1 (v/v, 6 mL). The solution was stirred for 1 hour at room temperature before being concentrated in vacuo. The residue was dissolved in DMF (15 mL), and Boc-L-hydroxyproline (605 mg, 2.63 mmol, 1.0 equiv) was added. To the stirring mixture, DIPEA (1.80 mL, 10.5 mmol, 4.0 equiv) was added, followed by HATU (1.10 g, 2.90 mmol, 1.2 equiv). The reaction was stirred for 16 hours at room temperature, after which LC–MS indicated absence of the starting material. The reaction mixture was diluted with H_2O (100 mL) and extracted in EtOAc (3 × 50 mL). The combined organic layers were washed with 5% w/v aqueous LiCl solution (5 × 50 mL). The organic layer was then washed with brine (50 mL), dried over anhydrous $MgSO_4$, filtered, and concentrated in vacuo. The crude product was purified *via* automated reversed-phase column chromatography (5–95% acetonitrile in 1% aqueous formic acid) to give the title compound **S17** as a white solid (571 mg, 1.37 mmol, 52%).

^{1}H NMR (500 MHz, $CDCl_3$): δ 8.67 (s, 1H), 7.37 (d, J = 8.1 Hz, 2H), 7.32 (d, J = 7.8 Hz, 2H), 4.57 – 4.31 (m, 4H), 3.67 – 3.45 (m, 2H), 2.90 (br s, 1H), 2.50 (s, 3H), 2.07 (br s, 1H), 1.42 (br s, 9H) ppm

^{13}C NMR (126 MHz, $CDCl_3$): δ 150.4, 148.4, 138.1, 131.6, 129.6, 128.2, 127.8, 80.9, 70.0, 58.8, 54.8, 43.0, 37.0, 27.9, 16.0 ppm

Note: Data are in accordance with those reported previously in literature.[7]

1.4.25 Synthesis of VHL Boc 21

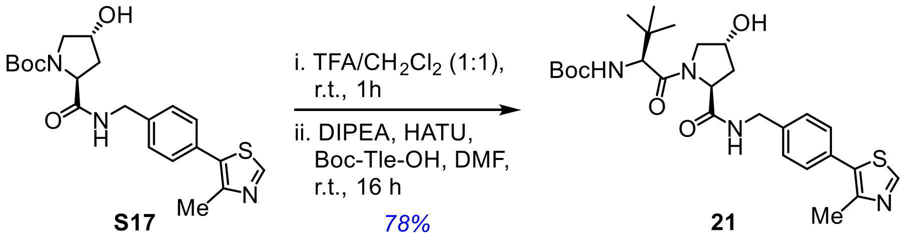

S17 (500 mg, 1.20 mmol, 1.0 equiv) was dissolved in a solution of TFA/CH_2Cl_2 1:1 (v/v, 5 mL). The solution was stirred for 1 hour at room temperature before being concentrated in vacuo. The residue was dissolved in DMF (20 mL) and Boc-ʟ-*tert*-leucine (278 mg, 1.20 mmol, 1.0 equiv) was added. To the stirring mixture, DIPEA (0.84 mL, 4.80 mmol, 4.0 equiv) was dropwise added, followed by HATU (502 mg, 1.32 mmol, 1.1 equiv). The reaction was stirred for 16 hours at ambient temperature. The reaction mixture was diluted with H_2O (50 mL) and extracted in EtOAc (3 × 25 mL). The organic layers were combined and washed with a 5% w/v aqueous LiCl solution (5 × 25 mL) and brine (25 mL), dried over anhydrous $MgSO_4$, and concentrated in vacuo. The crude product was purified via automated reversed-phase column chromatography (5–95% acetonitrile in 1% aqueous formic acid) to give the title compound **21** as a white solid (497 mg, 0.94 mmol, 78%).

^{1}H NMR (700 MHz, $CDCl_3$): δ 8.74 (s, 1H), 7.44 (t, *J* = 7.4 Hz, 1H), 7.36 – 7.33 (app. q, *J* = 7.34 Hz, 4H), 5.18 (d, *J* = 9.1 Hz, 1H), 4.45 (t, *J* = 7.9 Hz, 1H), 4.57 (dd, *J* = 14.9, 6.7 Hz, 1H), 4.38 – 4.33 (m, 1H), 4.32 (dd, *J* = 15.0, 5.1 Hz, 1H), 4.15 (d, *J* = 9.2 Hz, 1H), 4.08 (app. d, *J* = 11.4 Hz, 1H), 3.57 (dd, *J* = 11.4, 3.4 Hz, 1H), 2.57 – 2.53 (m, 1H), 2.14 – 2.11 (s, 3H), 1.90 (m, 1H), 1.38 (s, 9H), 0.93 (s, 9H) ppm

^{13}C NMR (176 MHz, $CDCl_3$): δ 172.8, 170.7, 156.5, 150.5, 147.9, 138.3, 132.0, 130.6, 129.5, 128.2, 80.5, 70.1, 58.9, 58.3, 56.5, 43.3, 35.7, 34.7, 28.3, 26.4, 15.8 ppm

Note: Data are in accordance with those reported previously in literature.[7]

1.4.26 Synthesis of VHL azide 22

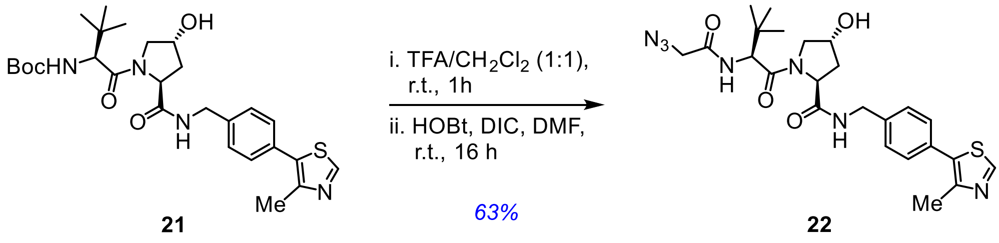

Prepared by *General Procedure B* using **21** (400 mg, 0.75 mmol, 1.0 equiv), TFA/CH_2Cl_2 1:1 (v:v, 4 mL), 2-azidoacetic acid (91 mg, 0.90 mmol, 1.2 equiv), HOBt (151 mg, 0.98 mmol, 1.3 equiv), DIC (155 µL,

0.98 mmol, 1.3 equiv) and DMF (10 mL). The crude product was purified via reversed phase flash column chromatography (C_{18} silica gel, 10–100% solvent B in solvent A. Solvent A: 5% formic acid solution (aq). Solvent B: MeCN) to yield the title compound **22** as a white solid (242 mg, 0.47 mmol, 63%).

R_f : 0.35 (5% MeOH in CH_2Cl_2)

HRMS (ESI): [M] calc. for $C_{24}H_{31}N_7O_4S$: 513.2158 found 513.2163

***ν*max** (neat)/cm^{-1}: 3650 (m, O-H), 2988 (w, C-H), 2940 (w, C-H), 2095 (w, N=C=S), 1734 (s, C=O), 1694 (s, C=O), 1573 (m, C=C), 1676 (s, C=O), 1423 (w, C-H), 1373 (m, C-H), 1236 (m, C-N), 1203 (m, C-N), 1171 (s, C-O), 1008 (w, C=C), 832 (w, C-H), 798 (s, C-H), 717 (w, C-H)

^{1}H NMR (500 MHz, $CDCl_3$): δ 8.79 (s, 1H), 7.40–7.35 (m, 4H), 7.23 (t, *J* = 5.9 Hz), 6.89 (d, *J* = 8.6 Hz, 1H), 4.74–4.71 (m, 1H), 4.62 – 4.55 (m, 2H), 4.49 (d, *J* = 8.7 Hz, 1H), 4.37–4.33 (m, 1H), 4.09–3.94 (m, 3H), 3.63 (dd, *J* = 11.3, 3.8 Hz, 1H), 2.63–2.58 (m, 1H), 2.56 (s, 3H), 2.15–2.10 (m, 1H), 0.95 (s, 9H) ppm

^{13}C NMR (126 MHz, $CDCl_3$): δ 171.2, 170.5, 167.0, 151.9, 147.0, 140.6, 133.5, 129.49, 129.44, 70.2, 58.5, 57.5, 56.7, 52.5, 43.3, 35.8, 35.1, 26.4 ppm

1.4.27 Synthesis of final VHL PROTAC 23

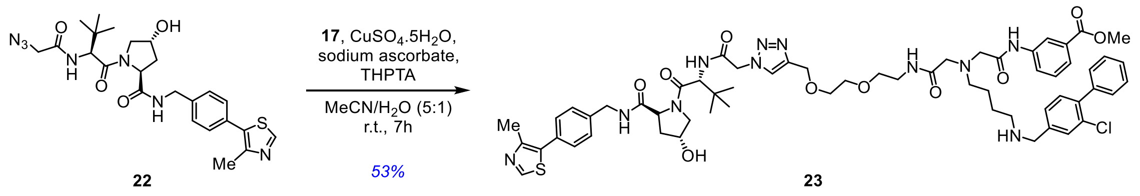

Prepared by *General Procedure D* using **17** (11 mg, 16 µmol, 1.0 equiv), **22** (8 mg, 16 µmol, 1.0 equiv), THPTA (14 mg, 32 µmol, 2.0 equiv), sodium ascorbate (19 mg, 96 µmol, 6.0 equiv), and $Cu_2SO_4{\cdot}5H_2O$ (8 mg, 32 µmol, 2.0 equiv). The reaction was purified via by HPLC (method: 25–65% B, 20 min) to yield the title compound **23** as a white solid (10 mg, 8.5 µmol, 53%).

R_f : 0.25 (5% MeOH in CH_2Cl_2)

HRMS (ESI): [M] calc. for $C_{60}H_{74}ClN_{11}O_{10}S$: 1175.5029, found 1175.5031

***ν*max** (neat)/cm^{-1}: 3650 (m, O-H), 2488 (w, C-H), 2234 (w, C-H), 2095 (w, N=C=S), 1734 (s, C=O), 1692 (s, C=O), 1373 (m, C-H), 1238 (m, C-N), 1203 (m, C-N), 1169 (s, C-O), 1131 (w, C=C), 1082 (w, C=C), 967 (w, C-H), 895 (s, C-H), 818 (w, C-H)

^{1}H NMR (500 MHz, MeOD): δ 8.91 (br s, 1H), 8.31–8.28 (m, 1H), 7.99 (br s, 1H), 7.77–7.76 (m, 2H), 7.69 (br s, 1H), 7.50–7.33 (m, 12H), 5.31–5.21 (m, 2H), 4.63–4.52 (m, 5H), 4.36–4.33 (m, 1H), 4.26 (s,

2H), 3.88 (s, 3H), 3.83–3.54 (m, 11H), 3.45–3.43 (m, 2H), 3.35 (s, 1H), 2.46 (s, 3H), 2.21–2.18 (m, 1H), 2.09–2.03 (m, 1H) 1.83–1.77 (m, 8H), 1.28 (s, 9H), 0.91–0.88 (m, 2H) ppm

^{13}C NMR (126 MHz, MeOD): δ 172.9, 170.4, 166.6, 166.3, 151.6, 147.4, 144.4, 141.6, 138.9, 138.4, 138.1, 132.6, 132.2, 131.7, 131.1, 130.8, 130.0, 128.9, 128.9, 128.4, 127.8, 127.7, 127.6, 125.5, 125.1, 124.0, 120.5, 69.7, 69.6, 69.4, 69.3, 67.6, 63.4, 59.7, 59.5, 58.3, 57.3, 56.6, 55.7, 51.4, 51.3, 50.1, 42.9, 42.3, 40.0, 39.0, 37.6, 36.1, 35.2, 33.5, 31.7, 29.3, 29.2, 29.0, 28.8, 25.6, 23.6, 22.9, 22.3, 14.3 ppm

1.4.28 Synthesis of final VHL PROTAC 24

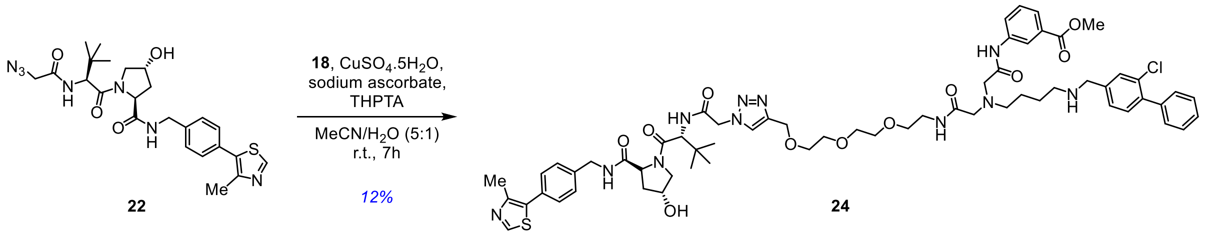

Prepared by *General Procedure D* using **18** (15 mg, 21 µmol, 1.0 equiv), **22** (11 mg, 21 µmol, 1.0 equiv), THPTA (18 mg, 42 µmol, 2.0 equiv), sodium ascorbate (25 mg, 126 µmol, 6.0 equiv), $Cu_2SO_4{\cdot}5H_2O$ (11 mg, 42 µmol, 2.0 equiv). The reaction was indicated as completed by absence of starting material on LC–MS. The reaction mixture was concentrated in vacuo and purified via by HPLC (method: 25–65% B, 20 min) to yield the title compound **24** as a white solid (3 mg, 2.5 µmol, 12%).

R_f : 0.16 (5% MeOH in CH_2Cl_2)

HRMS (ESI): $[M+H]^+$ calc for $[C_{62}H_{78}N_{11}O_{11}S_1Cl+H]^+$: 1220.5370, found 1220.5364

v_{max} (neat)/cm^{-1}: 3634 (m, O-H), 2488 (w, C-H), 2239 (w, C-H), 2081 (w, N=C=S), 1743 (s, C=O), 1670 (s, C=O), 1319 (m, C-H), 1249 (m, C-N), 1201 (m, C-N), 1166 (s, C-O), 1082 (w, C=C), 961 (w, C-H), 909 (s, C-H)

^{1}H NMR (700 MHz, $CDCl_3$): δ 8.68 (s, 1H), 7.83 – 7.70 (br m, 2H), 7.76 – 7.53 (br m, 2H), 7.45–7.29 (m, 9H), 5.43 – 5.39 (br m, 5H), 5.35 (app. d, *J* = 4.87 Hz, 2H), 4.05 – 4.02 (m, 4H), 3.88 (s, 3H), 3.75–3.51 (m, 12H), 2.44 (s, 3H), 2.37 – 2.34 (m, 2H), 2.25 – 2.21 (m, 5H) 1.83–1.77 (br m, 4H), 2.18 (s, 1H), 2.06 – 1.98 (m, 5H), 1.09 (s, 9H) ppm

Note: Yield too low to obtain a well resolved ^{1}H and ^{13}C NMR spectra due to poor HPLC purification. The compound was carried forward to in vitro testing.

1.4.29 Synthesis of final VHL PROTAC 25

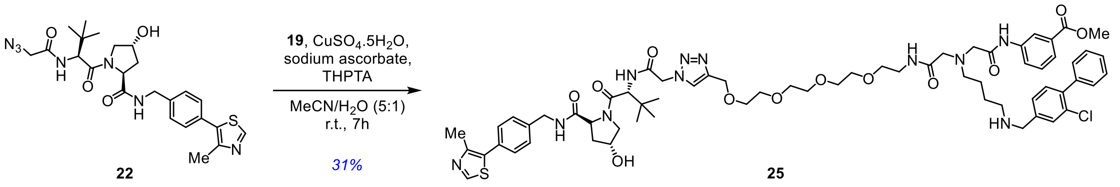

Prepared by General Procedure D using **19** (20 mg, 26 µmol, 1.0 equiv), **22** (13 mg, 26 µmol, 1.0 equiv), THPTA (23 mg, 52 µmol, 2.0 equiv), sodium ascorbate (31 mg, 156 µmol, 6.0 equiv), $Cu_2SO_4{\cdot}5H_2O$ (13 mg, 52 µmol, 2.0 equiv). The reaction mixture was concentrated in vacuo and purified via by HPLC (method: 25–65% B, 20 min) to yield the title compound **25** as a white solid (10 mg, 8 µmol, 31%).

R_f : 0.13 (5% MeOH in CH_2Cl_2)

HRMS (ESI): $[M+H]^+$ calc for $[C_{64}H_{82}N_{11}O_{12}S_1Cl+H]^+$: 1264.5632, found 1264.5669

ν

$\boldsymbol{\nu}_{\mathbf{max}}$ (neat)/cm^{-1}: 3130 (m, O-H), 2892 (s, C-H), 2488 (w, C-H), 2252 (w, C-H), 2078 (w, N=C=S), 1720 (s, C=O), 1671 (s, C=O), 1326 (m, C-H), 1253 (w, C-N), 1166 (s, C-O), 1079 (w, C=C), 965 (w, C-H), 910 (s, C-H)

^{1}H NMR (700 MHz, $CDCl_3$): δ 8.91 (s, 1H, H^1), 8.33 (br s, 1H, H^{24}), 8.16 (br s, 1H, H^{17}) 7.76 (d, *J* = 7.56 Hz, 1H, H^{23}), 7.70 (br s, 1H, H^{21}), 7.58 (s, 1H, H^{22}), 7.45–7.31 (m, 10H), 7.24–7.23 (m, 2H), 5.01 (br d, 1H, O-**H**), 4.80 (br s, 1H, H^{15}), 4.60 – 4.54 (m, 4H), 4.47 (s, 2H, H^8), 4.40 – 4.36 (m, 3H), 4.27 (br s, 1H, H^{11}), 4.16 – 4.01 (m, 4H), 3.88 (s, 3H, H^{19}), 3.74–3.51 (m, 17H, likely O-C**H**$_2$), 2.46 (s, 3H, H^3), 2.29 – 2.23 (m, 2H), 2.17 – 2.08 (m, 4H), 1.83 –1.77 (br m, 4H), 1.03 (s, 9H, H^{14}), 0.84 (br s, 2H) ppm

^{13}C NMR (176 MHz, $CDCl_3$): δ 171.7, 171.2, 166.8, 162.9, 161.3, 160.1, 151.4, 146.2, 141.7, 139.5, 138.4, 137.6, 135.7, 133.1, 133.0, 132.0, 131.6, 131.1, 130.9, 129.3, 129.2, 128.2, 126.0, 124.6, 121.1, 116.8, 115.1, 70.3, 70.1, 69.6, 64.0, 59.6, 58.5, 57.6, 52.3, 47.7, 46.5, 42.8, 39.7, 37.8, 35.4, 31.9, 26.5, 14.9 ppm

Note: Grease present in NMR: (^{1}H: 1.26 (s), 0.88 (m) δ ppm.; ^{13}C: 29.7 δ ppm).

1.4.30 Synthesis of final VHL PROTAC 26

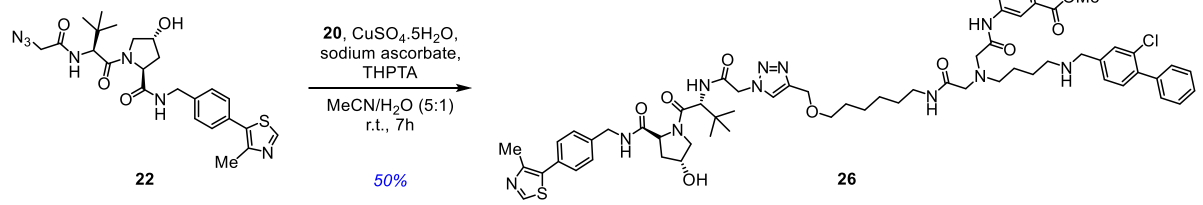

Prepared by *General Procedure D* using **20** (17 mg, 24 µmol, 1.0 equiv), **22** (12 mg, 24 µmol, 1.0 equiv) THPTA (21 mg, 48 µmol, 2.0 equiv), sodium ascorbate (29 mg, 144 µmol, 6.0 equiv) $Cu_2SO_4{\cdot}5H_2O$

(12 mg, 48 μmol, 2.0 equiv). The reaction mixture was concentrated in vacuo and purified via by HPLC (method: 25–65% B, 20 min) to yield the title compound **26** as a white solid (15 mg, 12 μmol, 50%).

R$_f$: 0.09 (10% MeOH in CH_2Cl_2)

HRMS (ESI): $[M+H]^+$ calc for $[C_{62}H_{78}N_{11}O_9S_1Cl+H]^+$: 1188.5471, found 1188.5466

***v*$_{max}$** (neat)/cm^{-1}: 3254 (w, O-H), 2962 (w, C-H), 2863 (w, C-H), 1666 (s, C=O), 1561 (m, C-O), 1443 (m, C-H), 1298 (m, C-N), 1199 (s, C-O), 1131 (s, C-O) 1082, (m C=C), 3130 (m, O-H), 2892 (s, C-H), 2488 (w, C-H), 2252 (w, C-H), 1720 (s, C=O), 1671 (s, C=O), 1326 (m, C-H), 1253 (w, C-N), 1166 (s, C-O), 1079 (w, C=C), 965 (w, C-H), 910 (s, C-H)

^{1}H NMR (700 MHz, d$_6$-DMSO): δ 8.99 (s, 1H), 8.61 (app t, *J* = 6.1 Hz, 1H), 8.54 (app. d, *J* = 1.5 Hz, 1H), 8.32 (app. t, *J* = 1.8 Hz, 1H), 8.00 (s, 1H), 7.80 (d, *J* = 8.0 Hz, 1H), 7.74 (m, 1H), 7.70 (t, *J* = 4.5 Hz, 1H), 7.53 – 7.47 (m, 4H), 7.45 – 7.38 (m, 7H), 5.22 (q, *J* = 14.4 Hz, 2H), 4.53 (d, *J* = 9.3 Hz, 1H), 4.46 – 4.37 (m, 4H), 4.33 (app s, 1H), 4.23 – 4.20 (m, 4H), 3.85 (s, 3H), 3.66 (dd, *J* = 10.5 Hz, 4.0 Hz, 1H), 3.57 (d, *J* = 10.6 Hz, 1H), 3.40 (t, *J* = 6.6 Hz, 2H), 3.12 (app. q, *J* = 6.5 Hz, 2H), 2.97 (app. s, 2H), 2.45 (s, 3H), 2.06 – 2.03 (m, 1H), 1.92 – 1.88 (m, 1H), 1.67 (br s, 4H), 1.48 – 1.46 (m, 2H), 1.42 – 1.41 (m, 2H), 1.27 – 1.26 (m, 4H), 0.96 (s, 9H) ppm

^{13}C NMR (126 MHz, d$_6$-DMSO): δ 172.3, 169.5, 167.5, 166.5, 166.4, 165.8, 151.9, 148.2, 144.2, 140.8, 140.0, 139.0, 138.5, 133.7, 132.2, 132.1, 131.8, 131.7, 131.6, 130.7, 130.1, 129.6, 129.5, 129.3, 129.1, 128.8, 128.5, 127.9, 125.9, 125.0, 124.3, 120.3, 117.6, 116.0, 70.0, 69.3, 67.9, 63.7, 59.2, 57.3, 57.0, 55.5, 52.8, 51.7, 49.5, 46.7, 42.1, 39.1, 38.4, 36.1, 29.5, 29.3, 26.8, 26.7, 26.6, 25.8, 16.4, 14.4, 11.3 ppm

1.4.31 Synthesis of CK2 precursor 27

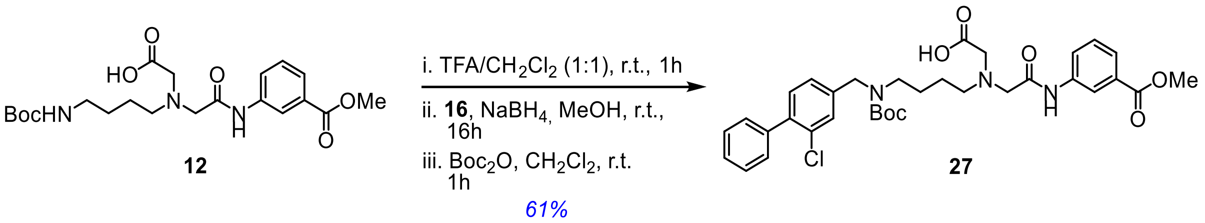

Prepared by General Procedure C using **12** (1.00 g, 2.29 mmol, 1.0 equiv), TFA/CH_2Cl_2 1:1 (v/v, 4.0 mL), **16** (594 mg, 2.74 mmol, 1.2 equiv), $NaBH_4$ (113 mg, 2.98 mmol, 1.3 equiv) and MeOH (5 mL). The crude product was purified via reversed phase flash column chromatography (C_{18} silica gel, 10–100% solvent B in solvent A. Solvent A: 5% formic acid solution (aq). Solvent B: MeCN) to yield the intermediate amine salt, which was dissolved in CH_2Cl_2 (8 mL) and the pH was adjusted to 7 by

dropwise addition of DIPEA. To the stirring solution was added Boc_2O (600 mg, 2.75 mmol, 1.2 equiv) and the mixture was left to stir for 1 hour at room temperature, after which LC–MS indicated an absence of starting material. The reaction mixture was concentrated and purified via basic combi flash column chromatography (C_{18} silica gel, 10–100% solvent B in solvent A. Solvent A: 2.5 % NH_4OH solution (aq). Solvent B: MeCN) to yield the title compound **27** as a white solid (0.89 g, 1.39 mmol, 61%).

R_f : 0.34 (5% MeOH in CH_2Cl_2)

HRMS (ESI): $[M+H]^+$ calc. for $C_{34}H_{41}O_7N_3Cl^+$: 638.2633, found 638.2616

ν_{max} (neat)/cm^{-1}): 2979 (br, w C-H), 1805 (m, s C=O), 1754 (m, s C=O), 1720 (s, s C=O acid), 1687 (s, s C=O), 1655 (s, s C=C), 1638 (m, s C=C), 1626 (m, s C=C), 1479 (m, s C-H), 1289 (s, s C-O), 1211 (s, s C-O or C-N), 1163 (s, s C-O or C-N), 1140 (s, s C-O or C-N), 1069 (s, s C-O or C-N).

^{1}H NMR (500 MHz, $CDCl_3$): δ 10.1 (s, 1H, -N**H**), 8.16 (appt. br s, 1H, H^4), 7.92 (app br s, 1H, H^6), 7.73 (app br d, J = 8.68 Hz, 1H, H^8), 7.41 – 7.33 (m, 7H, $H^{7/25/21/22/23}$), 7.29–7.27 (m, 1H, H^{17}), 7.25 – 7.24 (m, 1H, H^{24}), 7.10 (br s, 1H, -N**H**), 4.39 (br d, 2H, H^{26}), 3.87 (s, 3H, H^1), 3.70 (br s, 2H, H^{15}), 3.59 (br s, 2H H^{10}), 3.28 – 3.15 (m, 2H, $H^{11 \text{ or } 14}$), 2.95 – 2.48 (m, 2H, $H^{11 \text{ or } 14}$), 1.60–1.57 (m, 4H, $H^{12/13}$) 1.52 (s, 9H, H^{35}) ppm

^{13}C NMR (126 MHz, $CDCl_3$): δ 172.57, 168.1, 166.8, 155.8, 146.8, 140.7, 139.4, 139.1, 137.9,137.9, 132.6, 132.5, 131.8, 130.8, 129.4, 129.2, 128.5, 128.0, 127.6, 125.4, 120.6, 85.2, 56.5, 55.6, 52.3, 49.8, 46.1, 31.2, 28.7, 28.4, 27.4 ppm

1.4.32 Synthesis of final CRBN PROTAC 28

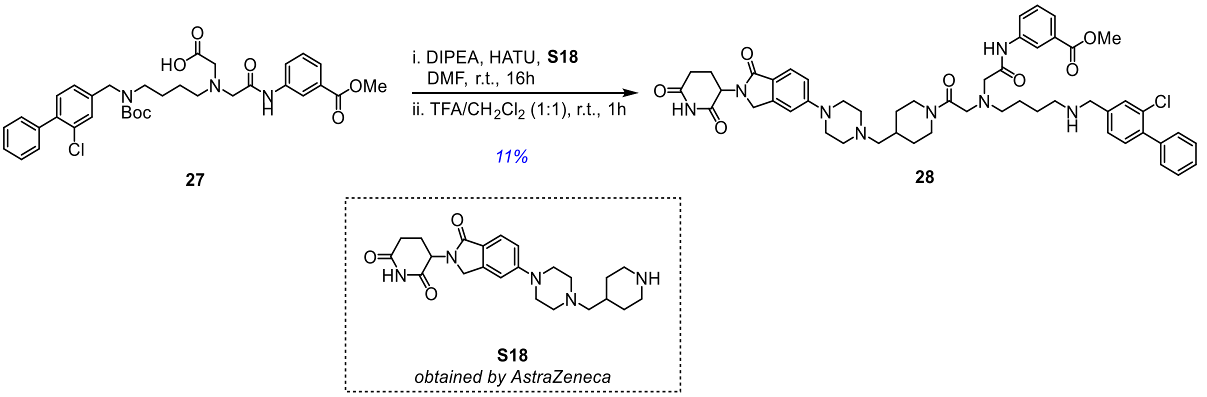

27 (20 mg, 31 µmol, 1.0 equiv) was dissolved in DMF (1 mL) and **S18** (13 mg, 31 µmol, 1.0 equiv) was added. To the stirring mixture, DIPEA (22 µL, 124 µmol, 4.0 equiv) was added, followed by HATU (13 mg, 34 µmol, 1.1 equiv). The reaction mixture was stirred for 16 hours at room temperature, after which LC–MS indicated an absence of starting material. The reaction mixture was concentrated in vacuo and redissolved in TFA/CH_2Cl_2 1:1 (v/v, 1.5 mL). The mixture was stirred at room temperature

for 1 hour, before being concentrated in vacuo. The reaction was purified *via* RP-HPLC (method: 25–95% B, 20 min) to yield the title compound **28** as a white solid (3 mg, 3.3 µmol, 11%).

R*f* : 0.12 (5% MeOH in CH_2Cl_2)

HRMS (ESI): $[M+H]^+$ calc. for $[C_{52}H_{61}O_7N_8Cl+H]^+$: 945.4430, found 945.4398

$\boldsymbol{\nu}_{max}$ (neat)/cm^{-1}: 1697 (m, C=O), 1685, (m, C=O), 1663 (s, C=C), 1654 (s, C=C), 1637 (s, C=C), 1559 (s, N-H or C-N), 1541 (s, N-H or C-N), 1534 (s, N-H or C-N), 1522 (s, N-H or C-N), 1508 (m, C=C), 1475 (m, C=C), 1400 (C-H), 1253 (w, C-O or C-N), 1202 (w, C-O or C-N), 1177 (w, C-O or C-N)

^{1}H NMR (700 MHz, d_6-DMSO): δ 8.33 (app. t, *J* = 1.7 Hz, 1H), 7.95 (s, 1H), 7.82 (d, *J* = 1.4 Hz, 1H), 7.74 (d, *J* = 1.4 Hz, 1H), 7.69 (d, J = 7.6 Hz, 1H), 7.60 (d, *J* = 8.5 Hz, 1H), 7.53 – 7.41 (m, 7H) 7.21 – 7.14 (m, 2H), 5.06 (dd, *J* = 13.3, 5.1 Hz, 1H), 4.36 (d, *J* = 17.0 Hz, 2H), 4.24 (d, *J* = 17.2 Hz, 2H), 4.22 – 4.21 (br s, 1H), 4.03 – 3.98 (br m, 2H), 3.85 (s, 3H), 3.68 – 3.43 (m, 11H), 3.24 – 2.90 (m, 10H), 2.62 – 2.58 (m, 1H), 2.38 (qd, *J* = 26.4, 13.2, 4.6 Hz, 1H), 2.17 – 2.10 (br s, 1H), 1.98 – 1.96 (m, 1H) 1.82 – 1.68 (m, 6H), 1.20 – 1.19 (m, 1H), 1.09 – 1.07 (m, 1H) ppm

^{13}C NMR (126 MHz, d_6-DMSO): δ 173.4, 171.7, 168.6, 166.5, 152.8, 144.6, 140.7, 138.5, 133.7, 132.1, 131.8, 131.7, 130.7, 129.9, 129.5, 128.8, 128.5, 124.4, 124.1, 123.3, 120.1, 115.9, 109.7, 60.9, 56.2, 55.8, 52.7, 51.9, 51.5, 49.5, 47.5, 46.9, 45.2, 44.1, 41.5, 31.2, 31.7, 30.5, 29.6, 23.0 ppm

1.4.33 Synthesis of final CRBN PROTAC 29

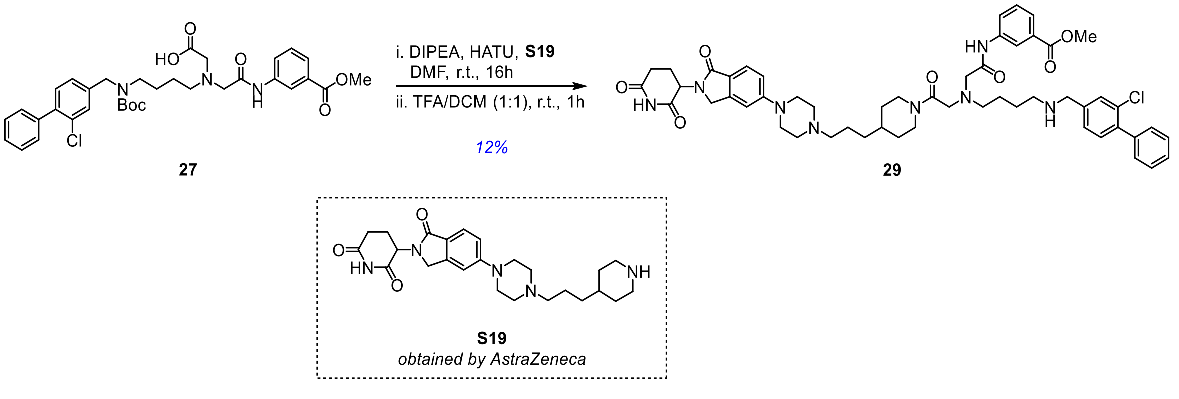

27 (20 mg, 31 µmol, 1.0 equiv) was dissolved in DMF (1 mL) and **S19** (14 mg, 31 µmol, 1.0 equiv) was added. To the stirring mixture, DIPEA (22 µL, 124 µmol, 4.0 equiv) was dropwise added, followed by HATU (13 mg, 34 µmol, 1.1 equiv). The reaction mixture was stirred for 16 hours at room temperature, after which LC–MS indicated an absence of starting material. The reaction mixture was concentrated in vacuo and redissolved in TFA/ CH_2Cl_2 1:1 (v/v, 1.5 mL). The mixture was stirred at room temperature for 1 hour, before being concentration in vacuo. The reaction was purified *via* by HPLC (method: 25–95% B, 20 min) to yield the title compound **29** as a white solid (4 mg, 3.7 µmol, 12%).

R_f : 0.13 (5% MeOH in CH_2Cl_2)

HRMS (ESI): $[M+H]^+$ calc. for $[C_{54}H_{63}O_7N_8Cl+H]^+$: 973.4743, found 973.4731

***v*$_{max}$** (neat)/cm^{-1}: 1686 (m, C=O), 1671, (m, C=O), 1619 (s, C=C), 1654 (s, C=C), 1578 (s, N-H or C-N), 1572 (s, N-H or C-N), 1561 (s, N-H or C-N), 1528 (s, N-H or C-N), 1491 (m, C=C), 1478 (m, C=C), 1450 (C-H), 1237 (w, C-O or C-N), 1200 (s, C-O or C-N), 1127 (m, C-O or C-N)

^{1}H NMR (700 MHz, d_6-DMSO): δ 8.33 (app. t, *J* = 7.6 Hz, 1H), 7.95 (s, 1H), 7.81 (d, *J* = 1.4 Hz, 1H), 7.74 (d, *J* = 1.5 Hz, 1H), 7.69 (d, *J* = 7.4 Hz, 1H), 7.59 (d, *J* = 8.5 Hz, 1H), 7.53 – 7.41 (m, 7H), 7.21 – 7.14 (m, 2H), 5.06 (dd, *J* = 13.3, 5.1 Hz, 1H), 4.35 (d, *J* = 17.0 Hz, 2H), 4.25 – 4.20 (m, 3H), 4.22 – 4.21 (d, *J* = 8.0 Hz, 1H), 3.85 (s, 3H), 3.79 – 3.49 (m, 8H), 3.24 – 2.90 (m, 11H), 1.98 – 1.95 (m, 2H), 2.38 (qd, *J* = 26.5, 13.3, 4.5 Hz, 1H), 1.98 – 1.95 (m, 1H) 1.74 – 1.49 (m, 9H), 1.23 – 1.22 (m, 2H), 1.08 – 1.22 (m, 1H), 0.96 – 0.98 (m, 1H) ppm

^{13}C NMR (126 MHz, d_6-DMSO): δ 173.4, 171.7, 168.6, 166.5, 152.8, 144.6, 140.7, 138.5, 133.7, 132.2, 131.8, 131.7, 130.7, 129.9, 129.6, 128.8, 128.5, 124.4, 124.1, 123.3, 120.1, 116.0, 108.0, 56.0, 52.7, 51.9, 50.9, 49.5, 47.5, 46.9, 45.6, 44.8, 42.2, 35.0, 32.9, 31.2, 31.7, 23.0, 23.0, 20.9 ppm

2. Biological and biophysical evaluation

2.1 Isothermal titration calorimetry (ITC)

All ITC experiments were performed at 25 °C using a MicroCal itc200 instrument (GE Healthcare). CK2α_K74A (10 mg/mL, 20mM Tris pH 8.0, 300 mM NaCl) was diluted in Tris buffer (200 mM Tris, 300 mM NaCl, 10% DMSO) and concentrated to 5–10 µM. Ligand **S13** or **24** in 100× stock solutions in DMSO was diluted into the buffer ensuring that the DMSO concentrations were carefully matched. For **S13** CK2α_K74A (5 µM) was loaded into the sample cell and 100 µM of the ligand was titrated in nineteen 2 µL injections of 2 s duration at 120 s intervals, with injector speed of 750 rpm. For **24** CK2α_K74A (7 µM) was loaded into the sample cell and 200 µM of the ligand was titrated into the cell. The solubility was limited above this concentration. Heats of dilution were determined in identical experiments, but without protein in the cell. The data fitting was performed with a single site binding model using the Origin software package.

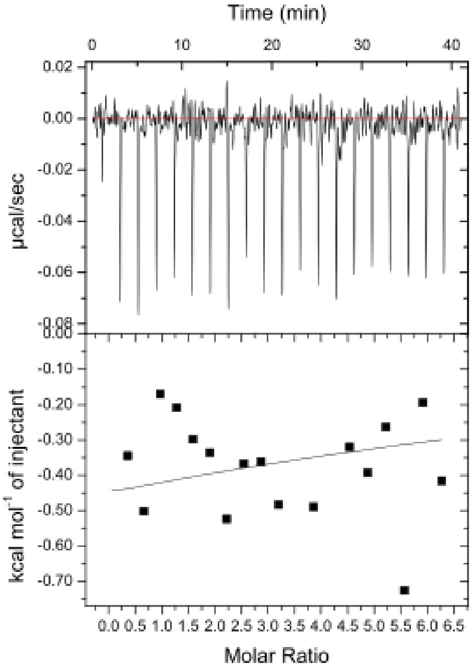

Figure S1: ITC of PROTAC **24**.

2.2 Cell lines

The HCT-116 and MDA-MB-231 cell lines were obtained from the American Type Culture Collection.

2.3 Cell culture

HCT-116 cells were cultured in McCoy's 5[a] (Modified) Medium (DMEM) (gibco, 16600082) supplemented with 10% fetal bovine serum (FBS) (gibco, 11875093) 2 mM L-glutamine and 50 µg/mL streptomycin. MDA-MB-231 cells were cultured in RPMI-1640 Medium supplemented with 10% FBS, 2 mM L-glutamine and 50 µg/ mL streptomycin. Cells were maintained for no more than 50 passages at 37 °C with 5% CO_2.

2.4 Treatment with PROTACs

Cells were counted using the Vi-CELL XR cell viability analyser, as per manufactures instructions, and the desired number of cells seeded in full media in individual 10 cm plates (Corning, #430167) and the cells were allowed to adhere overnight. The cells were then treated with the desired concentration of PROTAC. The PROTAC was dissolved in DMSO and was added directly to the cell culture media, the final DMSO concentration was 0.01%.

2.5 SDS-PAGE followed by Western blot

2.5.1 Lysate preparation

Upon removal from the incubator, the plates containing the cells were placed on ice. The media was aspirated and the plates washed with twice with ice cold PBS. RIPA buffer containing protease and phosphatase inhibitor was added and the cells were detached from the surface with a cell scraper. After centrifugation (8000 rpm for 3 min at 4 °C), the supernatant was discarded and the pellet flash frozen and stored at −80 °C or taken forward for lysis. The pellet was resuspended in RIPA buffer containing protease and phosphatase inhibitor and incubated at 4 °C for 10 minutes with rotation. The sample was sonicated for 3 min (30 seconds on, 30 seconds off) and subjected to centrifugation (21,000*g* for 10 min at 4 °C). The supernatant was transferred and the protein concentration quantified by the Direct Detect Spectrometer as per manufacturer's instructions.

2.5.2 SDS-PAGE

30 µg of protein was denatured by heating at 80 °C for 10 min in NuPAGE LDS sample buffer (4X), NuPAGE Sample Reducing Agent (10x) and made up to 25 µL with RIPA buffer (multiplied 181 up as required). The samples were loaded onto a 4–12% Bis-Tris 1.0 mm pre-cast polyacrylamide gel (12 µL loaded onto 12 well, 10 µL loaded onto 15 well) with a molecular weight ladder. With the MOPS running buffer, voltage was applied with at 60 V for 30 minutes followed by 120 V. The voltage was stopped once the dye front reached the bottom of the gel (≈90 minutes).

2.5.3 Western blot preparation

The proteins were transferred from the gel to a PVDF membrane using an iBlot2 gel transfer device using the pre-programmed P0 method. Following transfer, the membrane was washed sequentially in methanol, H_2O and TBS-T (0.1% Tween-20 in TBS). The membrane was blocked with 5% milk in TBS for 1 h at room temperature and then incubated with primary antibody in 1% milk in TBS-T overnight at

4 °C. The membrane was then washed three times for 5 min with TBS-T and incubated with secondary antibody in 1% milk in TBS-T for 1 hr at room temperature. The membrane was washed three times for 5 min with TBS-T washed once with TBS for 5 minutes and then imaged using the LiCor CLx or LiCor Odyssey. Protein quantification was performed by densitometry using Image Studio analysis software (LI-COR Biosciences).

2.5.4 Western blot results

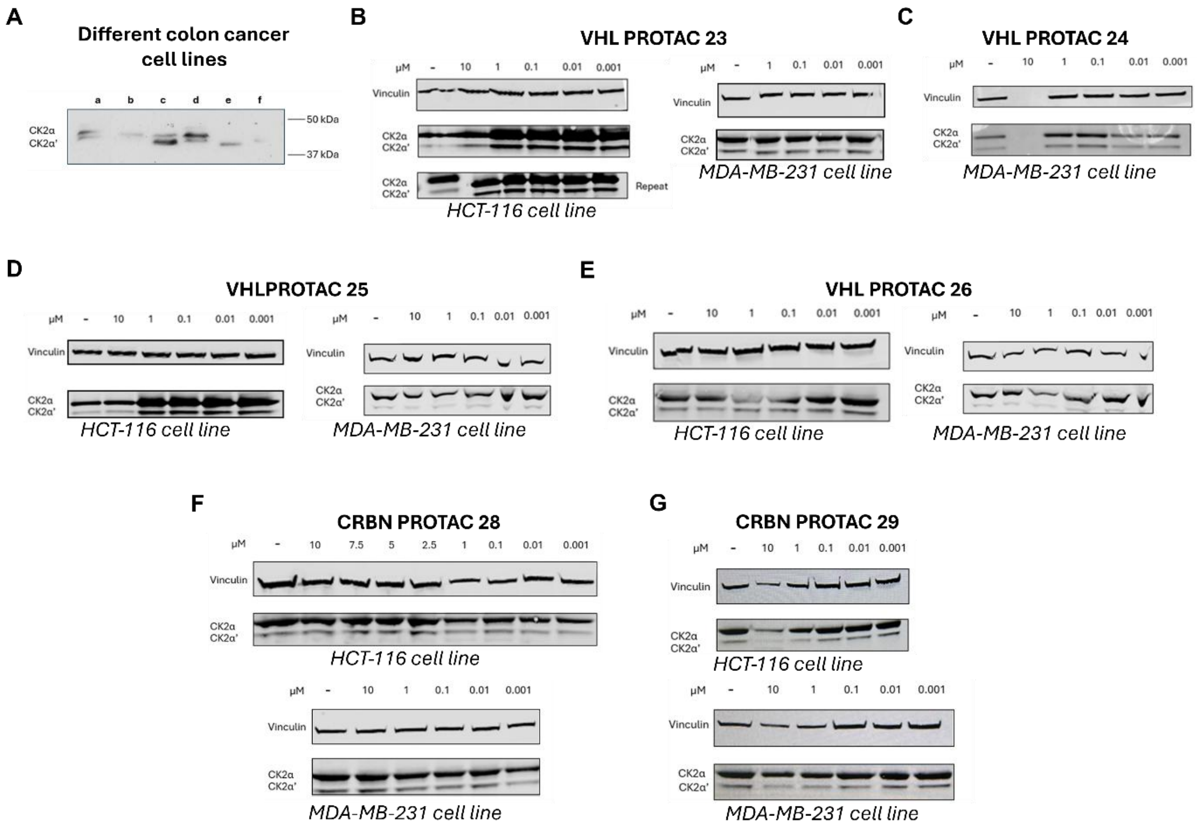

Figure S2: Results of Western blot assays carried out with the PROTACs to determine their ability to degrade CK2α. Panel **A** shows the Western blot analysis on six different colon cancer cell lines, which are as follows: **a**: LoVo; **b**: HT29; **c**: RKO; **d**: HCT-116; **e**: DLD-1; **f**: SW-480; Panels **B** – **G** show the Western blot analysis of the HCT-116 and MDA-MB-231 cell lines treated with the VHL and CRBN PROTACs. The cells were treated for 24h in a concentration dependent assay. 0.1% DMSO was used as a control.

2.5.5 Uncropped Western blots

- ***Different colon cancer cell lines***

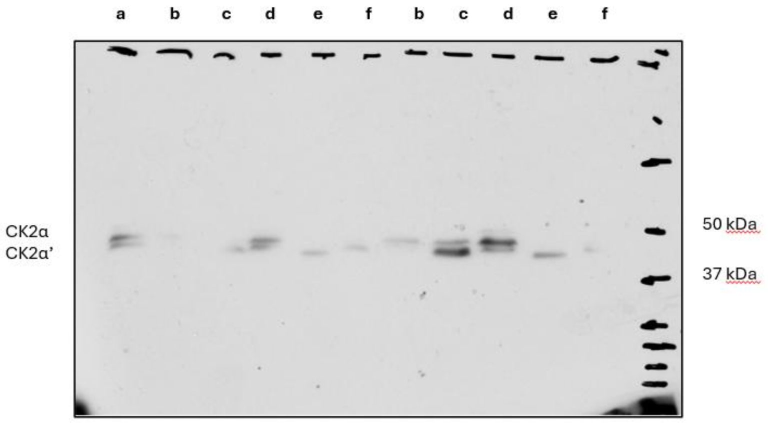

- ***VHL PROTAC 23 with HCT-116 cell line***

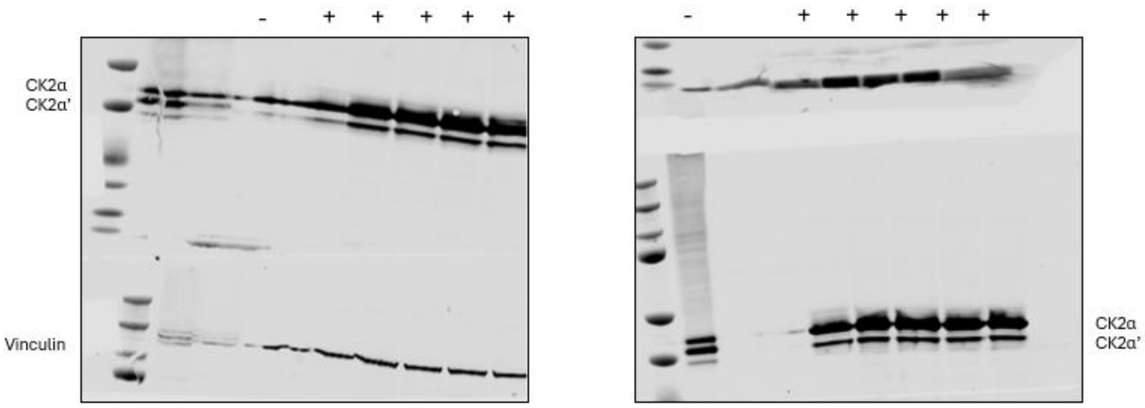

- ***VHL PROTAC 25 with HCT-116 cell line***

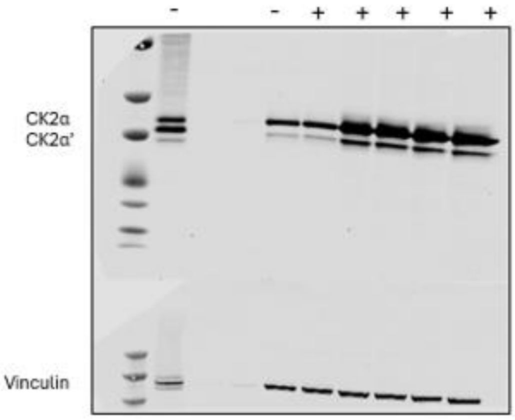

- ***VHL PROTAC 26 with HCT-116 cell line***

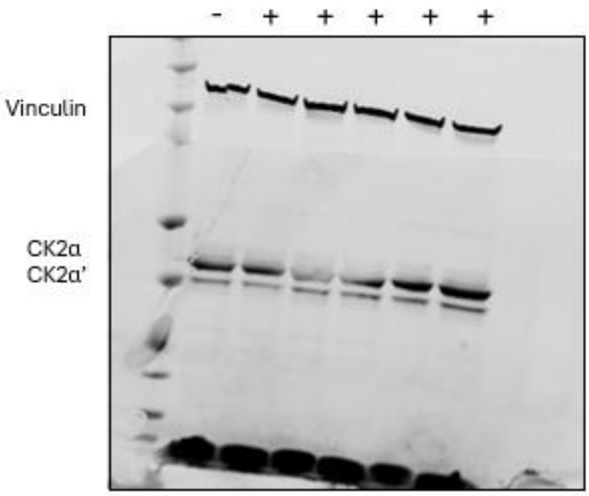

- ***CRBN PROTAC 28 with HCT-116 cell line***

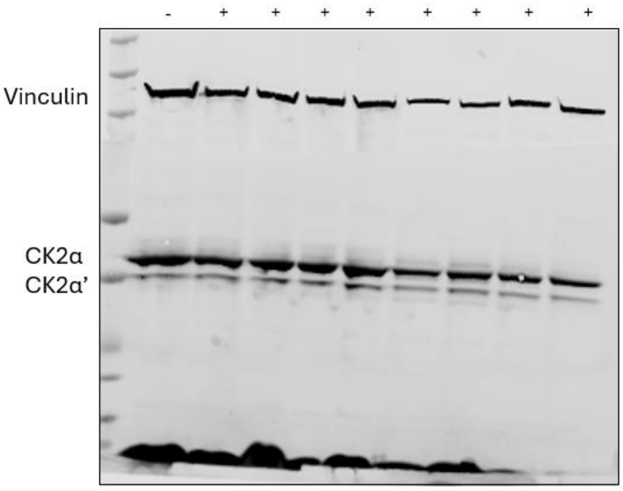

- ***CRBN PROTAC 29 with HCT-116 cell line***

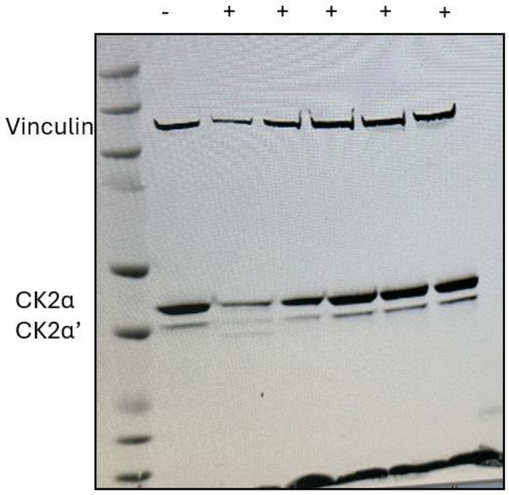

- ***VHL PROTAC 23 and CRBN PROTAC 28 in MDA-MB-231 cell line***

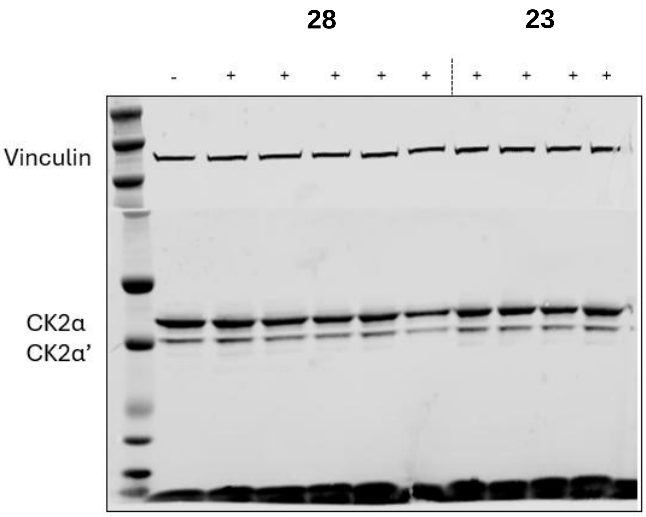

- ***VHL PROTAC 24 with MDA-MB-23 cell line***

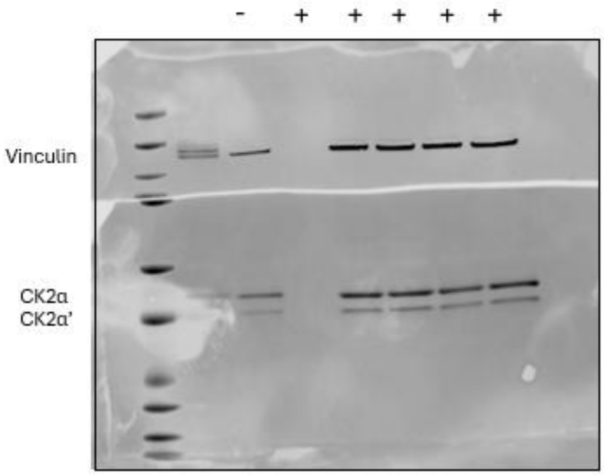

- ***VHL PROTACs 25 and 26 with MDA-MB-23 cell line***

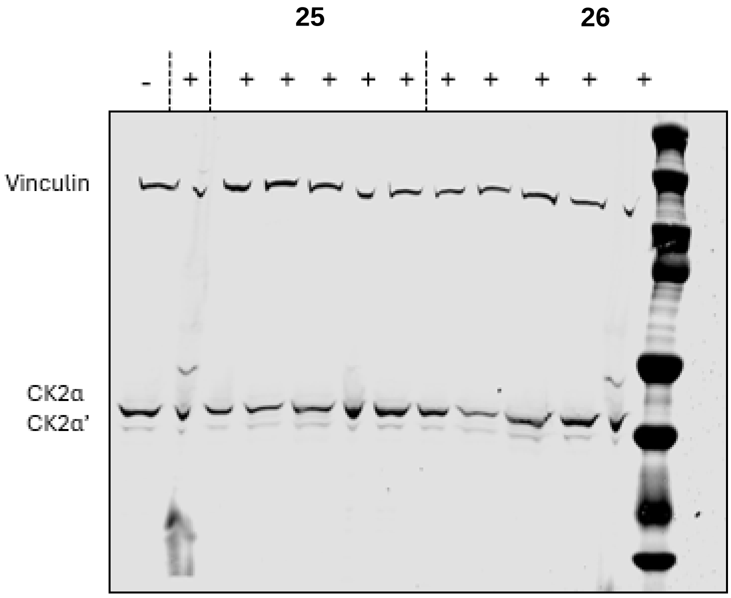

- ***CRBN PROTAC 29 with MDA-MB-23 cell line***

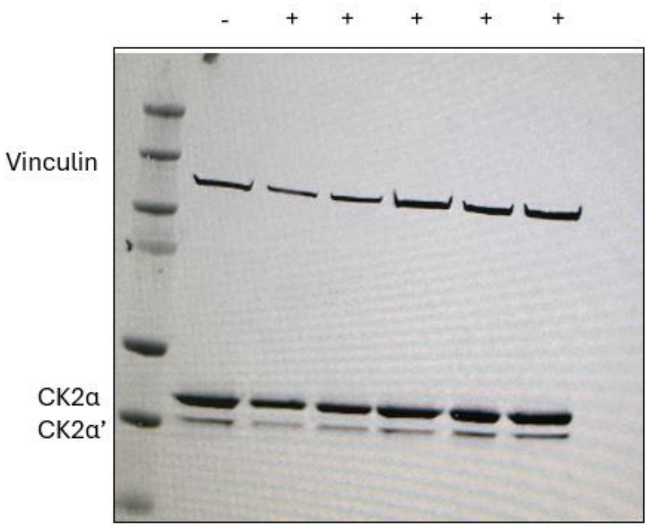

2.6 Sources of antibodies and reagents used

Antibodies	Source	Catalogue#	Dilution
Rabbit anti-CK2α/α’	Cell Signaling	2656	1:1000
Rabbit anti-Vinculin	Cell Signaling	E1E9V	1:1000
IR Dye 800CW donkey anti-rabbit	LiCor Bio	926-32213	1:5000
IR Dye 680RD goat anti-rabbit	LiCor Bio	926-68071	1:5000
**Reagents**	**Source**	**Catalogue#**	**Dilution**
RIPA-Buffer (Pierce)	Thermo Scientific	89900	
Protease Inhibitor (Pierce complete EDTA-free protease inhibitor tablet)	Roche	11873580001	100x
Phosphatase Inhibitor (HALT Phosphatase inhibitor cocktail)	Thermo Scientific	78427	100x
NuPAGE LDS sample buffer	Invitrogen	NP0007	4x
NuPAGE sample reducing agent	Invitrogen	11569166	10x
Molecular weight ladder (Precision plus protein dual colour standard)	Bio-Rad	1610374	

4-12% Bis-Tris 1.0 mm gel (12 well)	NuPAGE	NP0322	
4-12% Bis-Tris 1.0 mm gel (15 well)	NuPAGE	NP0323	
MOPS running buffer	NuPAGE	NP0001	20x
iBlot 2 gel transfer device	Invitrogen	NP0004	
PVDF membrane (iBlot 2 transfer stacks, PVDF)	Invitrogen	IB24001	
Tris-buffered saline tablets	Sigma-Aldrich	12161700	
Tween-20 (10% Tween 20)	Bio-Rad	1610781	100x
Milk (skimmed milk powder)	Marvel Original		

3. References

(1) Yilmaz, E.; Bier, D.; Guillory, X.; Briels, J.; Ruiz-Blanco, Y. B.; Sanchez-Garcia, E.; Ottmann, C.; Kaiser, M. *Chem. Eur. J.* **2018**, *24*, 13807–13814. doi:10.1002/chem.201801074

(2) Budhadev, D.; Poole, E.; Nehlmeier, I.; Liu, Y.; Hooper, J.; Kalverda, E.; Akshath, U. S.; Hondow, N.; Turnbull, W. B.; Pöhlmann, S.; Guo, Y.; Zhou, D. *J. Am. Chem. Soc.* **2020**, *142*, 18022–18034. doi:10.1021/jacs.0c06793

(3) Anabuki, T.; Tsukahara, M.; Matsuura, H.; Takahashi, K. *Biosci. Biotechnol. Biochem.* **2016**, *80*, 432–439. doi:10.1080/09168451.2015.1104240

(4) Pokorski, J. K.; Breitenkamp, K.; Liepold, L. O.; Qazi, S.; Finn, M. G. *J. Am. Chem. Soc.* **2011**, *133*, 9242–9245. doi:10.1021/ja203286n

(5) Yang, C.; Edsall, R.; Harris, H. A.; Zhang, X.; Manas, E. S.; Mewshaw, R. E. *Bioorg. Med. Chem.* **2004**, *12*, 2553–2570. doi:10.1016/j.bmc.2004.03.028

(6) De Fusco, C.; Brear, P.; Iegre, J.; Georgiou, K. H.; Sore, H. F.; Hyvönen, M.; Spring, D. R. *Bioorg. Med. Chem.* **2017**, *25*, 3471–3482. doi:10.1016/j.bmc.2017.04.037

(7) Steinebach, C.; Voell, S. A.; Vu, L. P.; Bricelj, A.; Sosič, I.; Schnakenburg, G.; Gütschow, M. *Synthesis* **2020**, *52*, 2521–2527. doi:10.1055/s-0040-1707400

4. Copies of NMR spectra

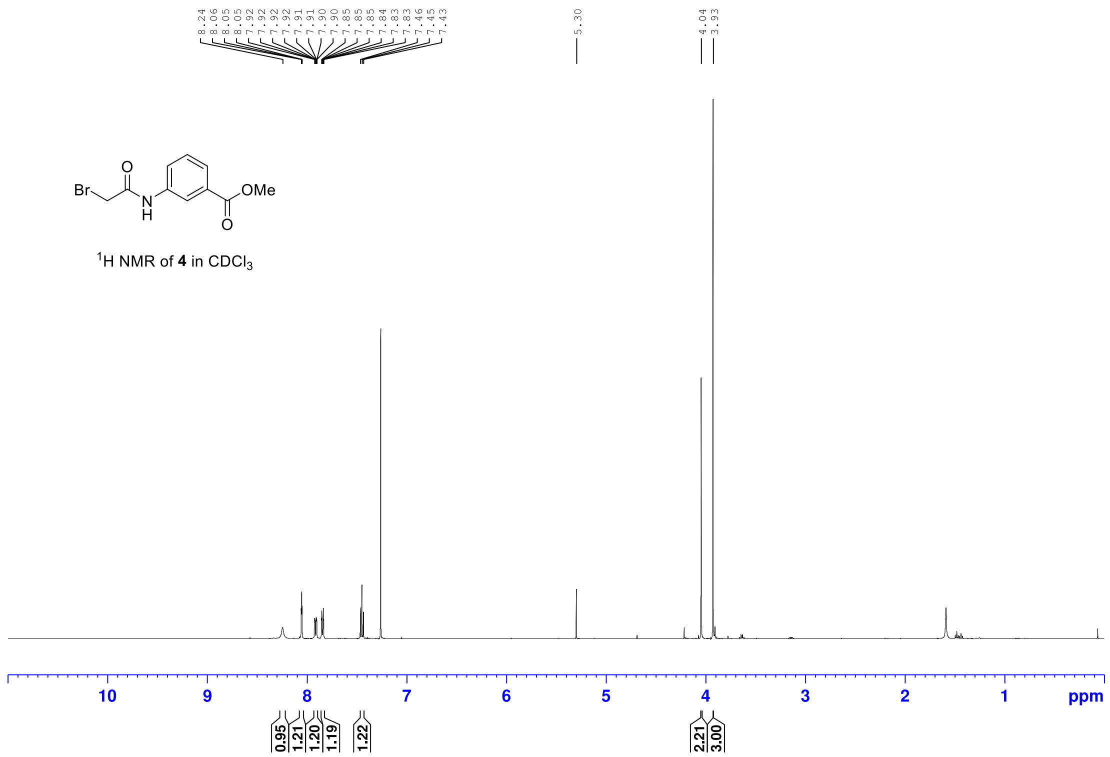

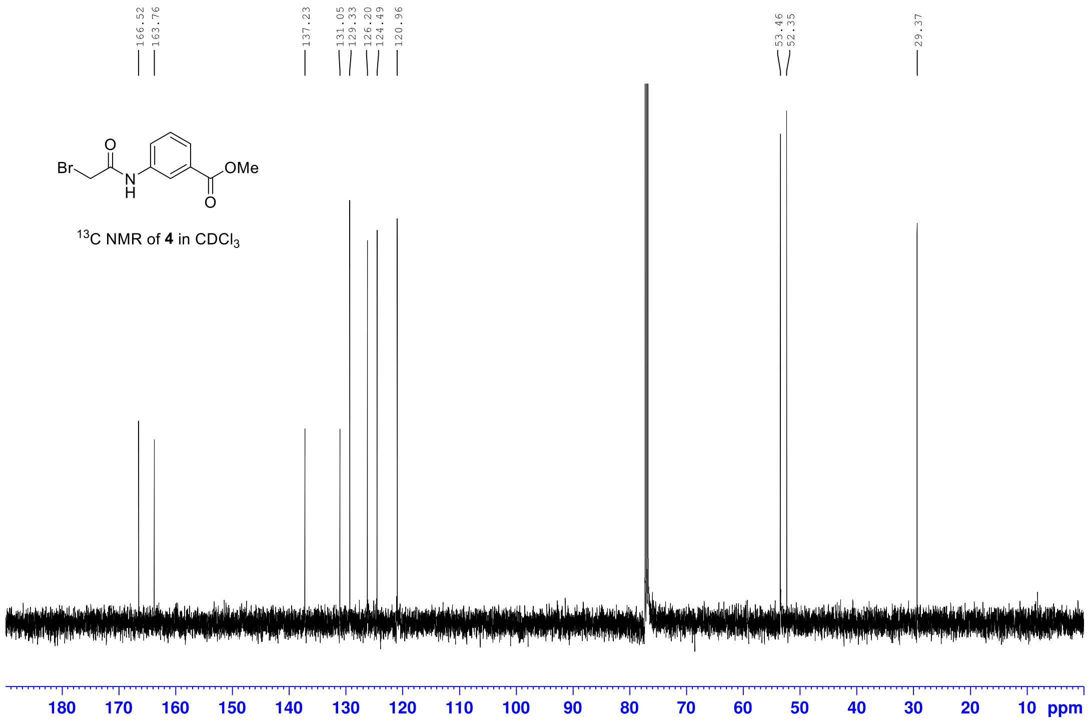

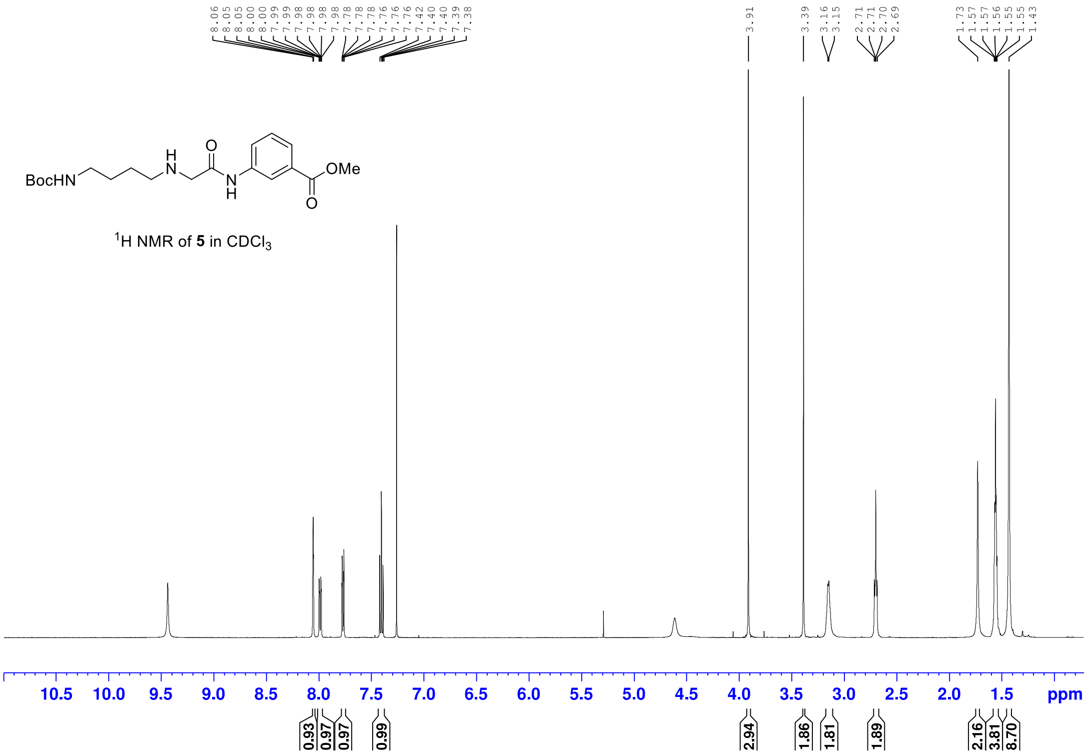

^{1}H NMR of **5** in $CDCl_3$

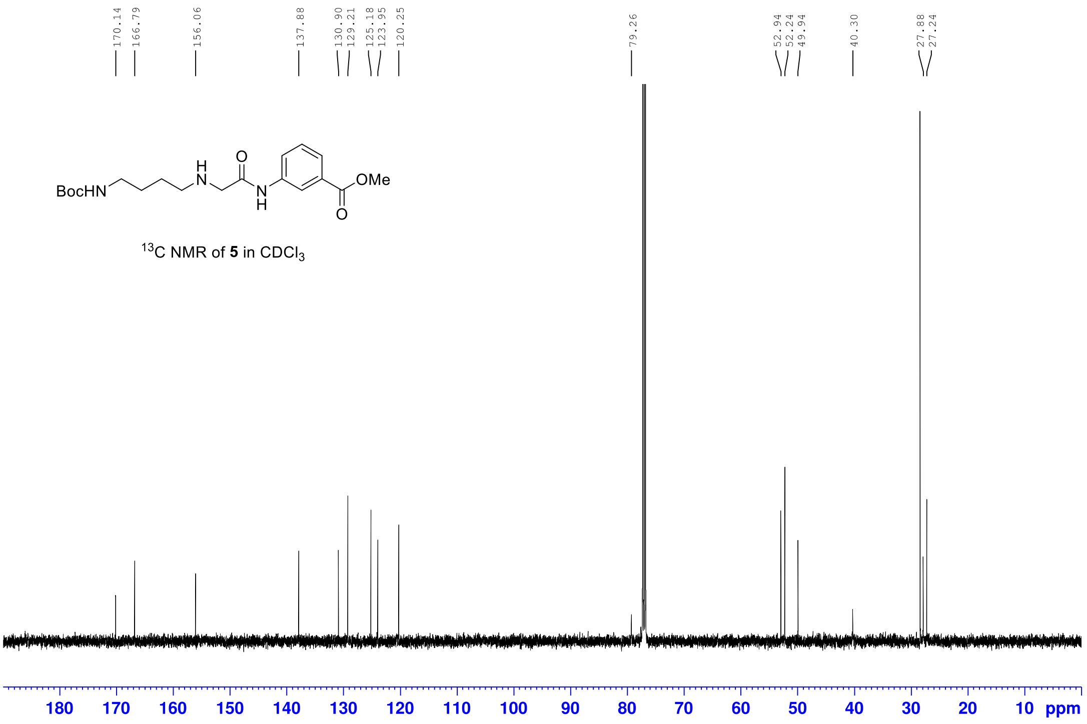

^{13}C NMR of **5** in $CDCl_3$

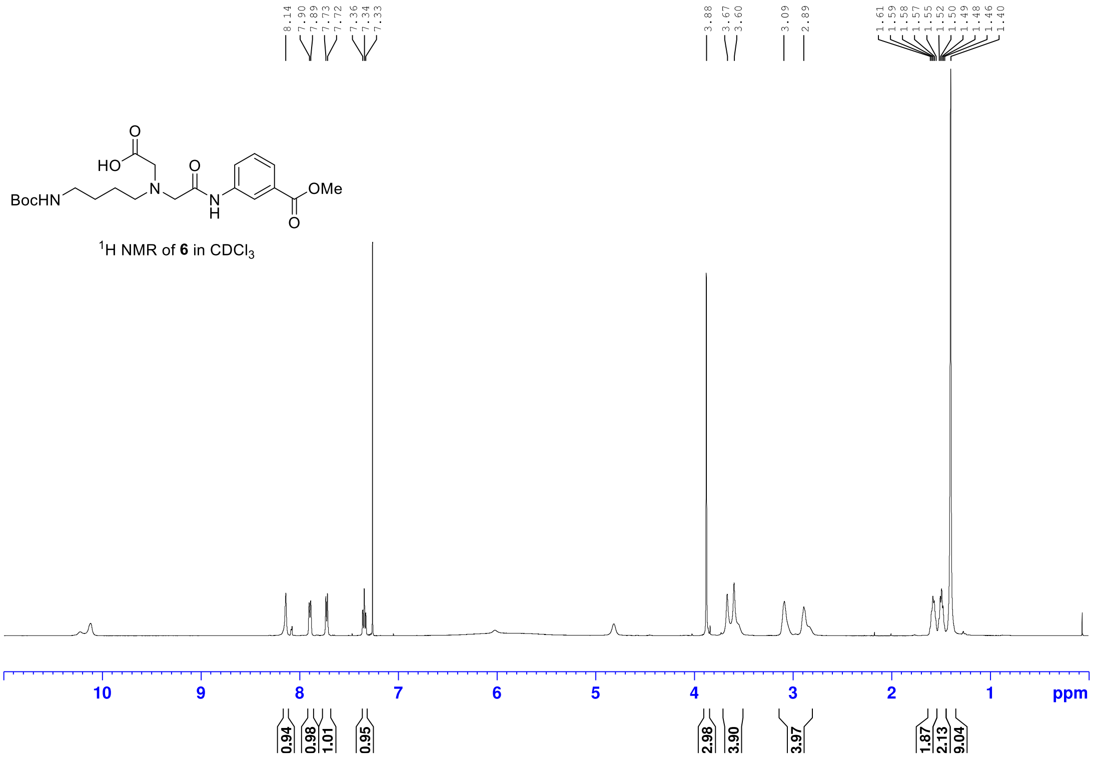
8.14 7.90 7.89 7.73 7.72 7.36 7.34 7.33
3.88 3.67 3.60
3.09 2.89
1.61 1.59 1.58 1.57 1.55 1.52 1.50 1.49 1.48 1.46 1.40
HO
O
O
BocHN
N
N
H
OMe
O
^{1}H NMR of **6** in $CDCl_3$
10 9 8 7 6 5 4 3 2 1 ppm
0.94 0.98 1.01 0.95 2.98 3.90 3.97 1.87 2.13 9.04

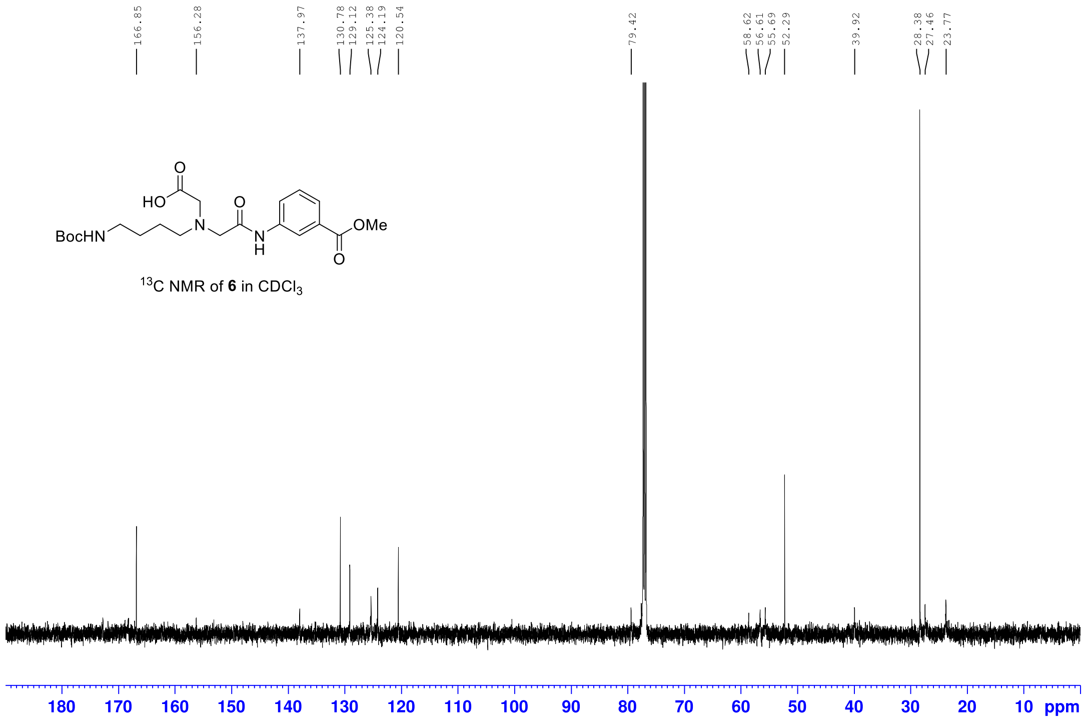
166.85 156.28 137.97 130.78 129.12 125.38 124.19 120.54 79.42 58.62 56.61 55.69 52.29 39.92 28.38 27.46 23.77
HO
O
O
BocHN
N
N
H
OMe
O
^{13}C NMR of **6** in $CDCl_3$
180 170 160 150 140 130 120 110 100 90 80 70 60 50 40 30 20 10 ppm

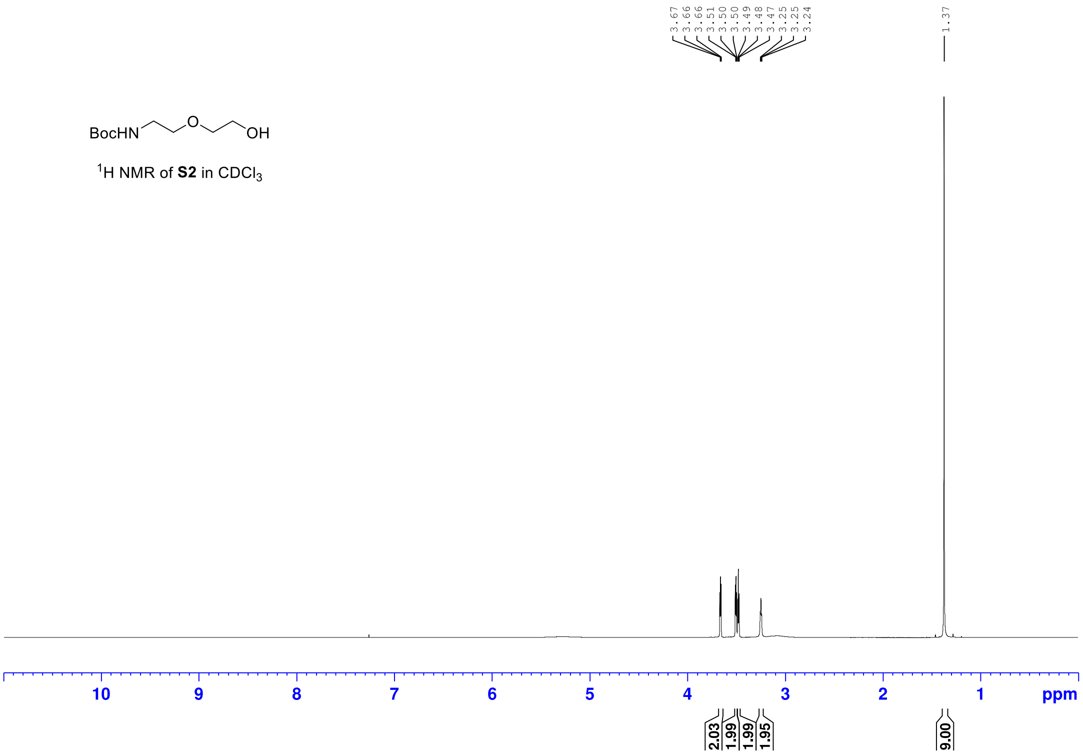

^{1}H NMR of **S2** in $CDCl_3$

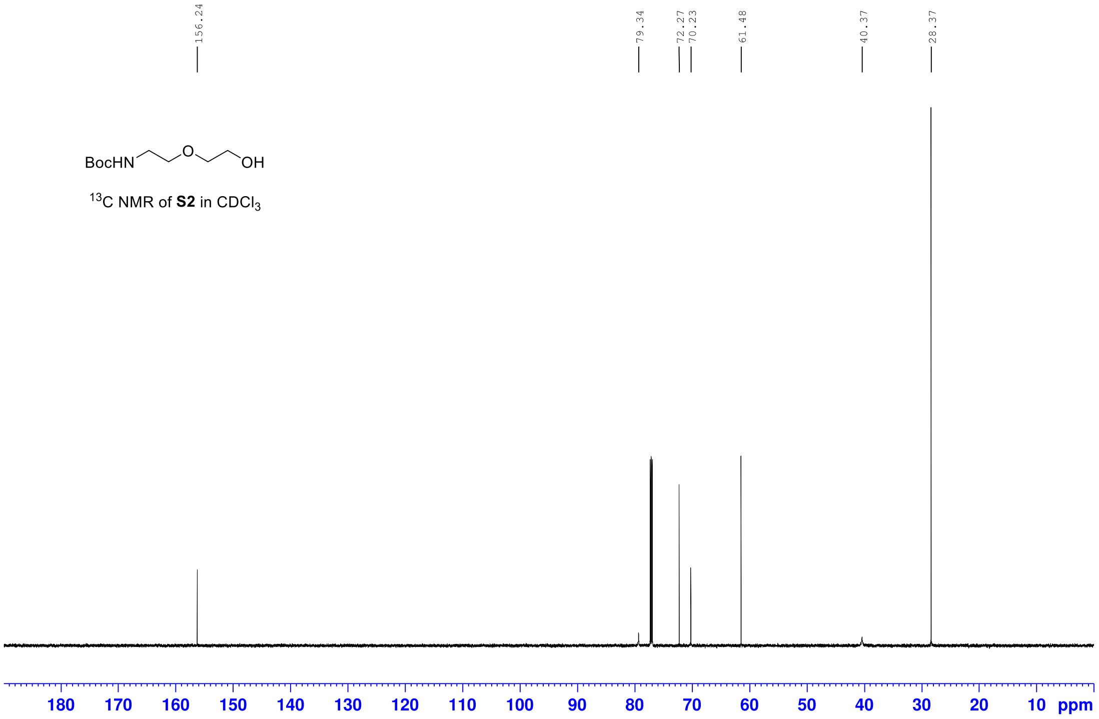

^{13}C NMR of **S2** in $CDCl_3$

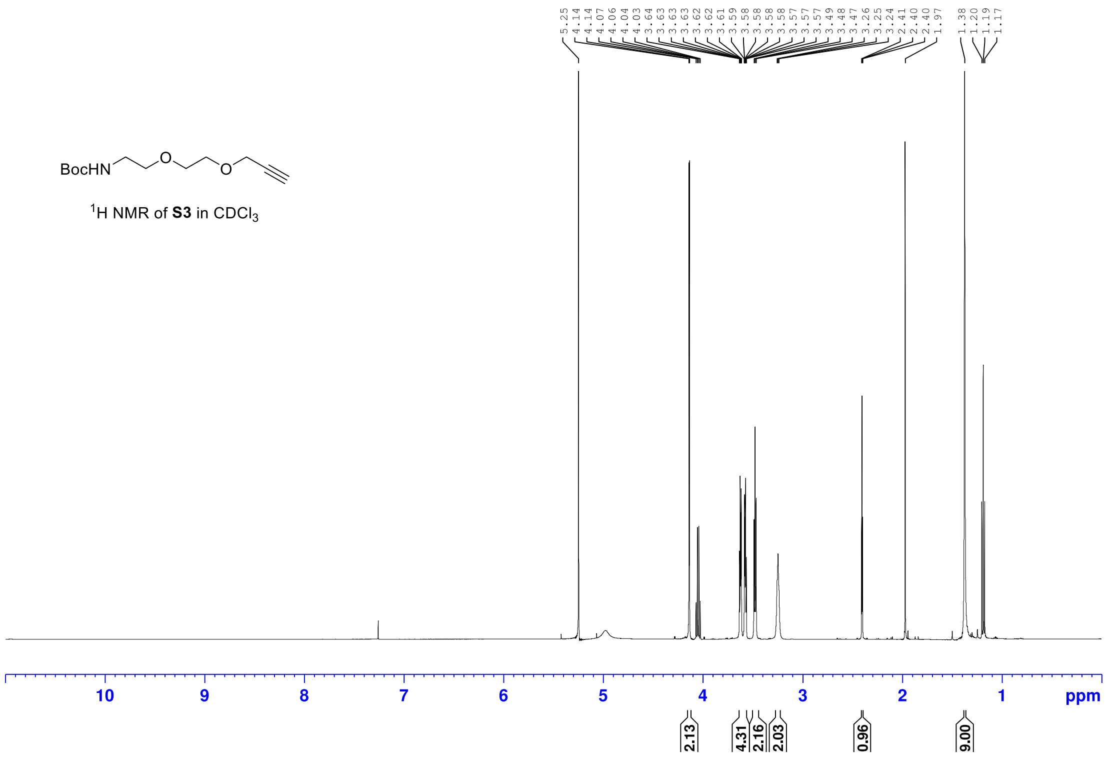

^{1}H NMR of **S3** in $CDCl_3$

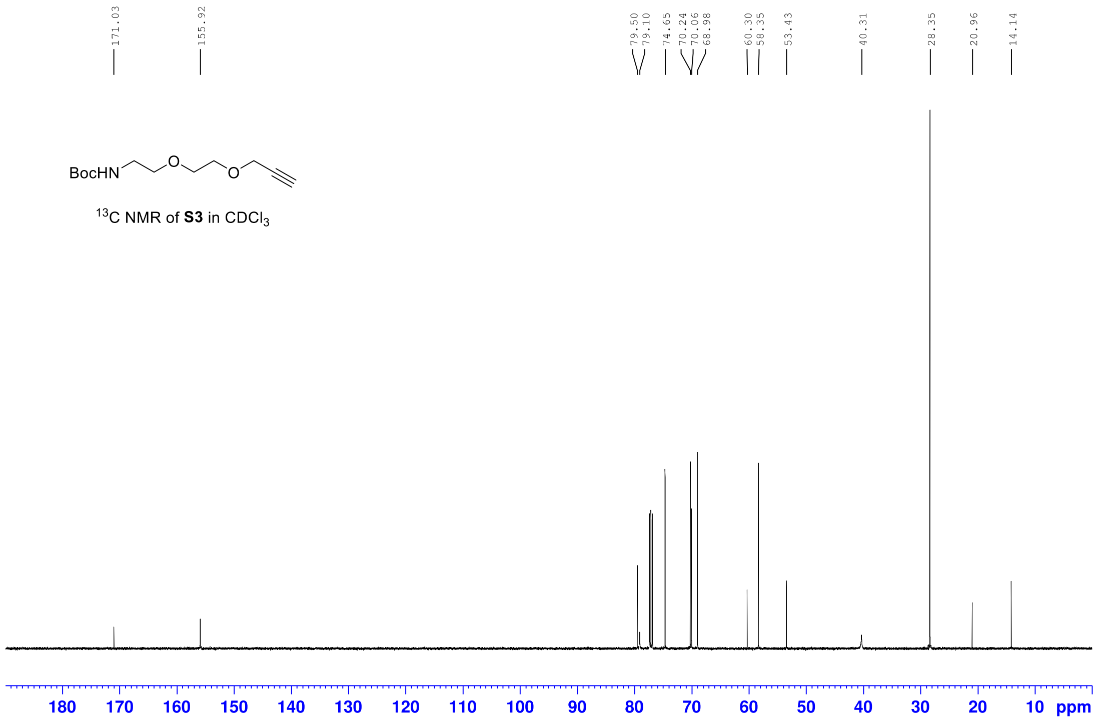

^{13}C NMR of **S3** in $CDCl_3$

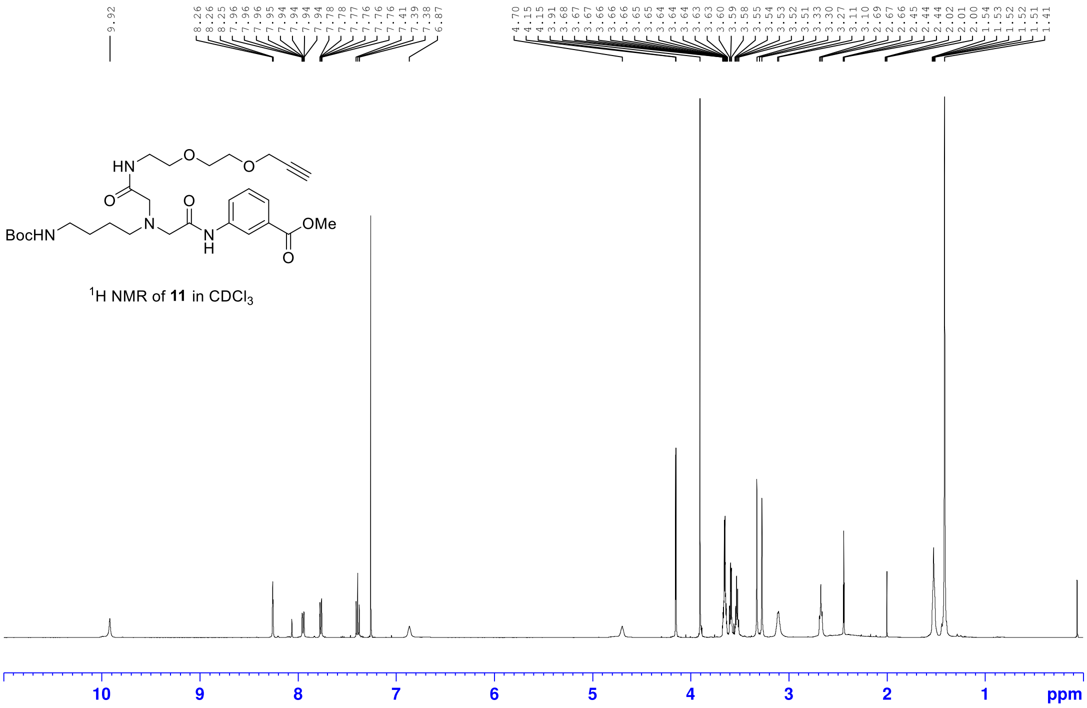

^{1}H NMR of **11** in $CDCl_3$

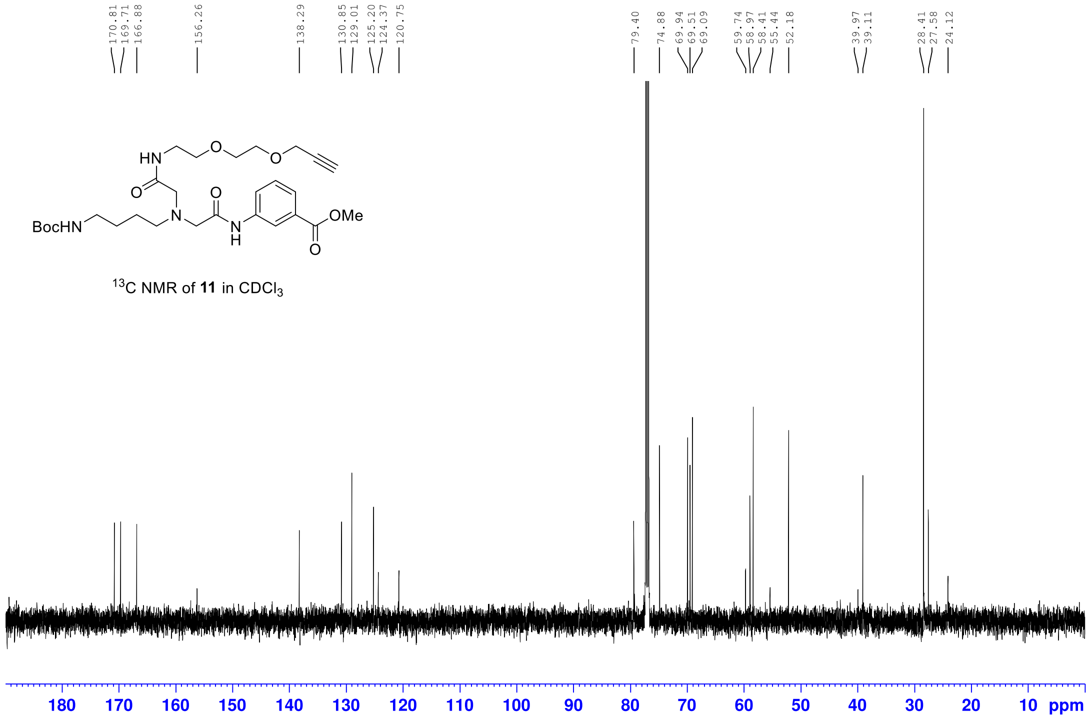

^{13}C NMR of **11** in $CDCl_3$

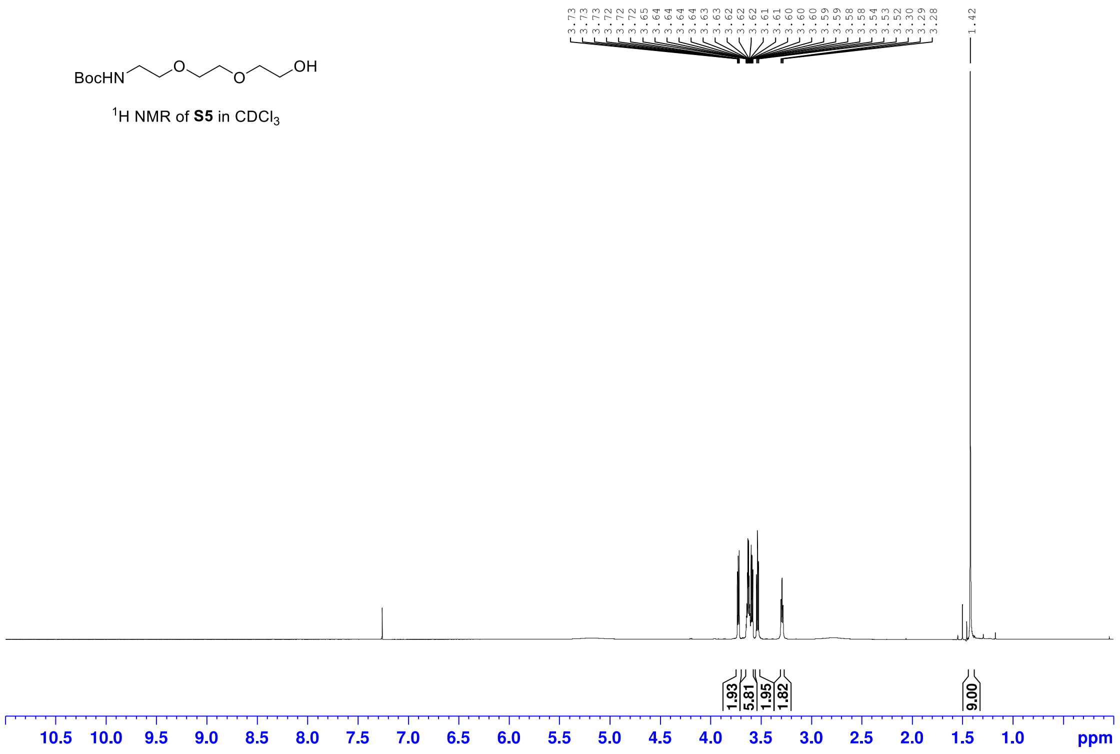
BocHN
O
O
OH
^{1}H NMR of **S5** in $CDCl_3$
1.42
1.93
5.81
1.95
1.82
9.00
ppm

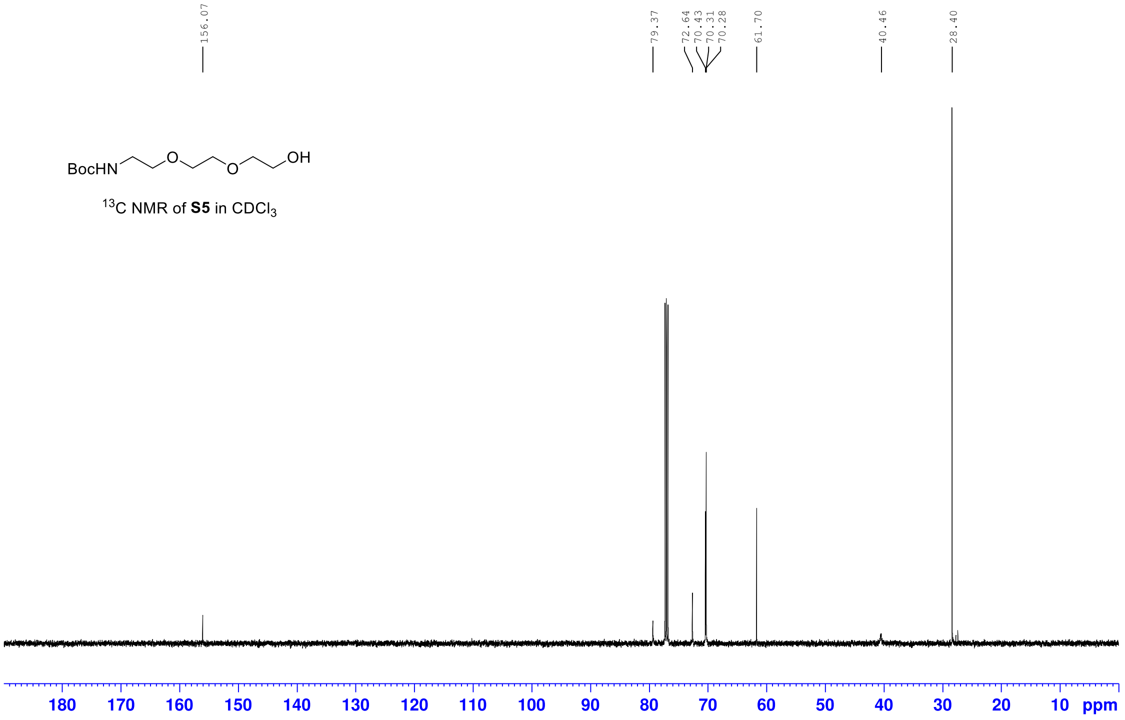
156.07
79.37
72.64
70.43
70.31
70.28
61.70
40.46
28.40
BocHN
O
O
OH
^{13}C NMR of **S5** in $CDCl_3$
ppm

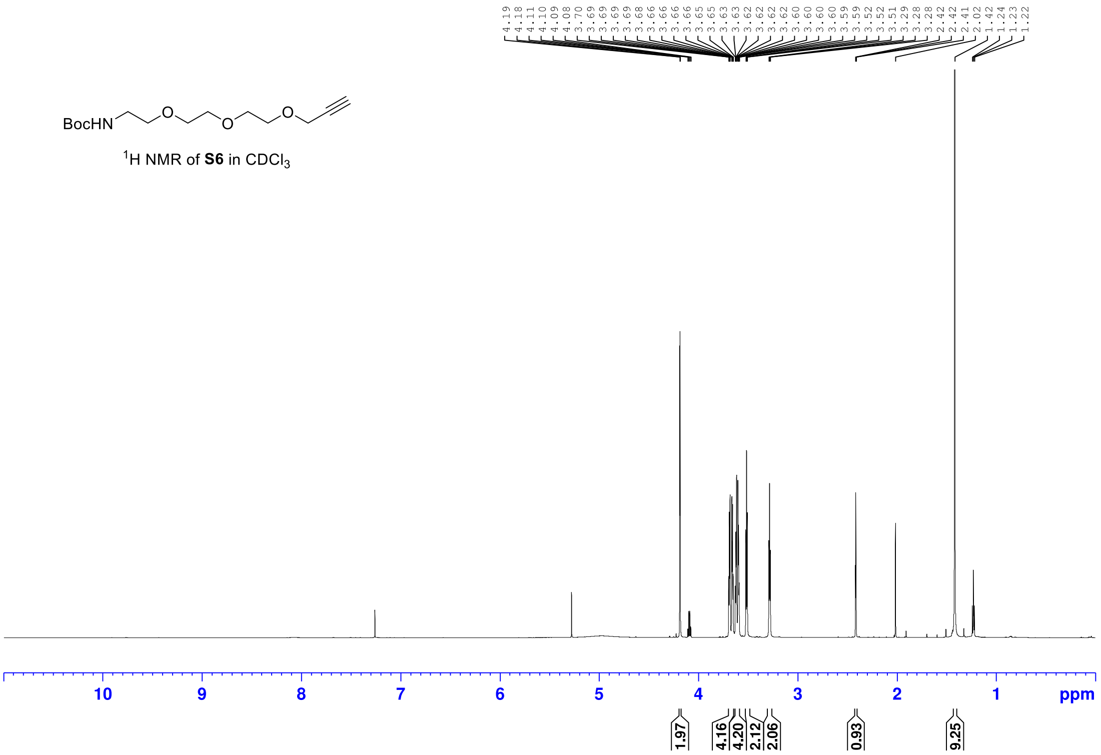

^{1}H NMR of **S6** in $CDCl_3$

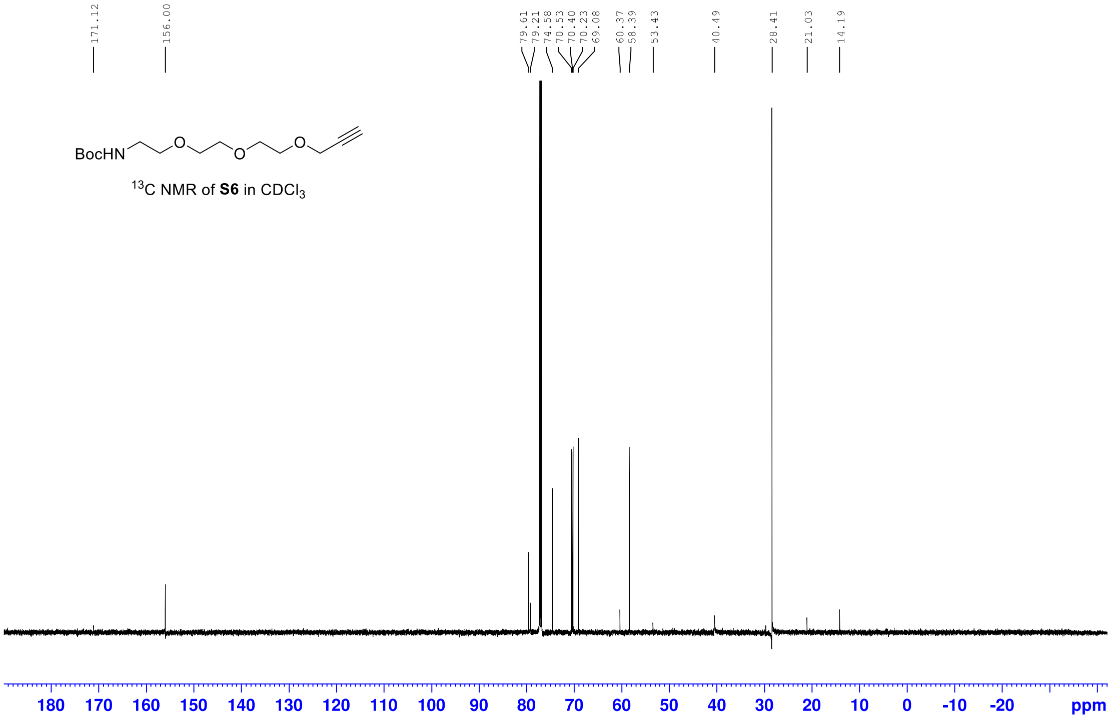

^{13}C NMR of **S6** in $CDCl_3$

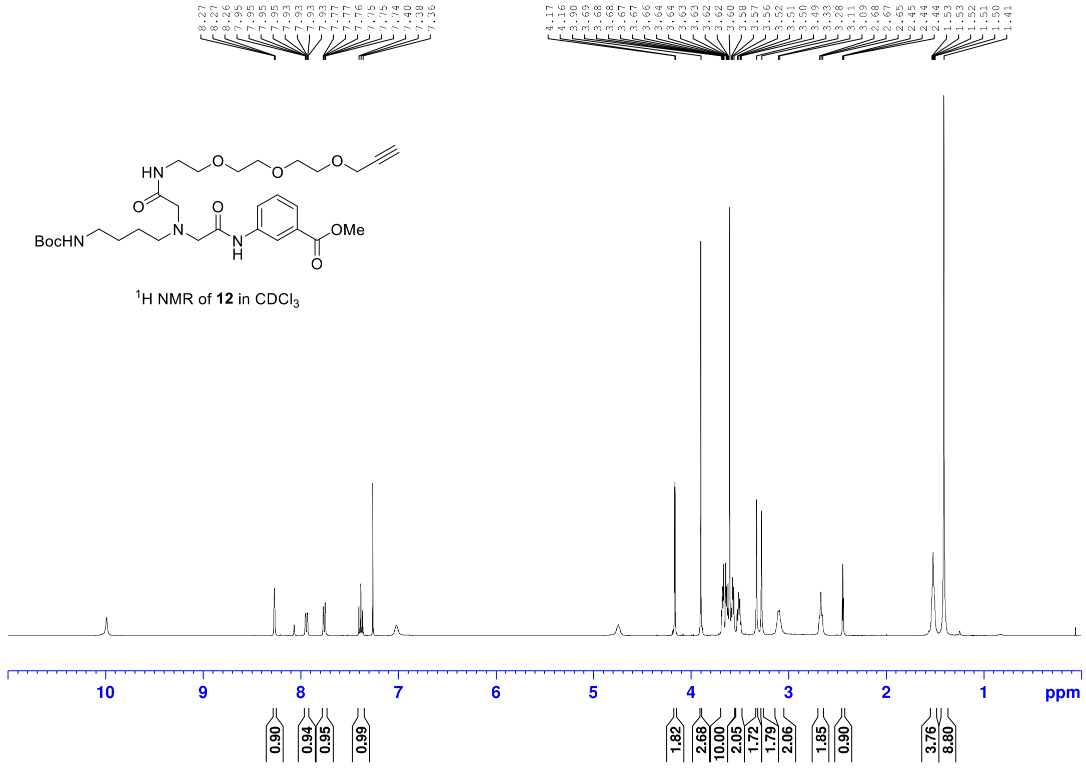

^{1}H NMR of **12** in $CDCl_3$

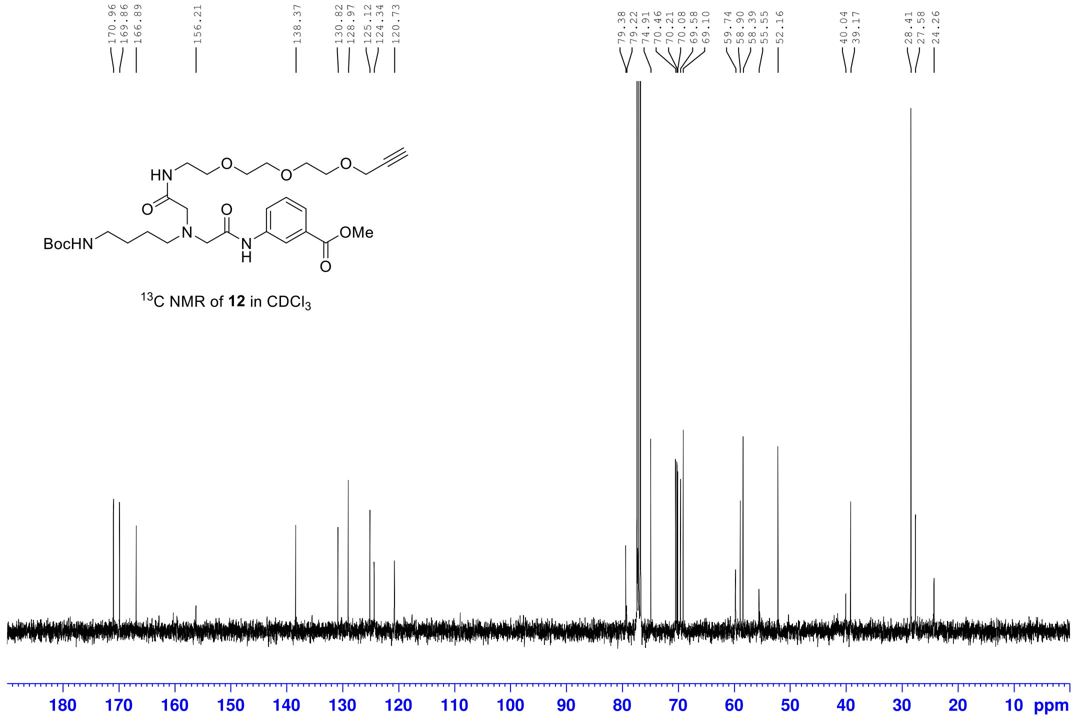

^{13}C NMR of **12** in $CDCl_3$

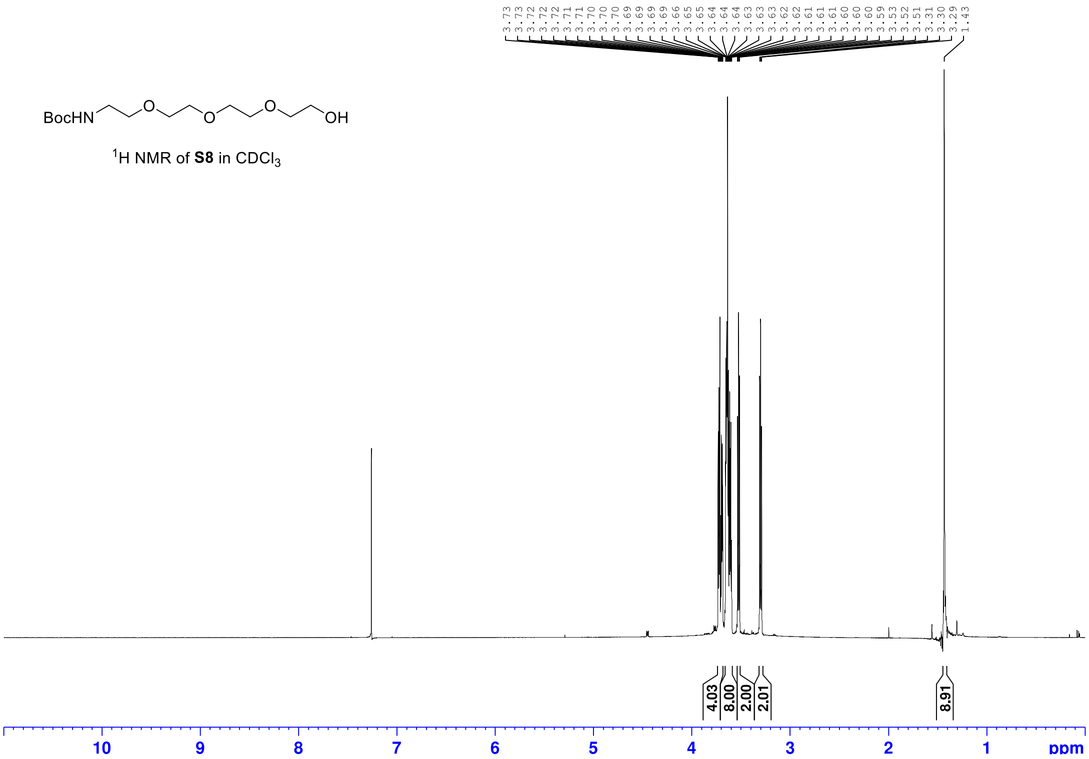

^{1}H NMR of **S8** in $CDCl_3$

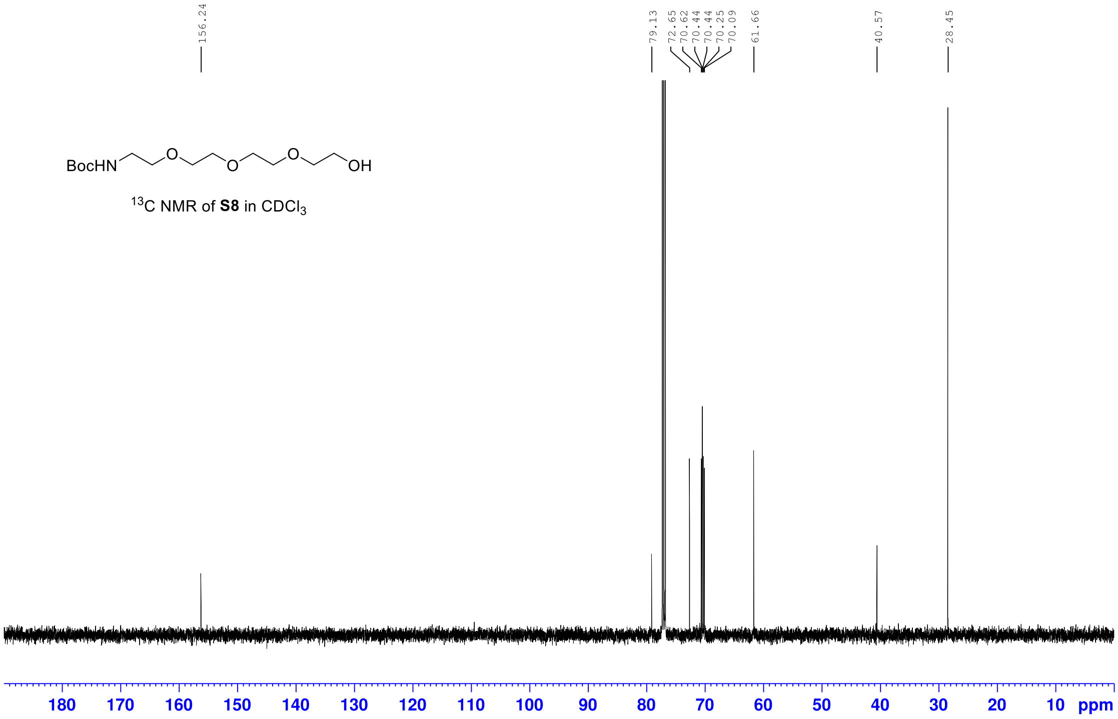

^{13}C NMR of **S8** in $CDCl_3$

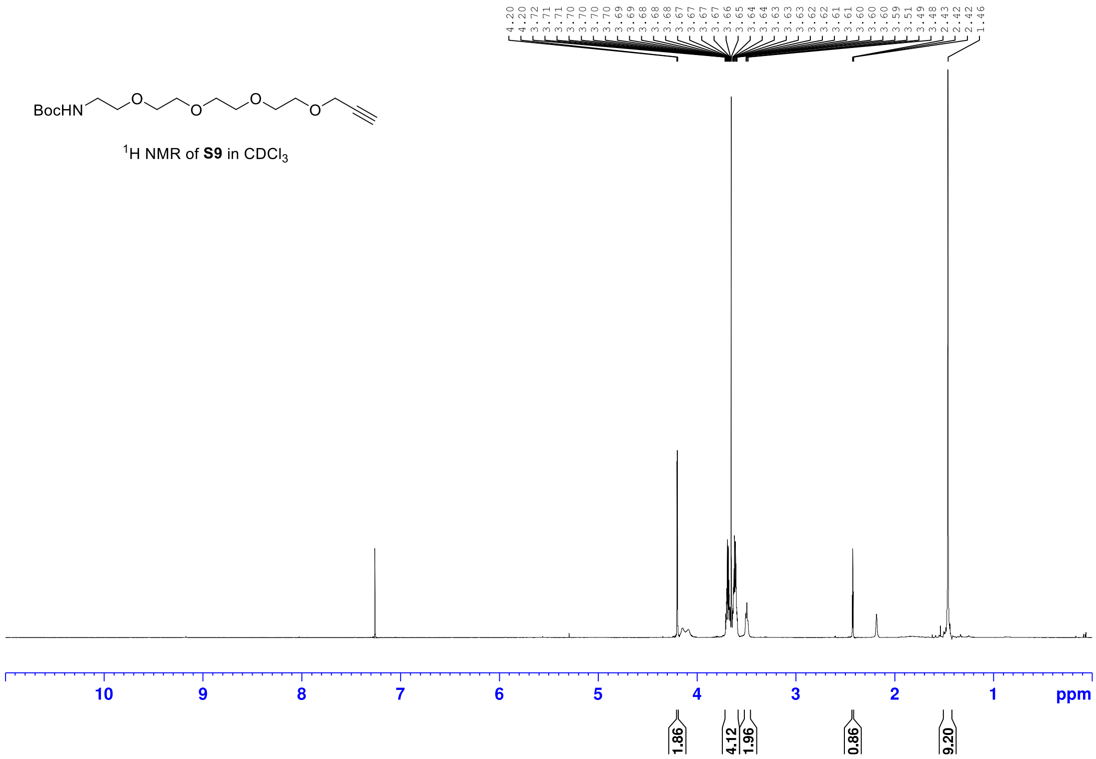

^{1}H NMR of **S9** in $CDCl_3$

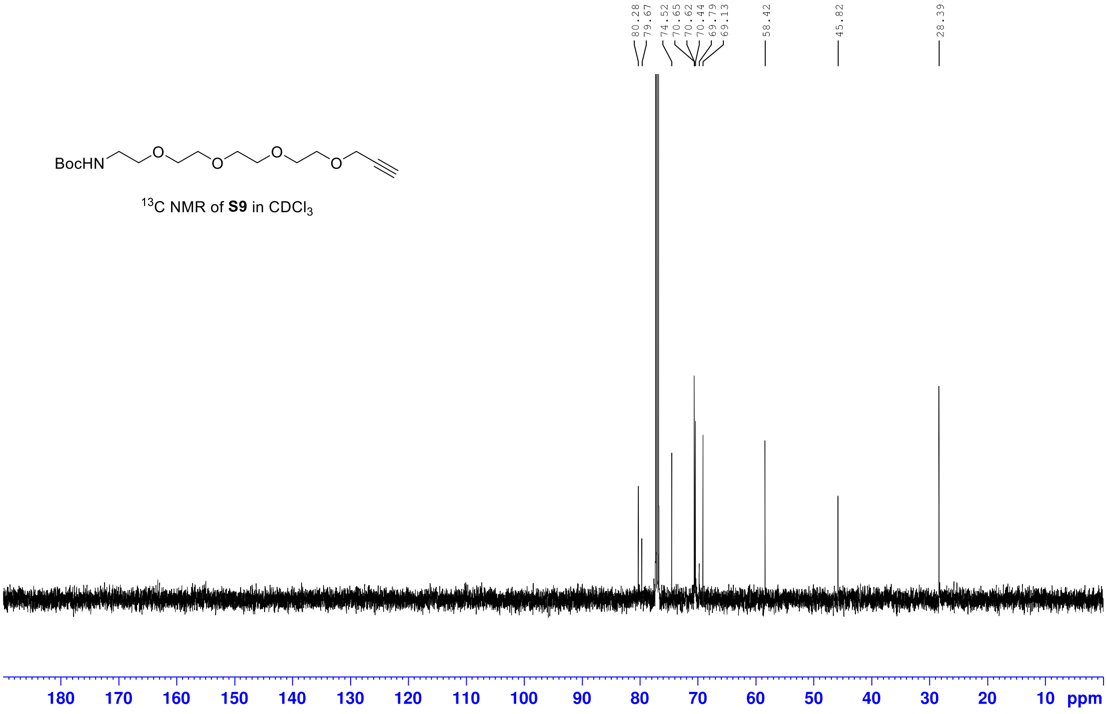

^{13}C NMR of **S9** in $CDCl_3$

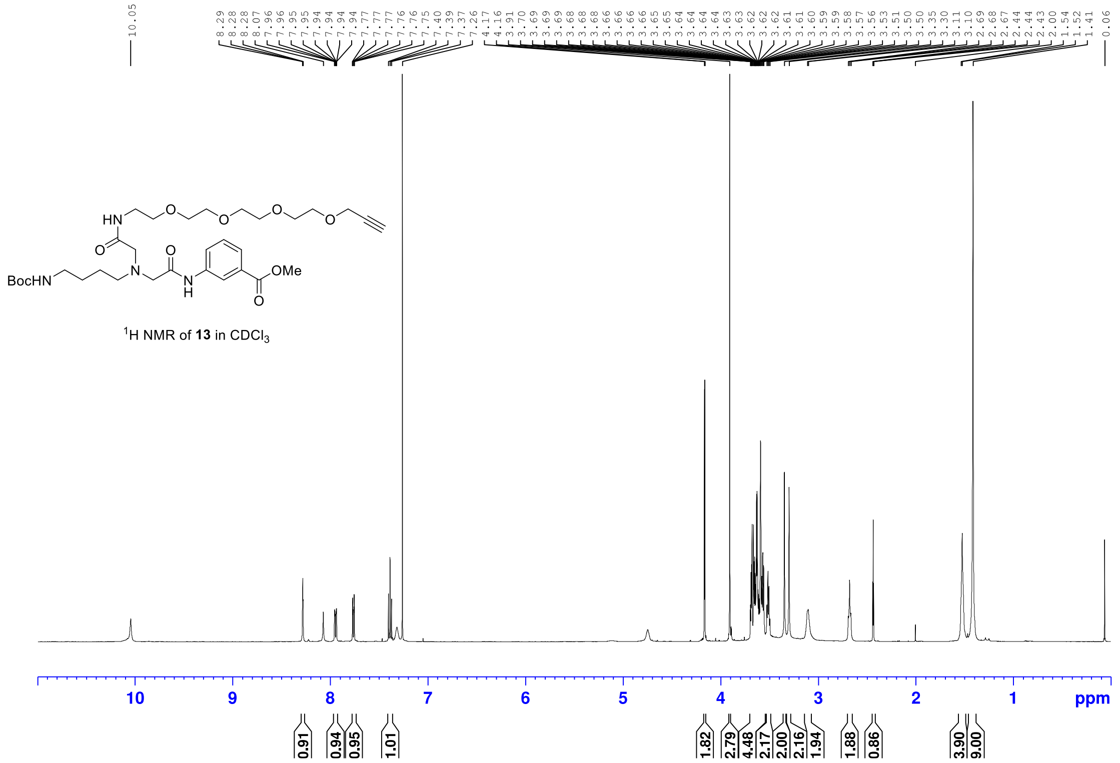

^{1}H NMR of **13** in $CDCl_3$

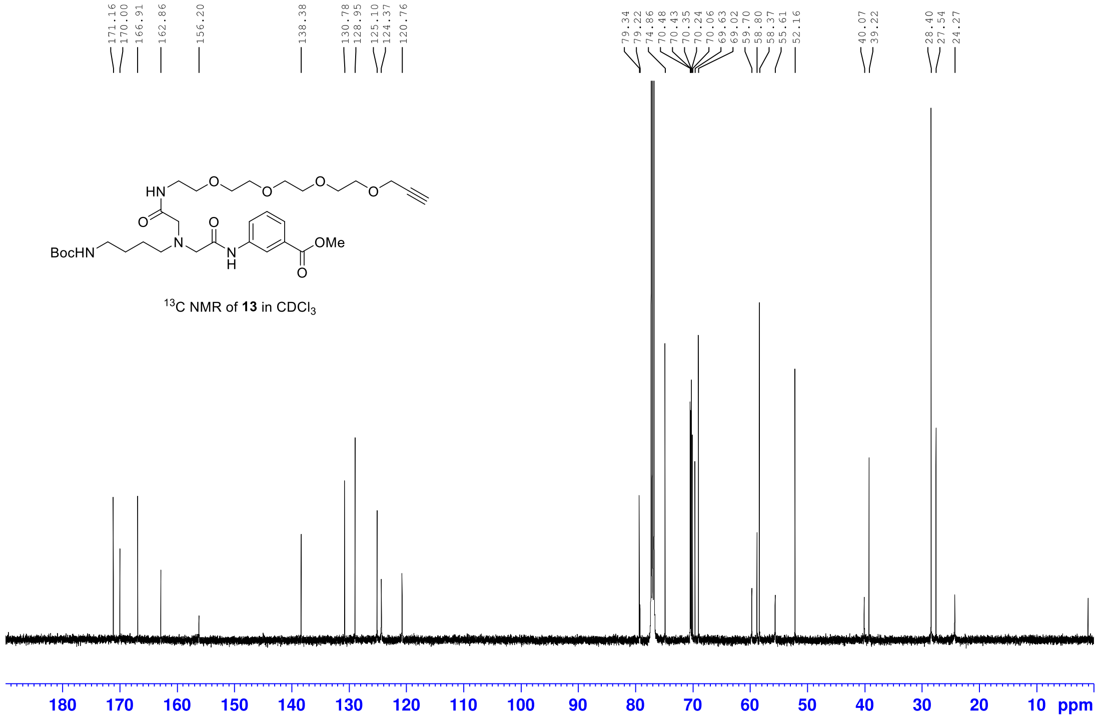

^{13}C NMR of **13** in $CDCl_3$

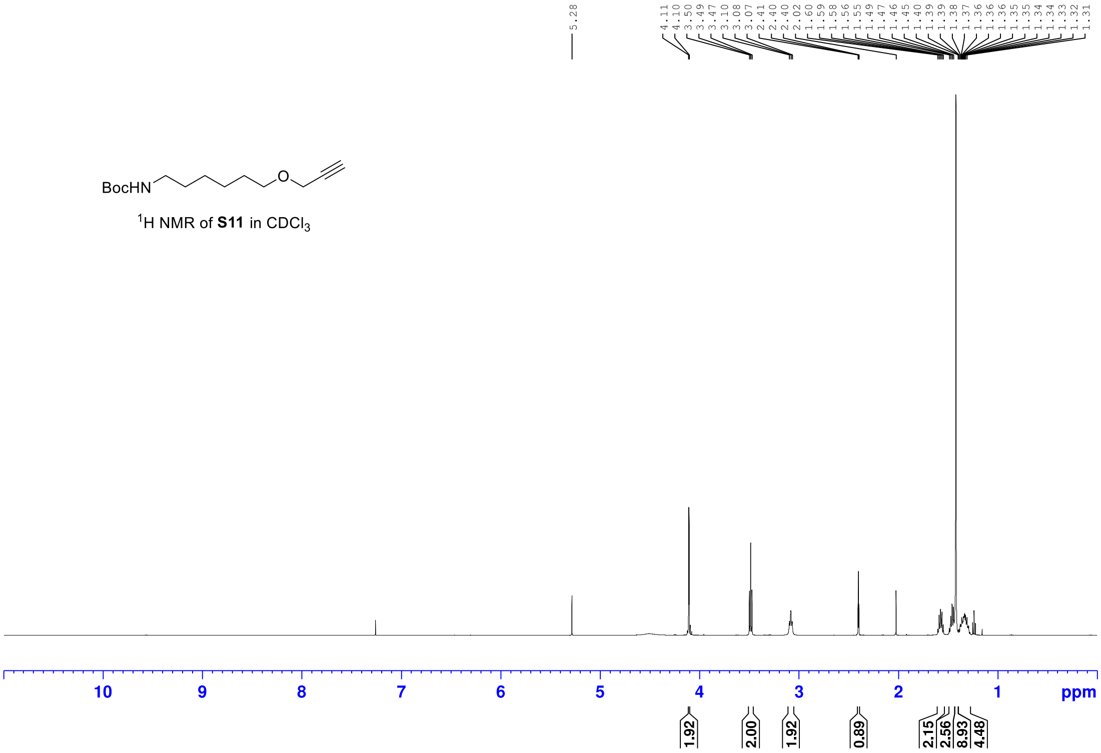

^{1}H NMR of **S11** in $CDCl_3$

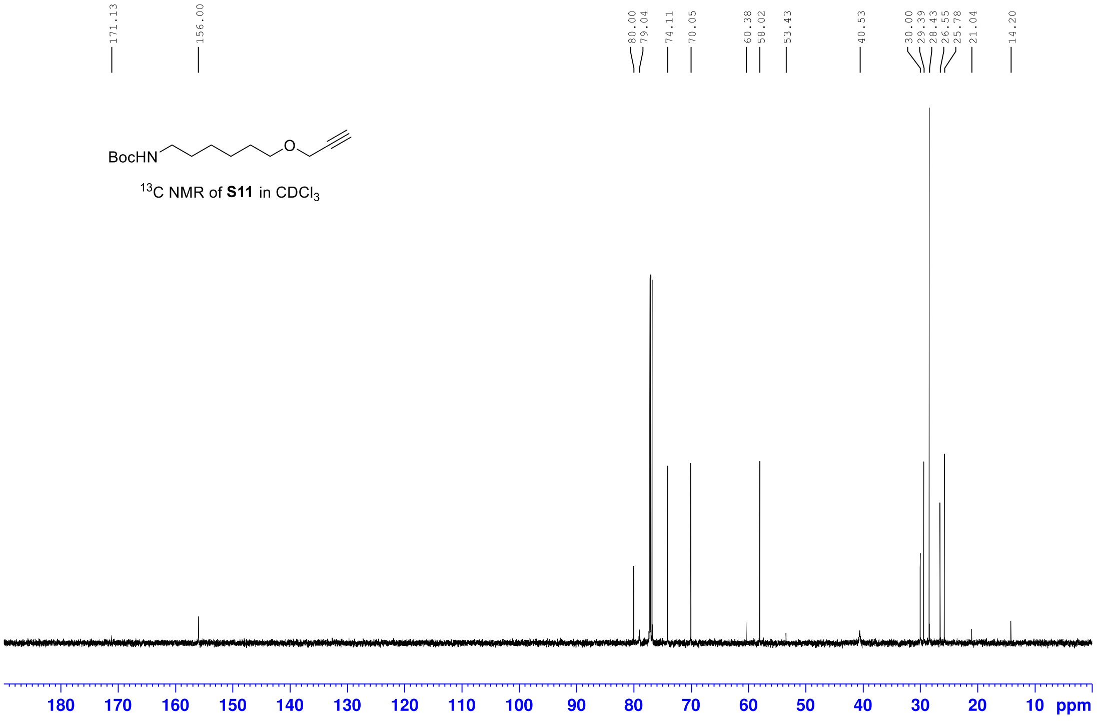

^{13}C NMR of **S11** in $CDCl_3$

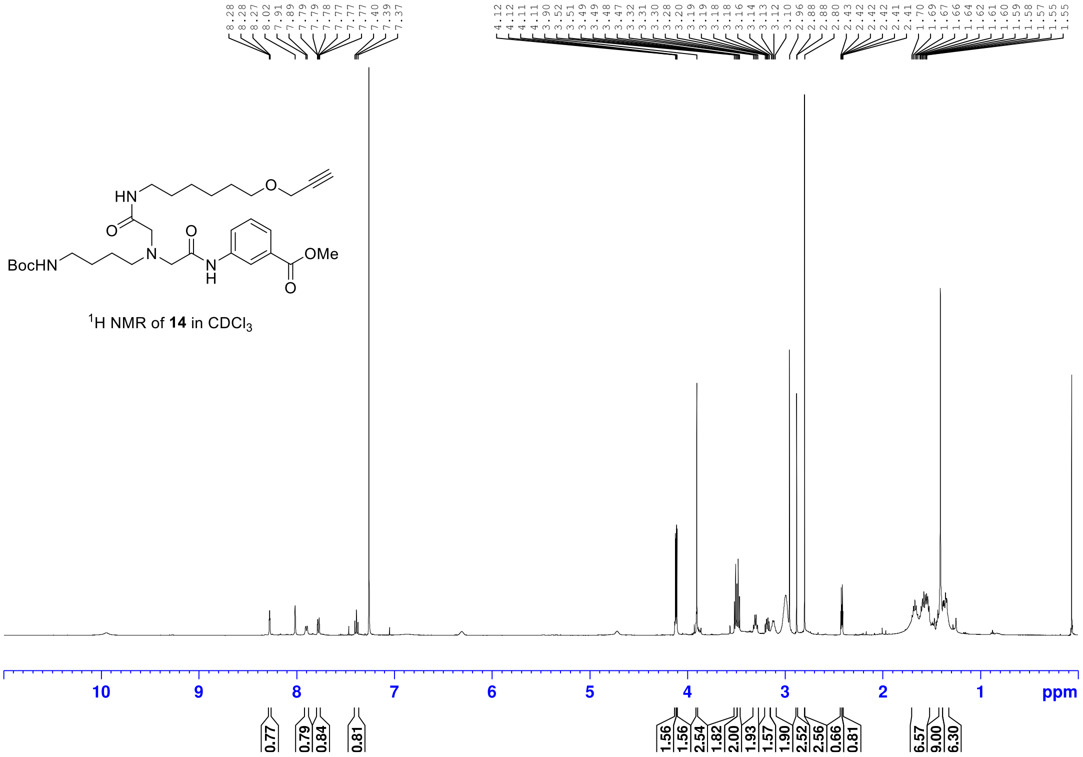

^{1}H NMR of **14** in $CDCl_3$

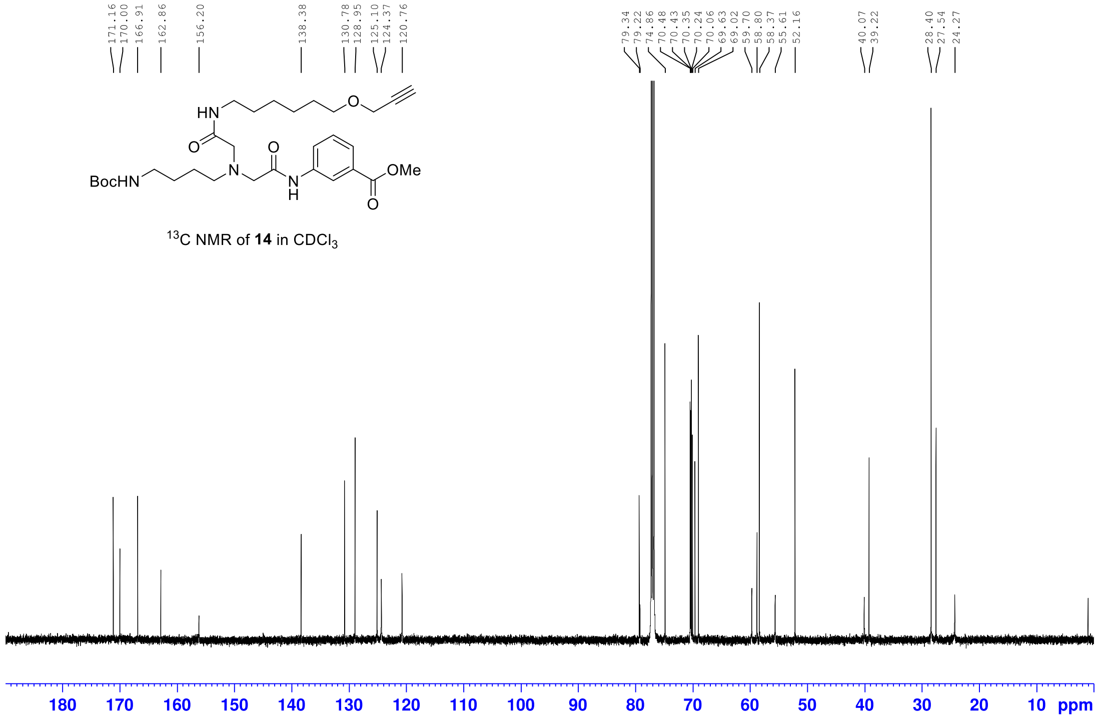

^{13}C NMR of **14** in $CDCl_3$

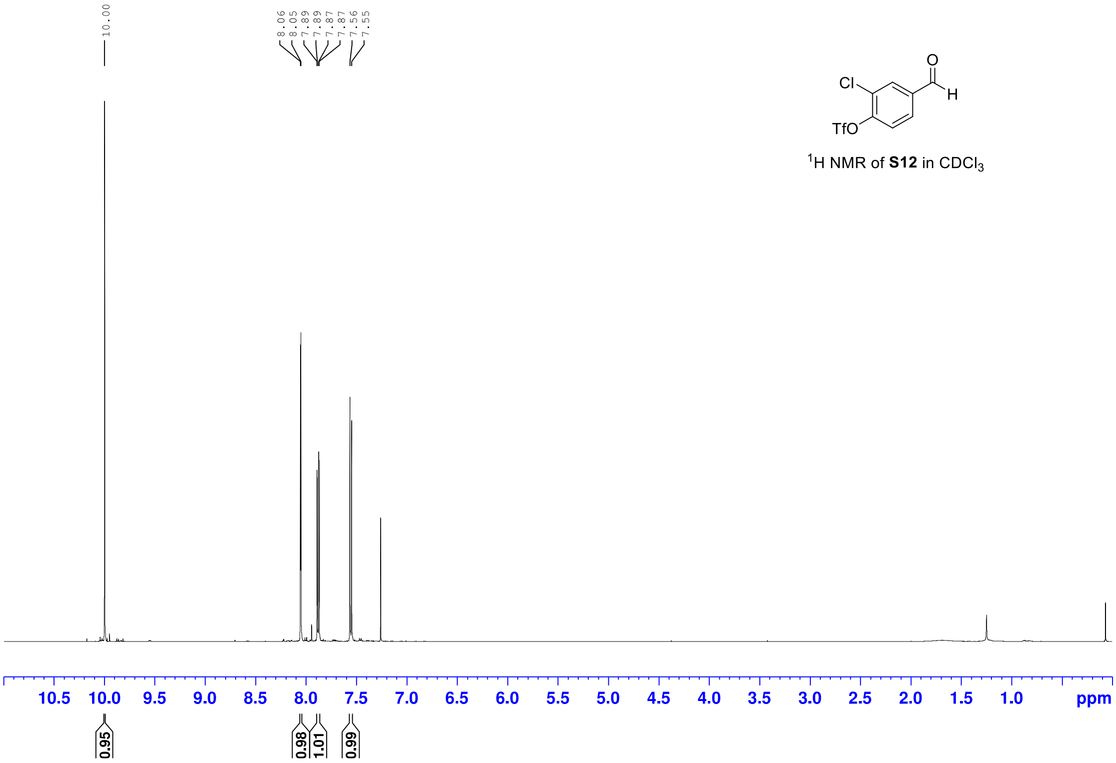
10.00
8.06
8.05
7.89
7.89
7.87
7.87
7.56
7.55
Cl
TfO
O
H
^{1}H NMR of **S12** in $CDCl_3$
10.5 10.0 9.5 9.0 8.5 8.0 7.5 7.0 6.5 6.0 5.5 5.0 4.5 4.0 3.5 3.0 2.5 2.0 1.5 1.0 ppm
0.95
0.98
1.01
0.99

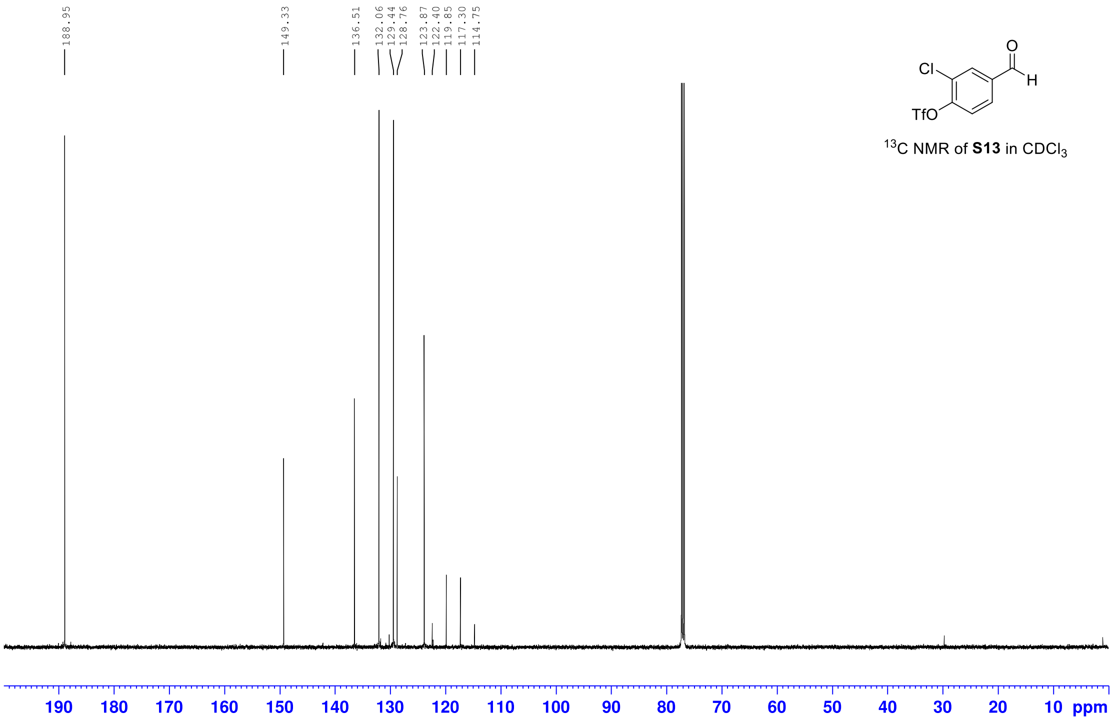
188.95
149.33
136.51
132.06
129.44
128.76
123.87
122.40
119.85
117.30
114.75
Cl
TfO
O
H
^{13}C NMR of **S13** in $CDCl_3$
190 180 170 160 150 140 130 120 110 100 90 80 70 60 50 40 30 20 10 ppm

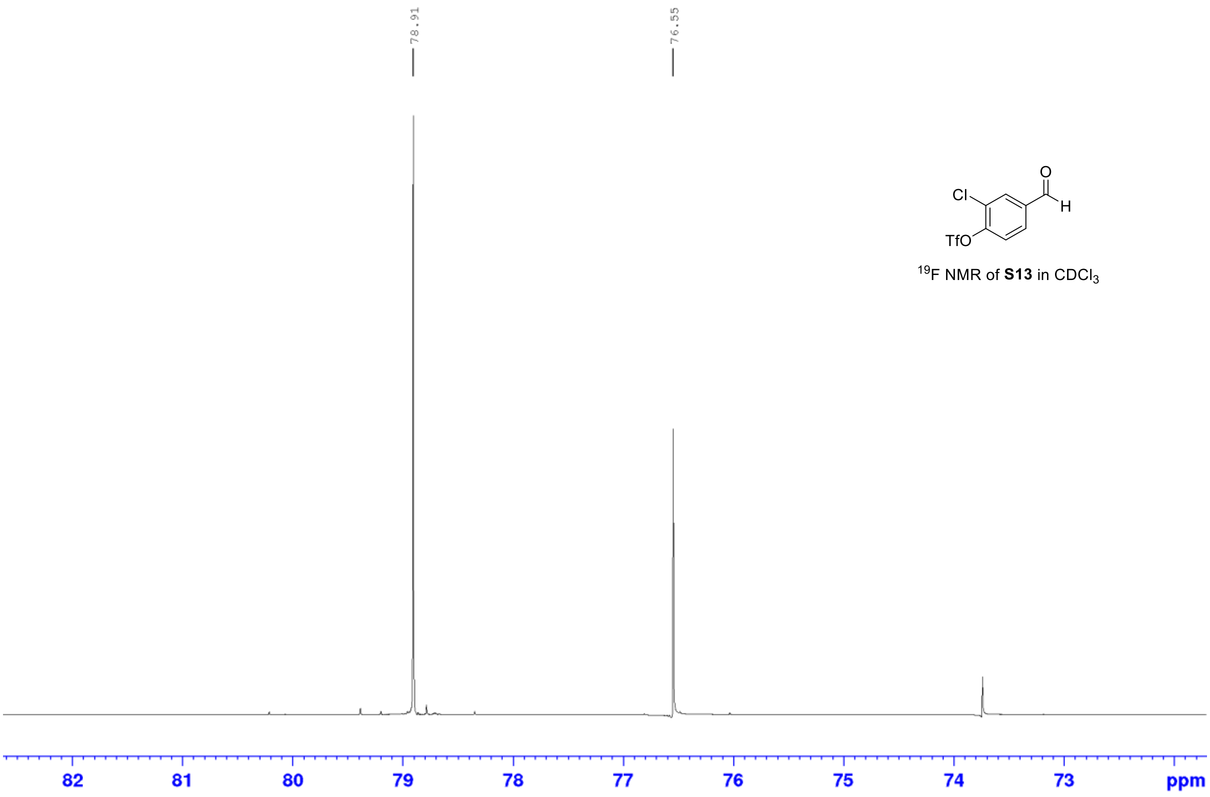

^{19}F NMR of **S13** in $CDCl_3$

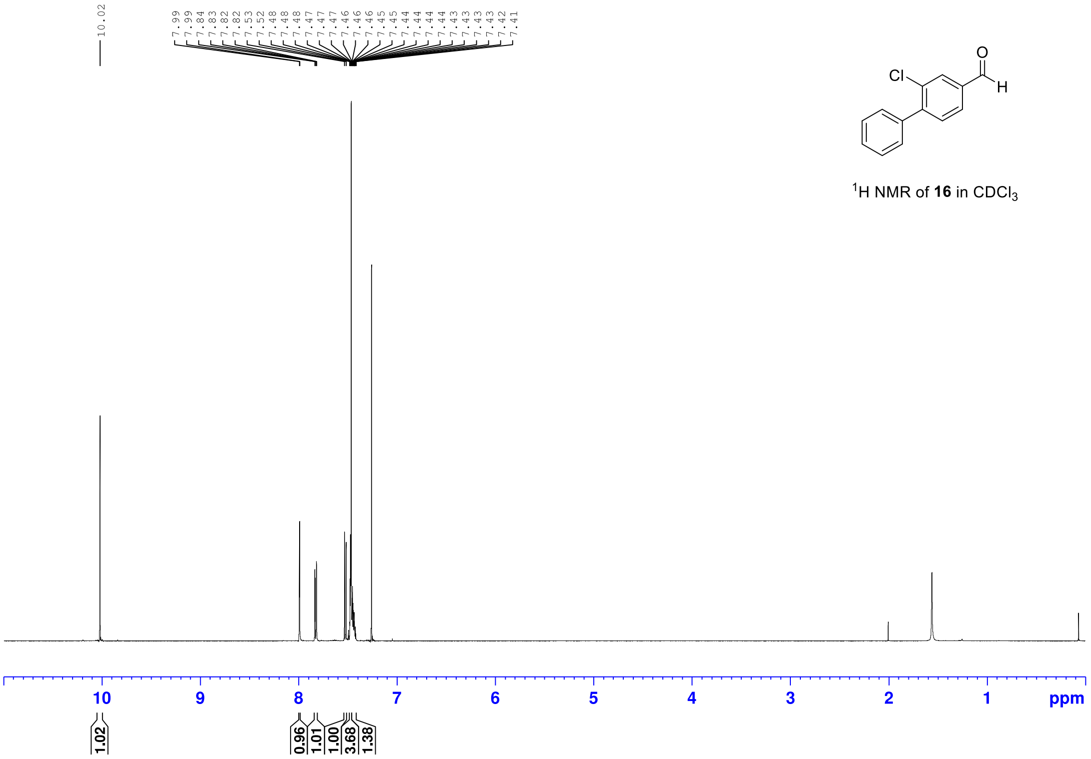

^1H NMR of **16** in $CDCl_3$

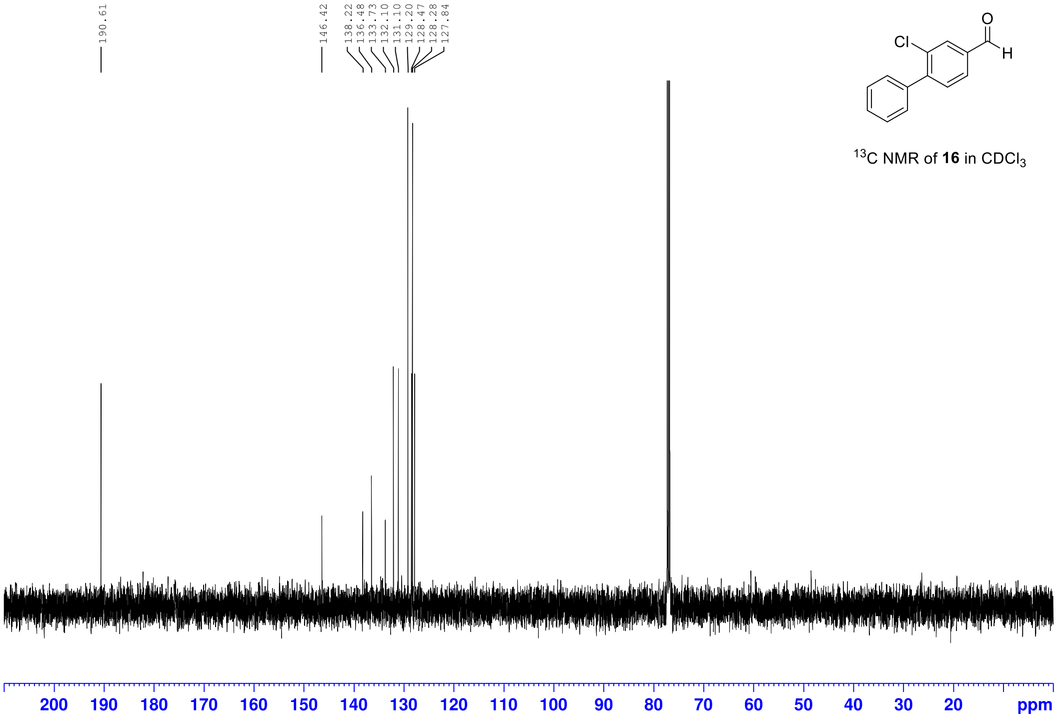

^{13}C NMR of **16** in $CDCl_3$

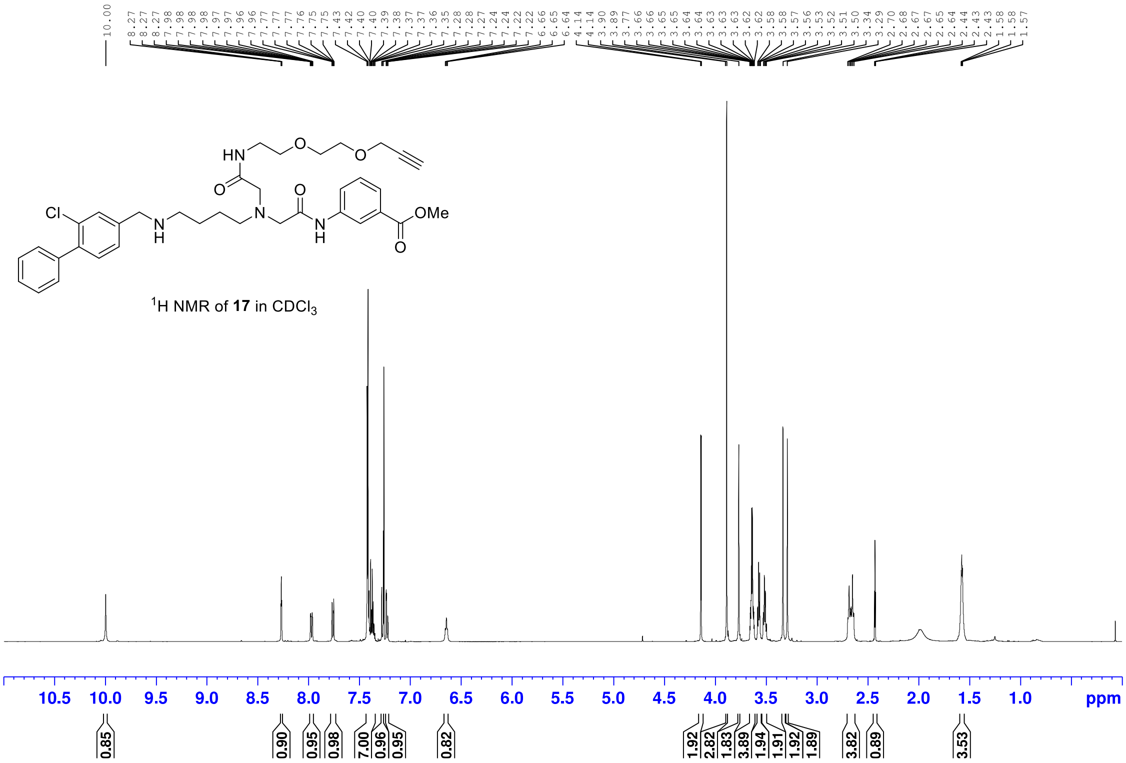
^{1}H NMR of **17** in $CDCl_3$
ppm

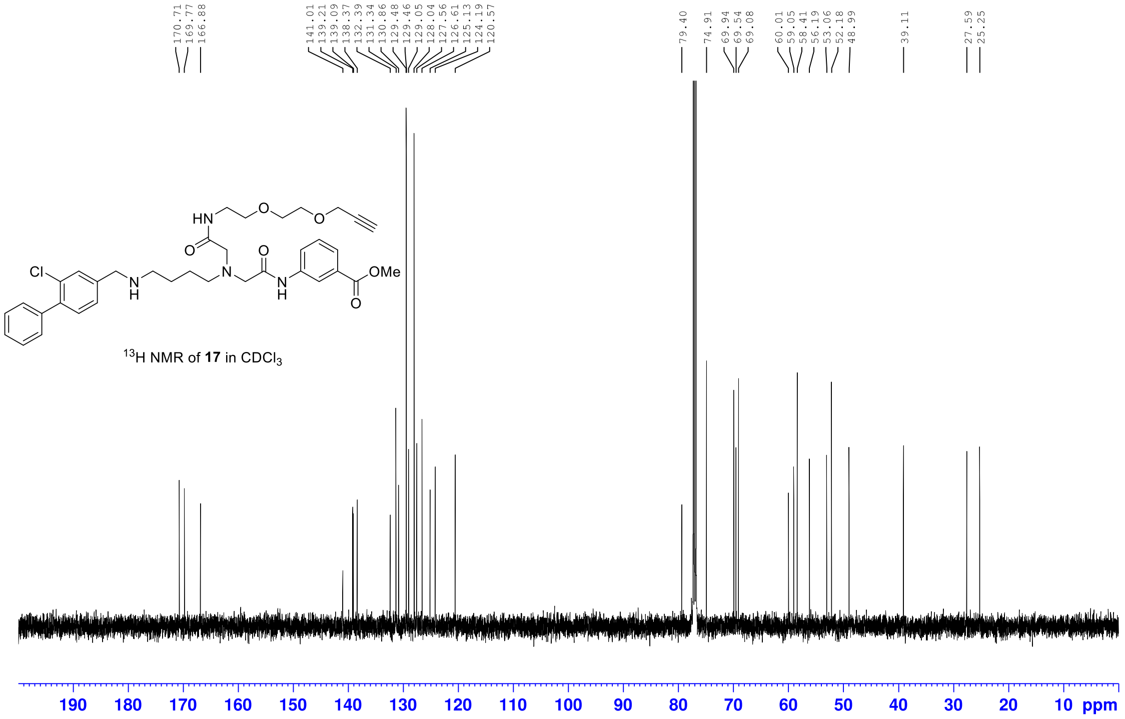
^{13}H NMR of **17** in $CDCl_3$
ppm

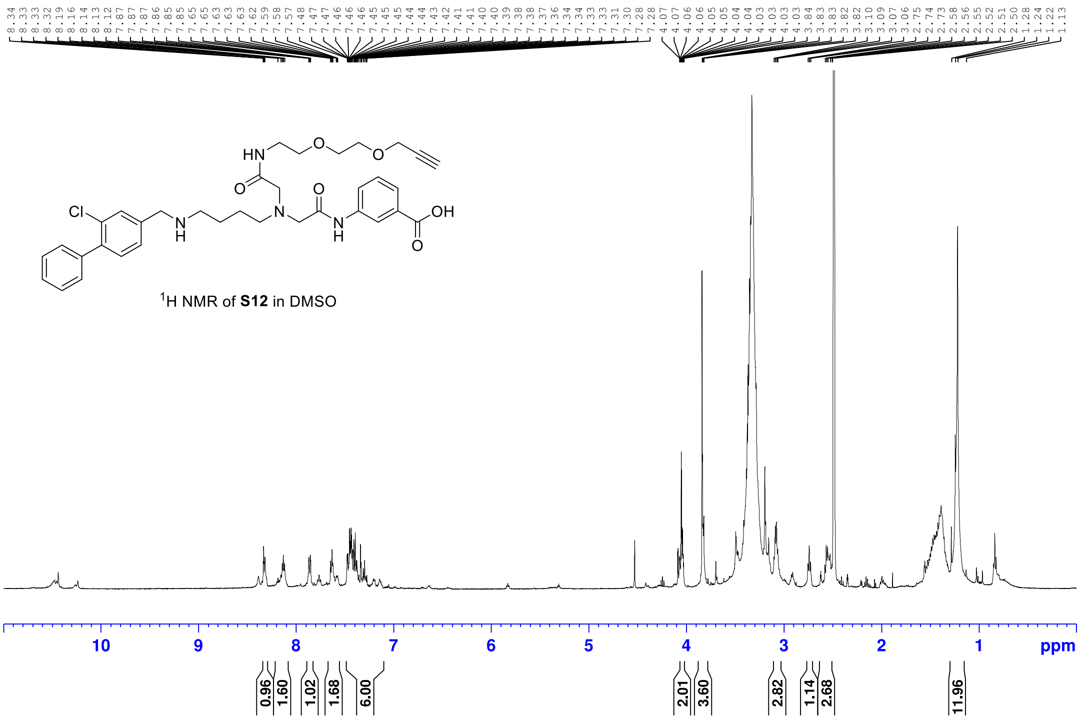

^{1}H NMR of **S12** in DMSO

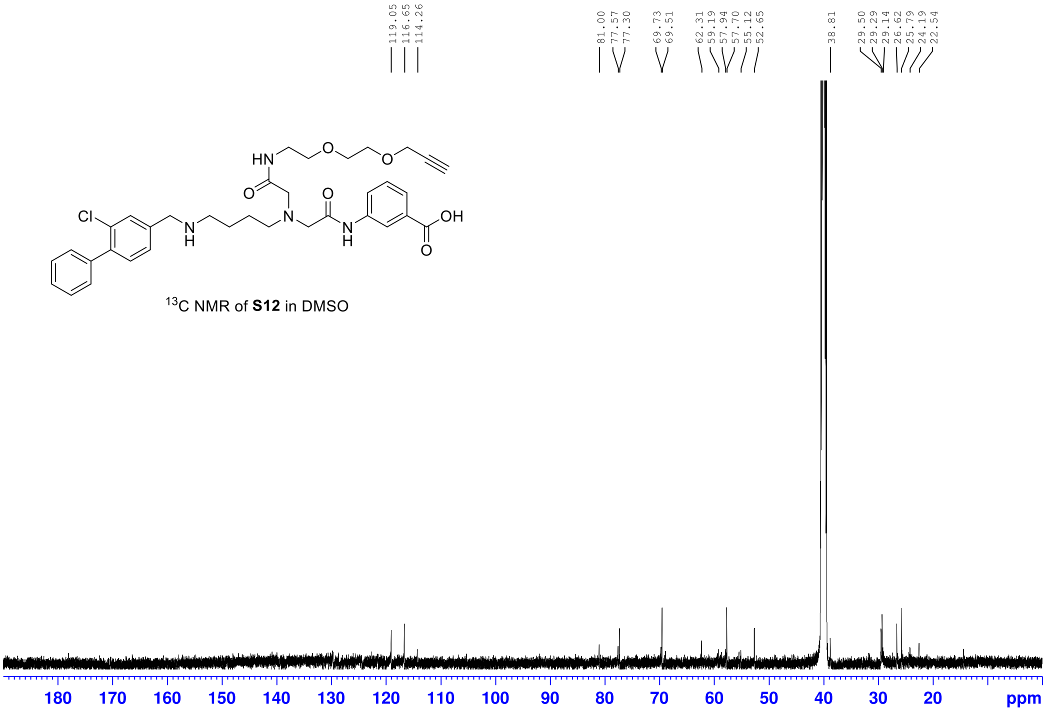

^{13}C NMR of **S12** in DMSO

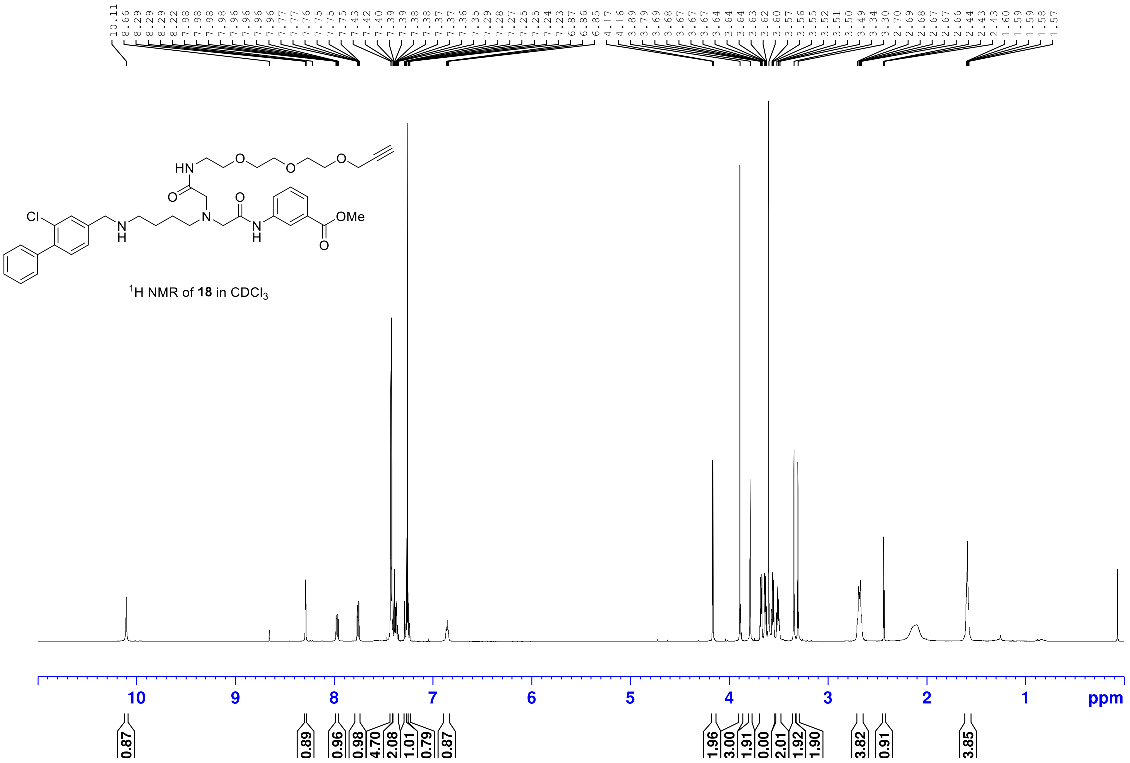

^{1}H NMR of **18** in $CDCl_3$

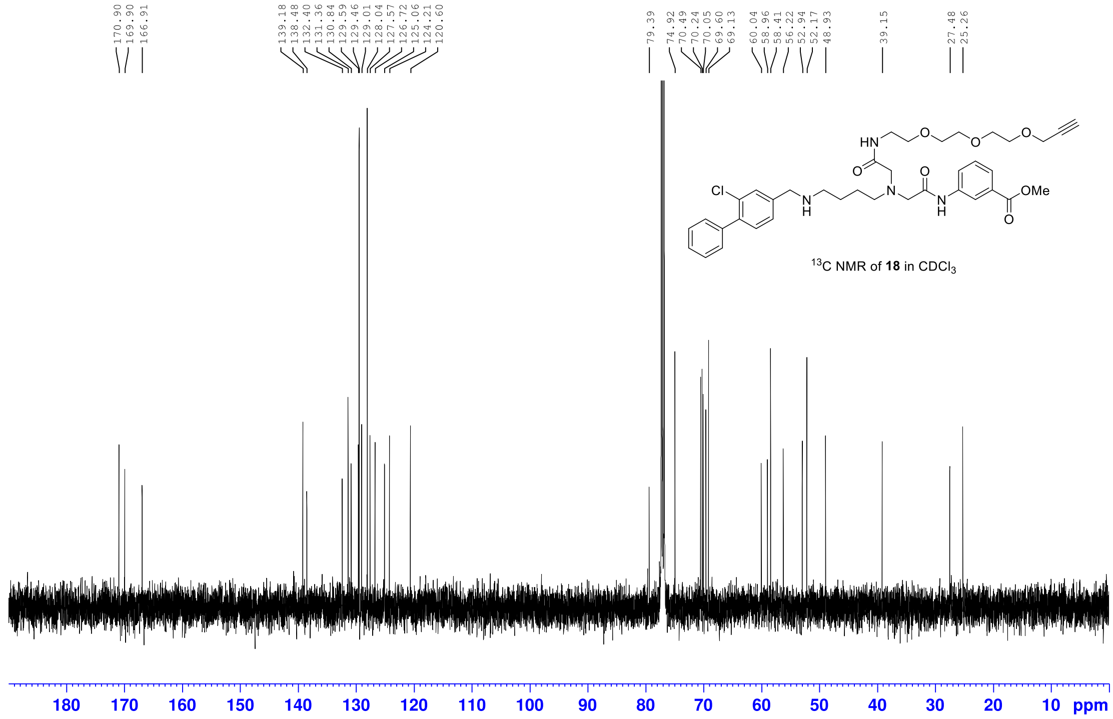

^{13}C NMR of **18** in $CDCl_3$

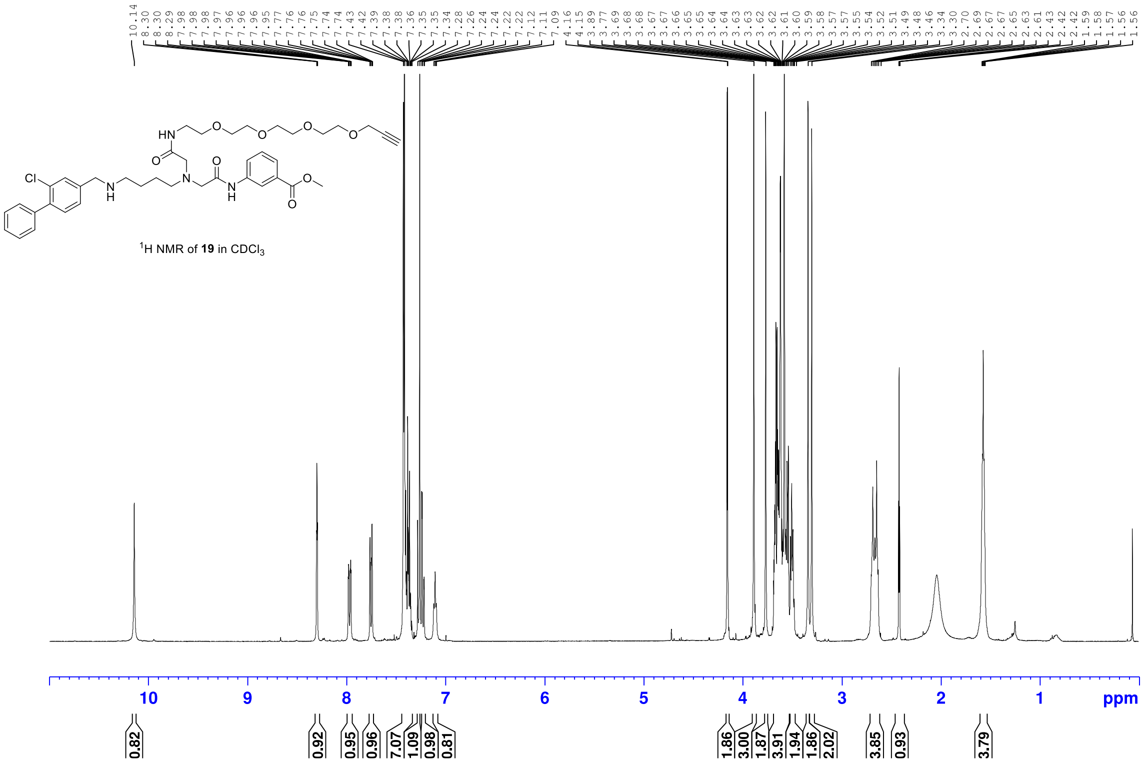
10.14 8.30 8.30 8.29 7.98 7.98 7.97 7.96 7.96 7.96 7.95 7.77 7.76 7.76 7.75 7.74 7.74 7.43 7.42 7.39 7.38 7.38 7.36 7.35 7.35 7.34 7.28 7.26 7.24 7.24 7.22 7.22 7.12 7.11 7.09 4.16 4.15 3.89 3.77 3.69 3.68 3.68 3.67 3.67 3.66 3.65 3.65 3.64 3.63 3.63 3.62 3.62 3.61 3.60 3.59 3.58 3.57 3.57 3.55 3.54 3.52 3.51 3.49 3.48 3.46 3.34 3.30 2.70 2.69 2.67 2.67 2.65 2.63 2.61 2.43 2.42 2.42 1.59 1.58 1.57 1.56
HN
O
O
O
O
O
Cl
N
N
H
O
N
H
O
O
^{1}H NMR of **19** in $CDCl_3$
10 9 8 7 6 5 4 3 2 1 ppm
0.82 0.92 0.95 0.96 7.07 1.09 0.98 0.81 1.86 3.00 1.87 3.91 1.94 1.86 2.02 3.85 0.93 3.79

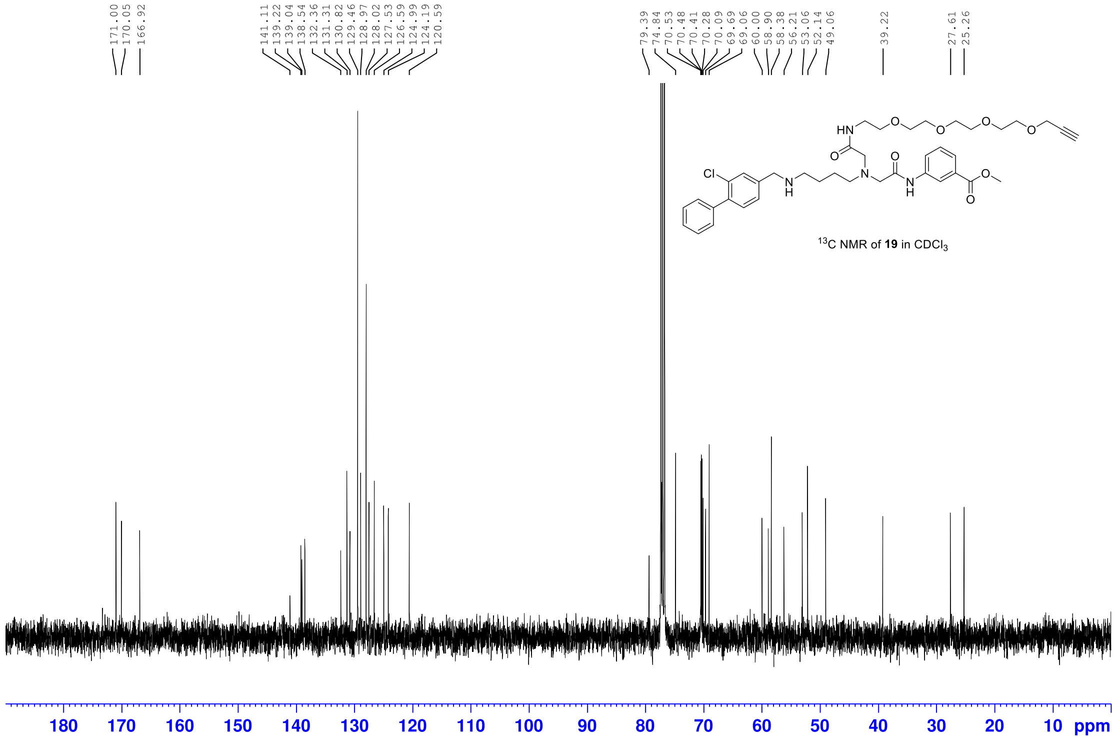
171.00 170.05 166.92 141.11 139.22 139.04 138.54 132.36 131.31 130.82 129.46 128.97 128.02 127.53 126.59 124.99 124.19 120.59 79.39 74.84 70.53 70.48 70.41 70.28 70.09 69.69 69.06 60.00 58.90 58.38 56.21 53.06 52.14 49.06 39.22 27.61 25.26
HN
O
O
O
O
O
Cl
N
N
H
O
N
H
O
O
^{13}C NMR of **19** in $CDCl_3$
180 170 160 150 140 130 120 110 100 90 80 70 60 50 40 30 20 10 ppm

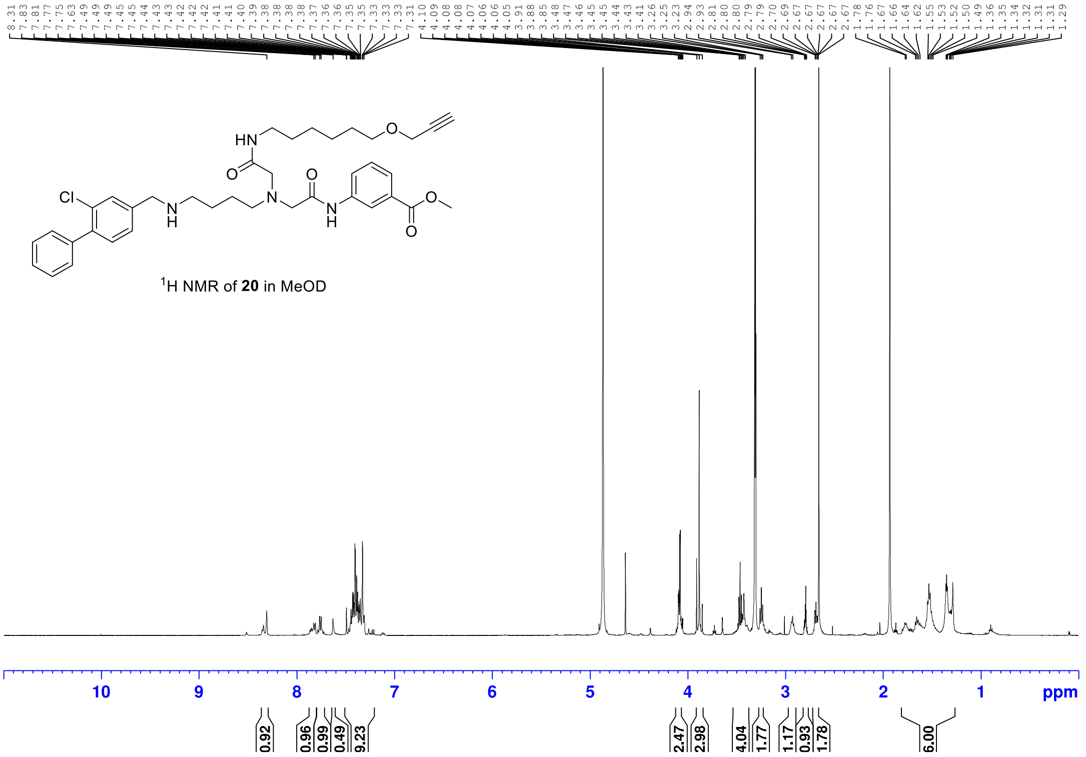

^{1}H NMR of **20** in MeOD

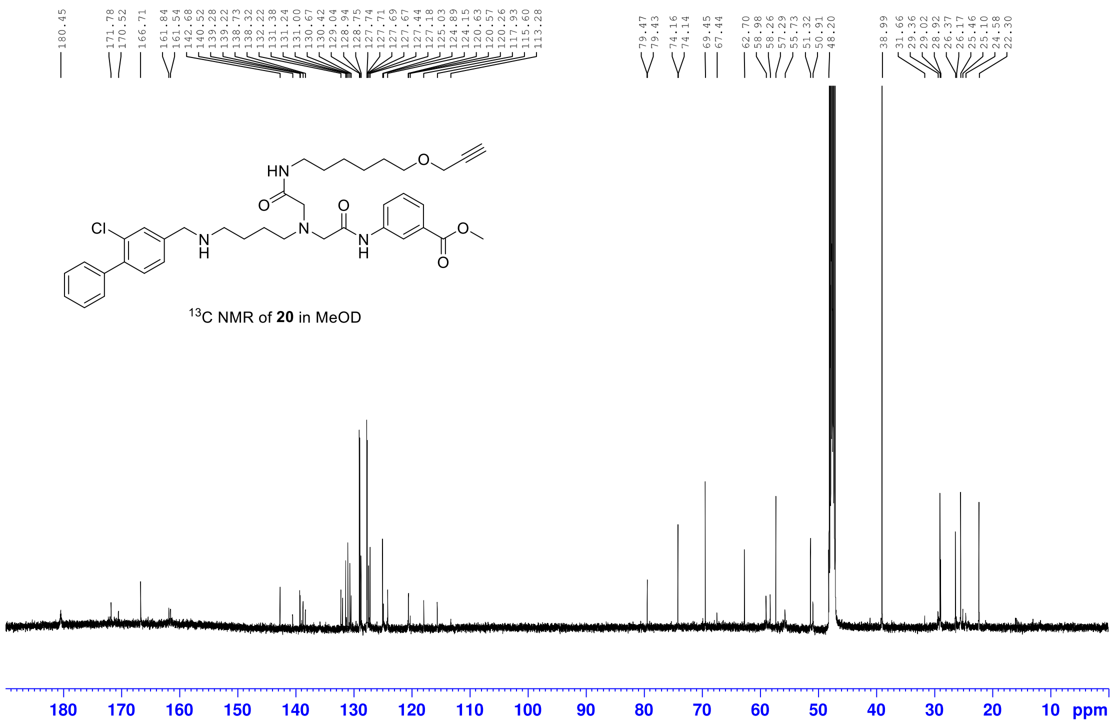

^{13}C NMR of **20** in MeOD

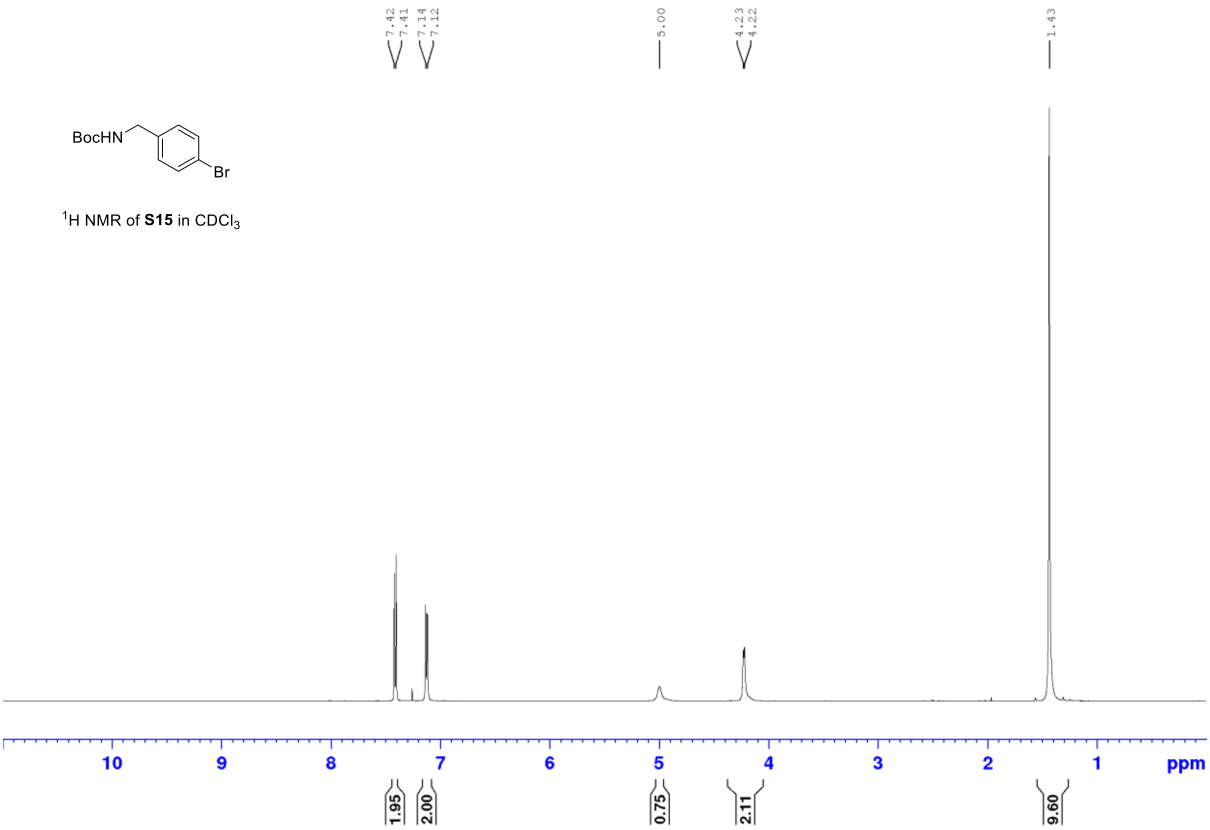

7.42
7.41
7.14
7.12
5.00
4.23
4.22
1.43
BocHN
Br
^{1}H NMR of **S15** in $CDCl_3$
10
9
8
7
6
5
4
3
2
1
ppm
1.95
2.00
0.75
2.11
9.60

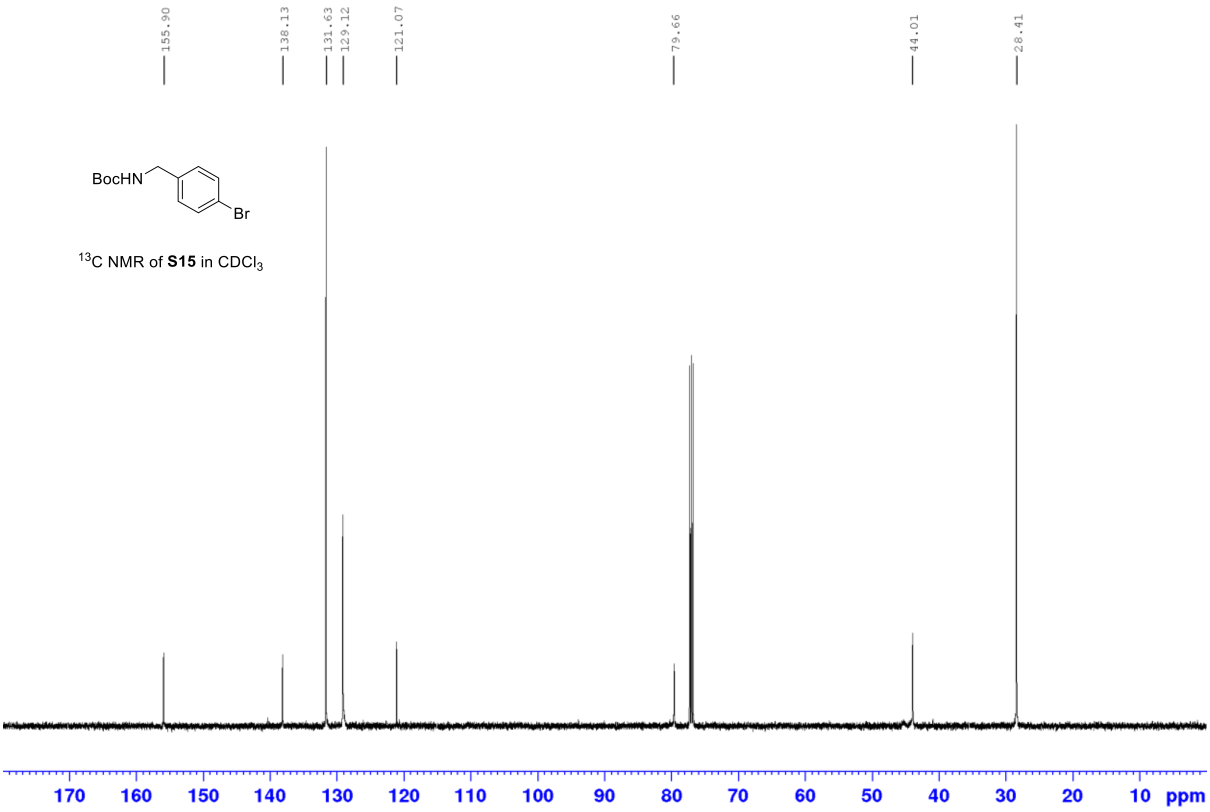

155.90
138.13
131.63
129.12
121.07
79.66
44.01
28.41
BocHN
Br
^{13}C NMR of **S15** in $CDCl_3$
170
160
150
140
130
120
110
100
90
80
70
60
50
40
30
20
10
ppm

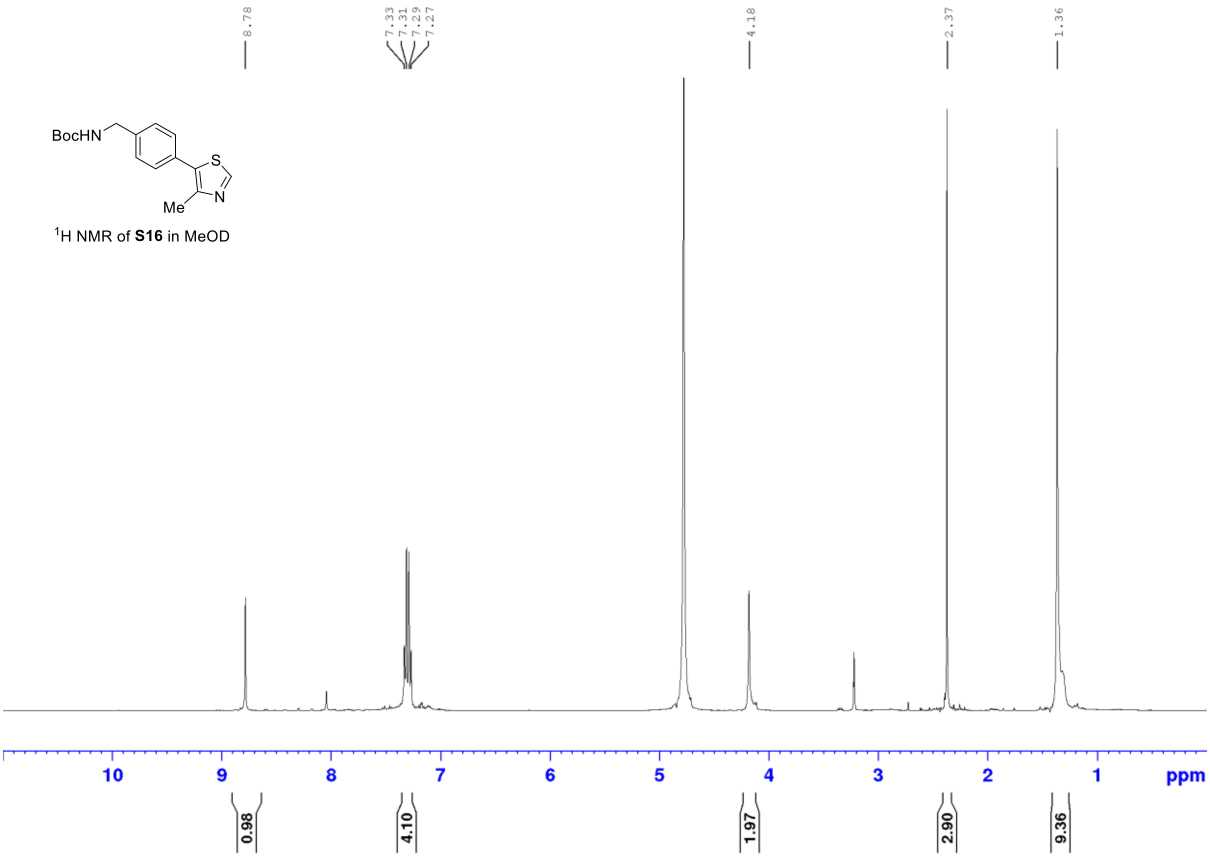

^{1}H NMR of **S16** in MeOD

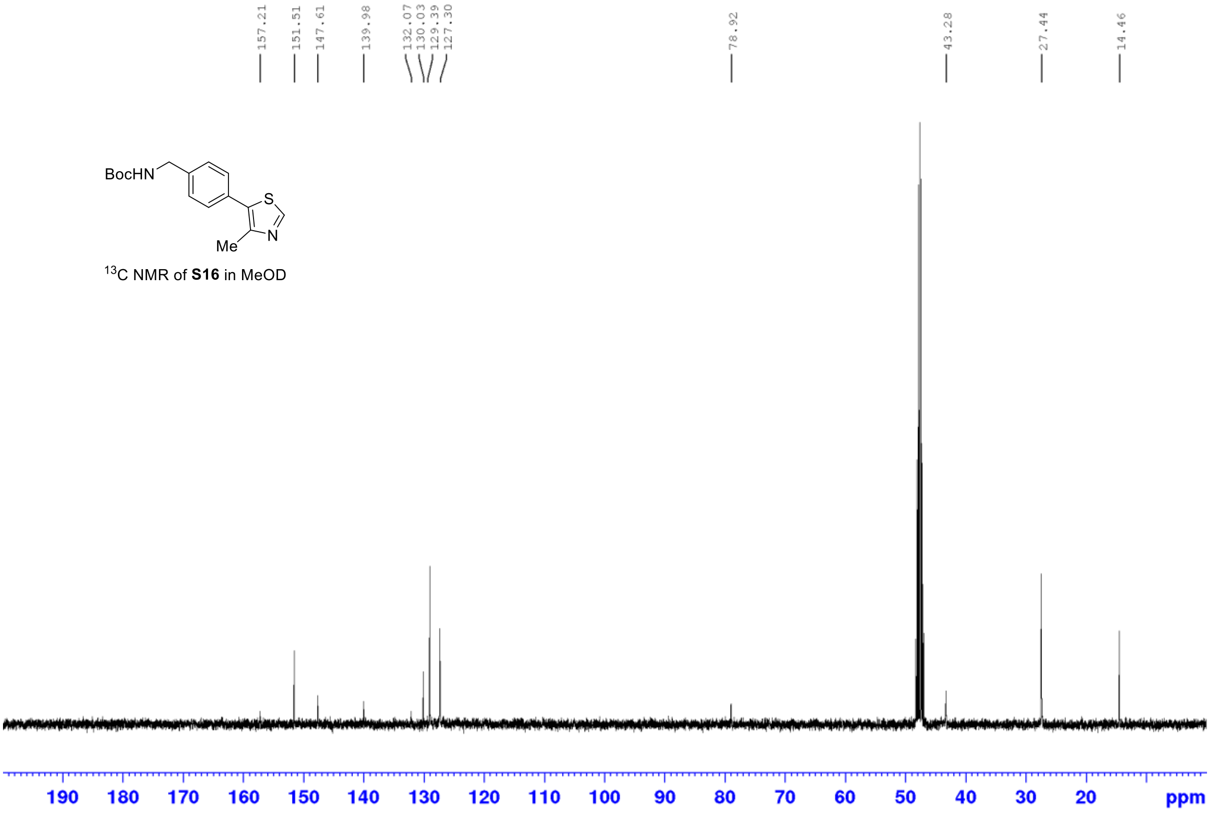

^{13}C NMR of **S16** in MeOD

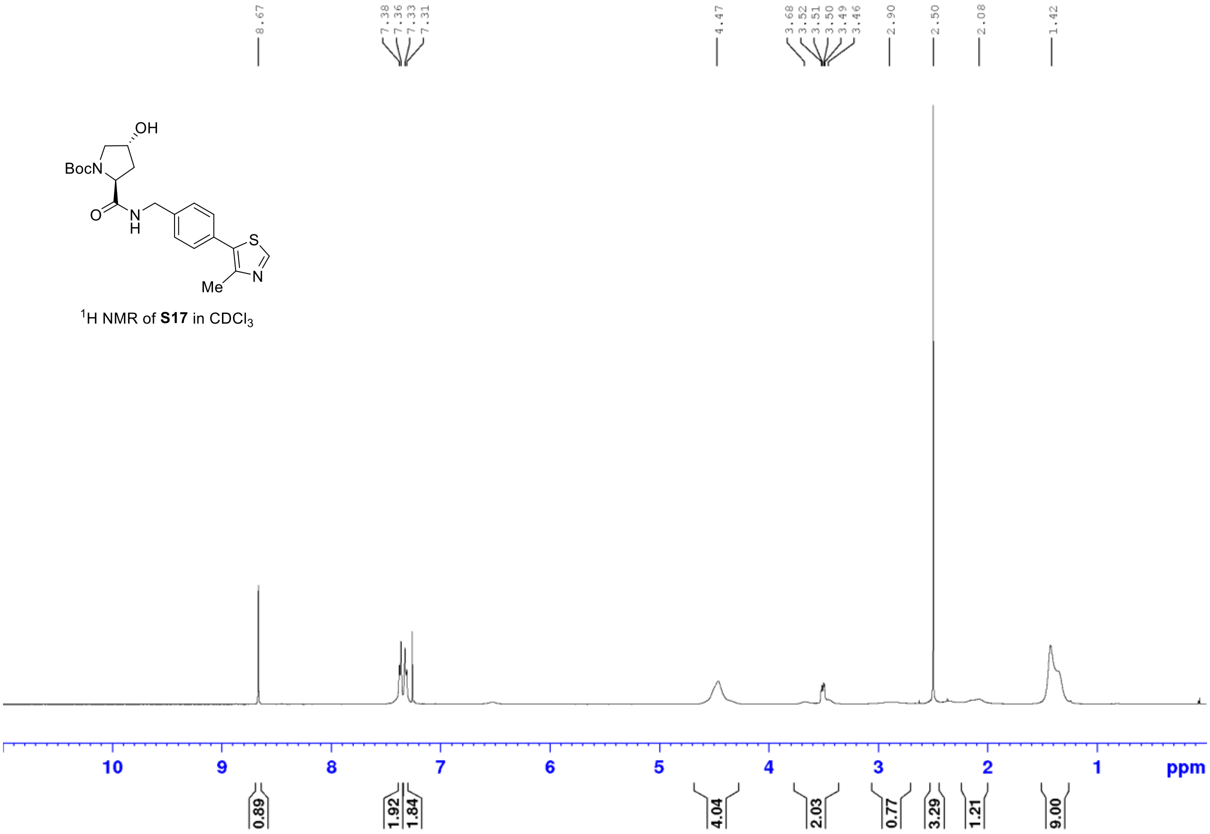

^{1}H NMR of **S17** in $CDCl_3$

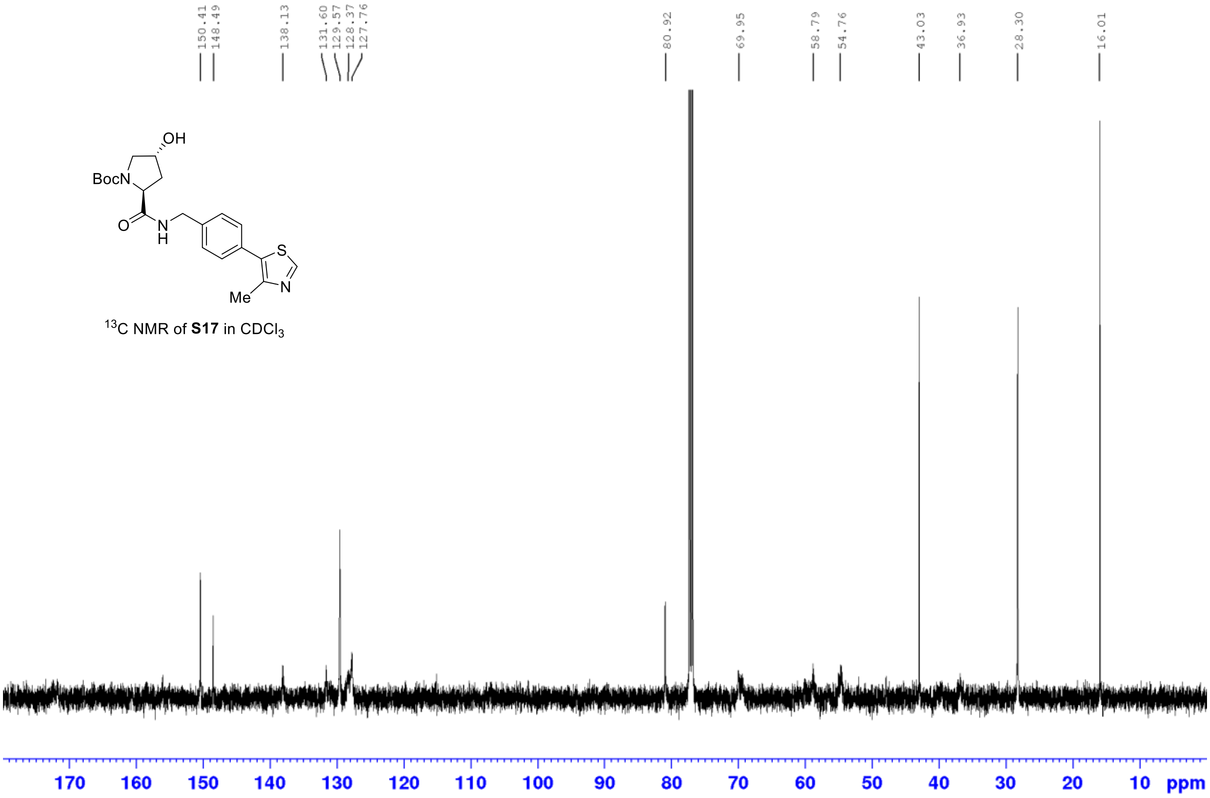

^{13}C NMR of **S17** in $CDCl_3$

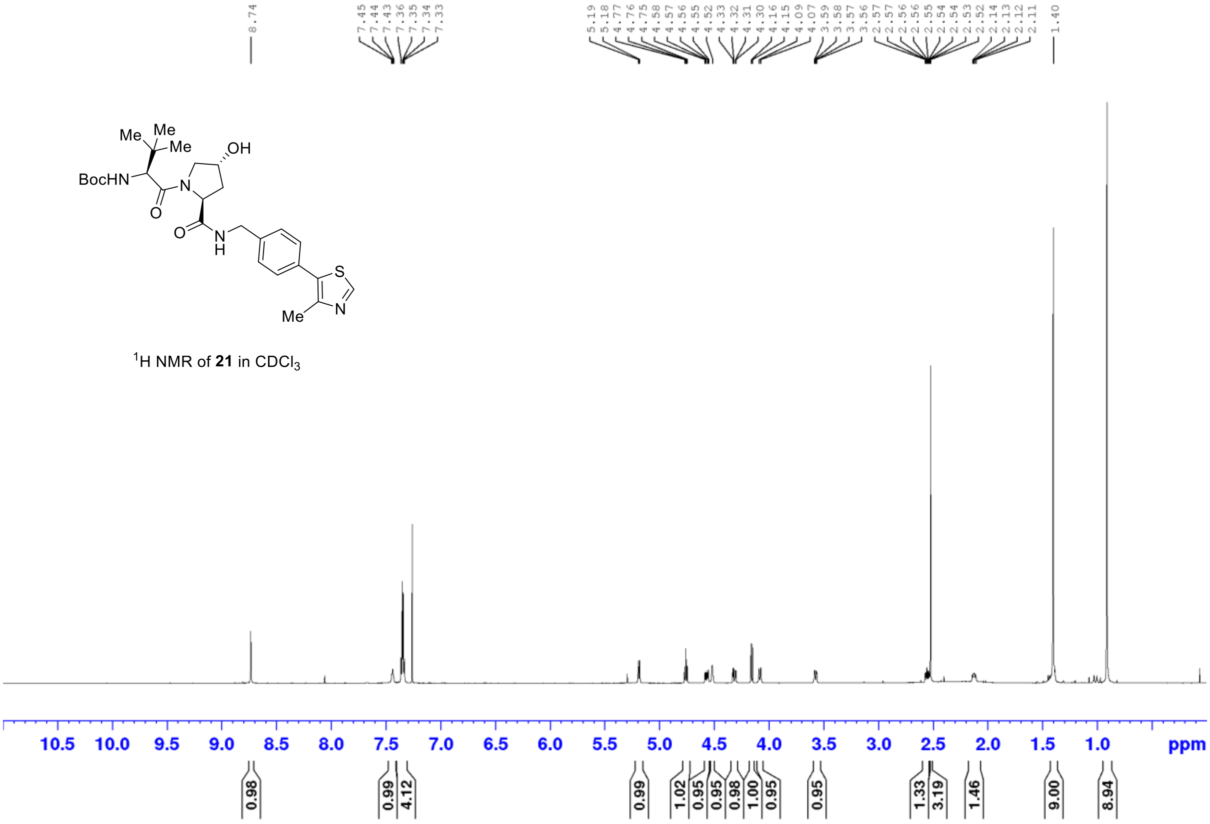

^{1}H NMR of **21** in $CDCl_3$

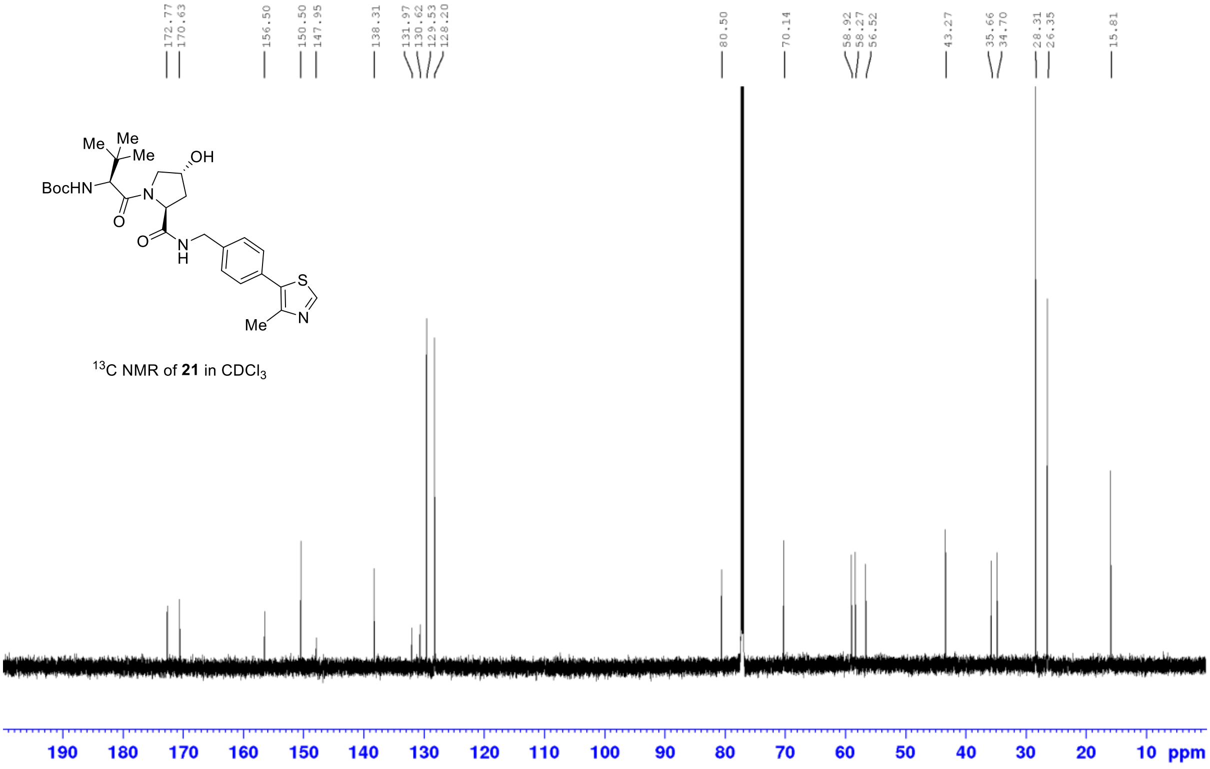

^{13}C NMR of **21** in $CDCl_3$

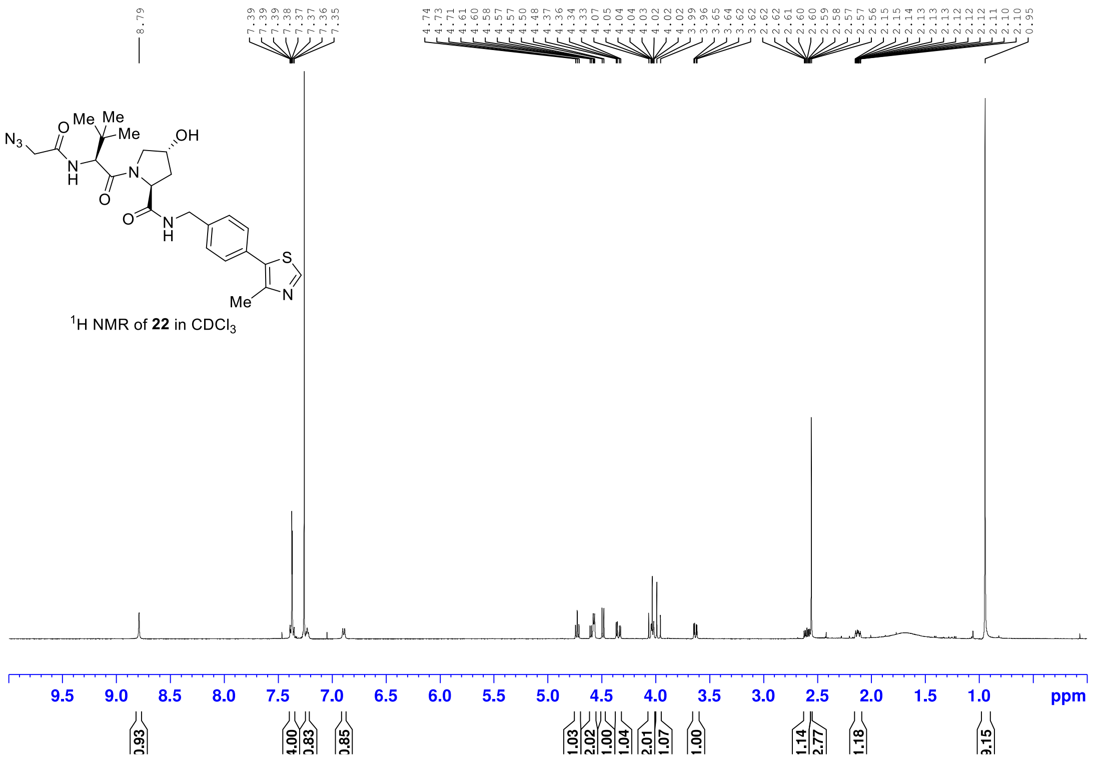
^{1}H NMR of **22** in $CDCl_3$
ppm

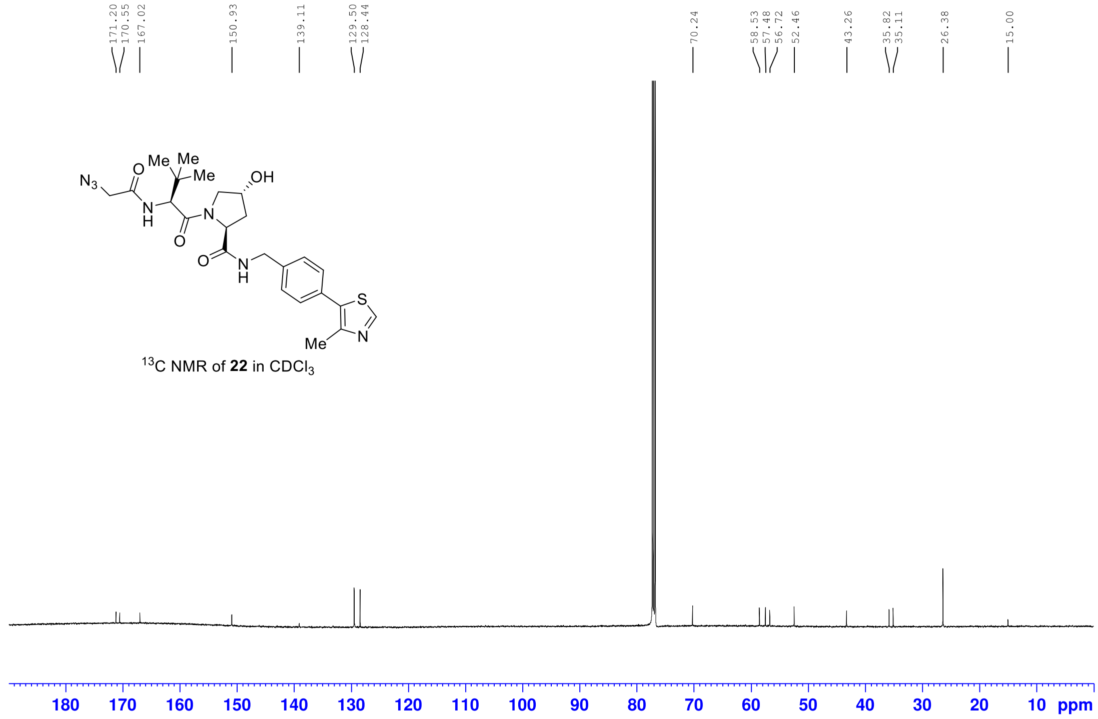
^{13}C NMR of **22** in $CDCl_3$
ppm

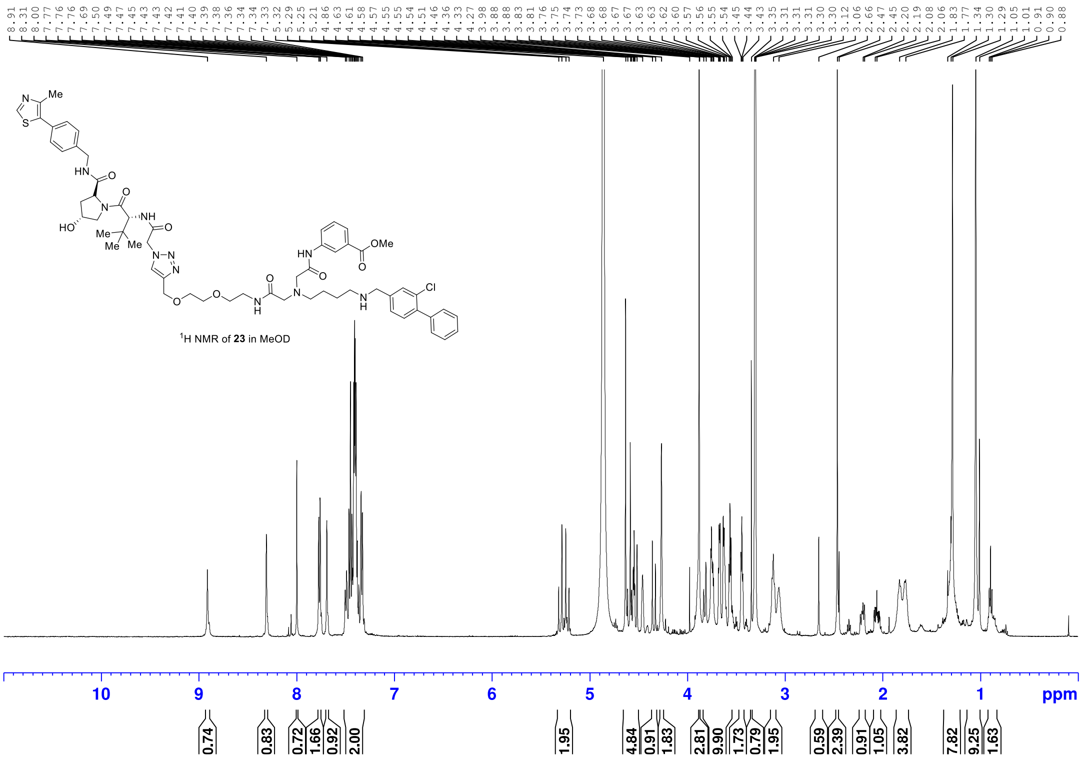
1H NMR of 23 in MeOD

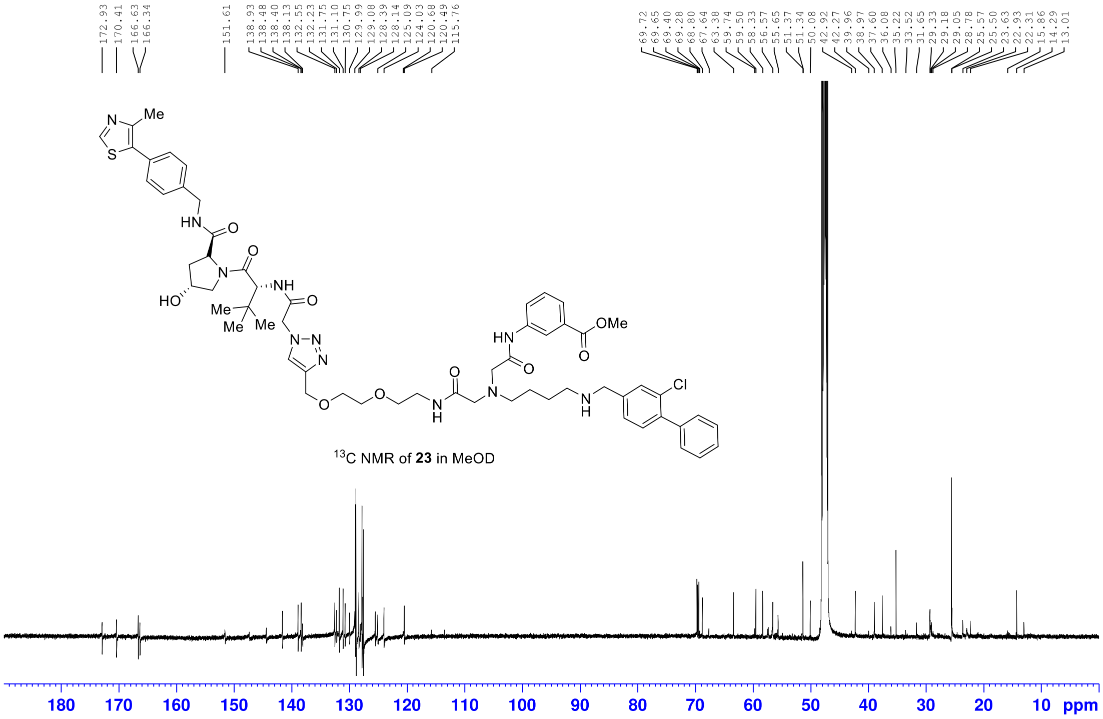
13C NMR of 23 in MeOD

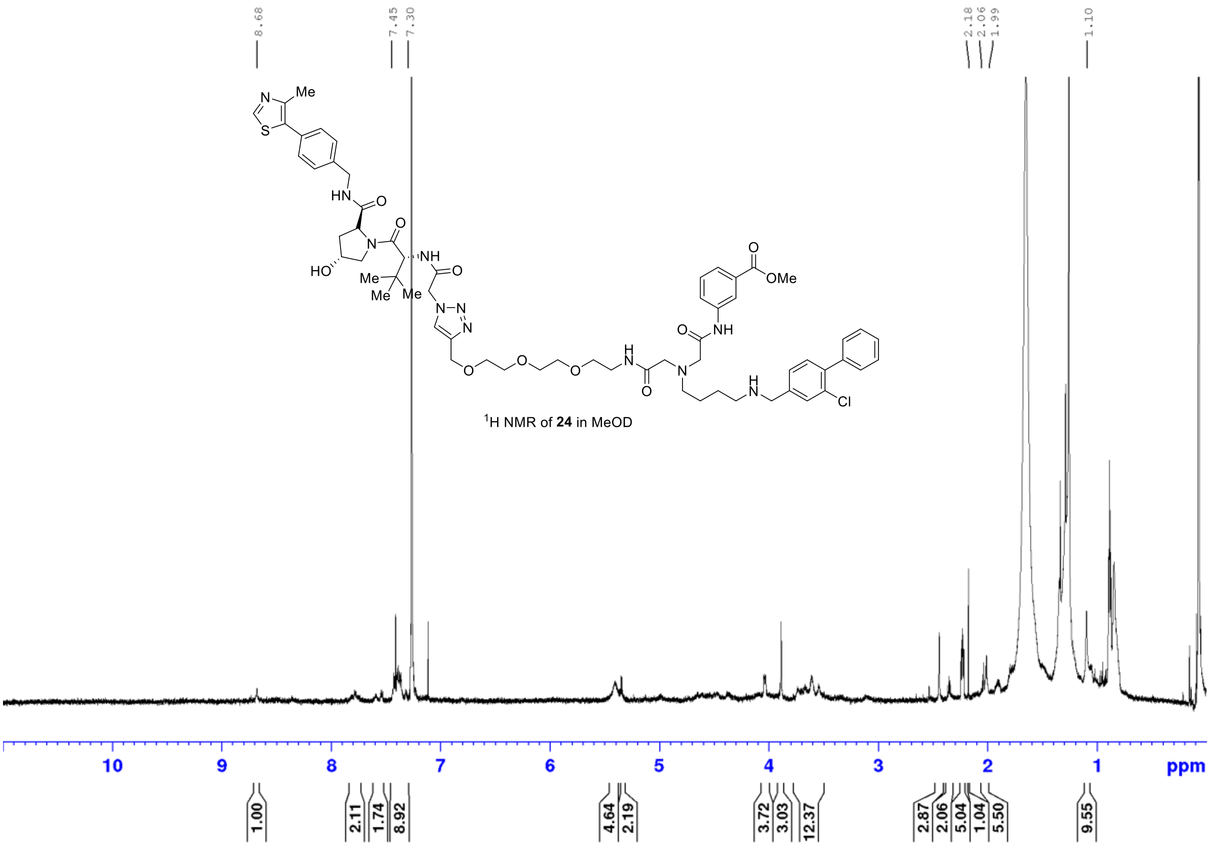

^{1}H NMR of **24** in MeOD

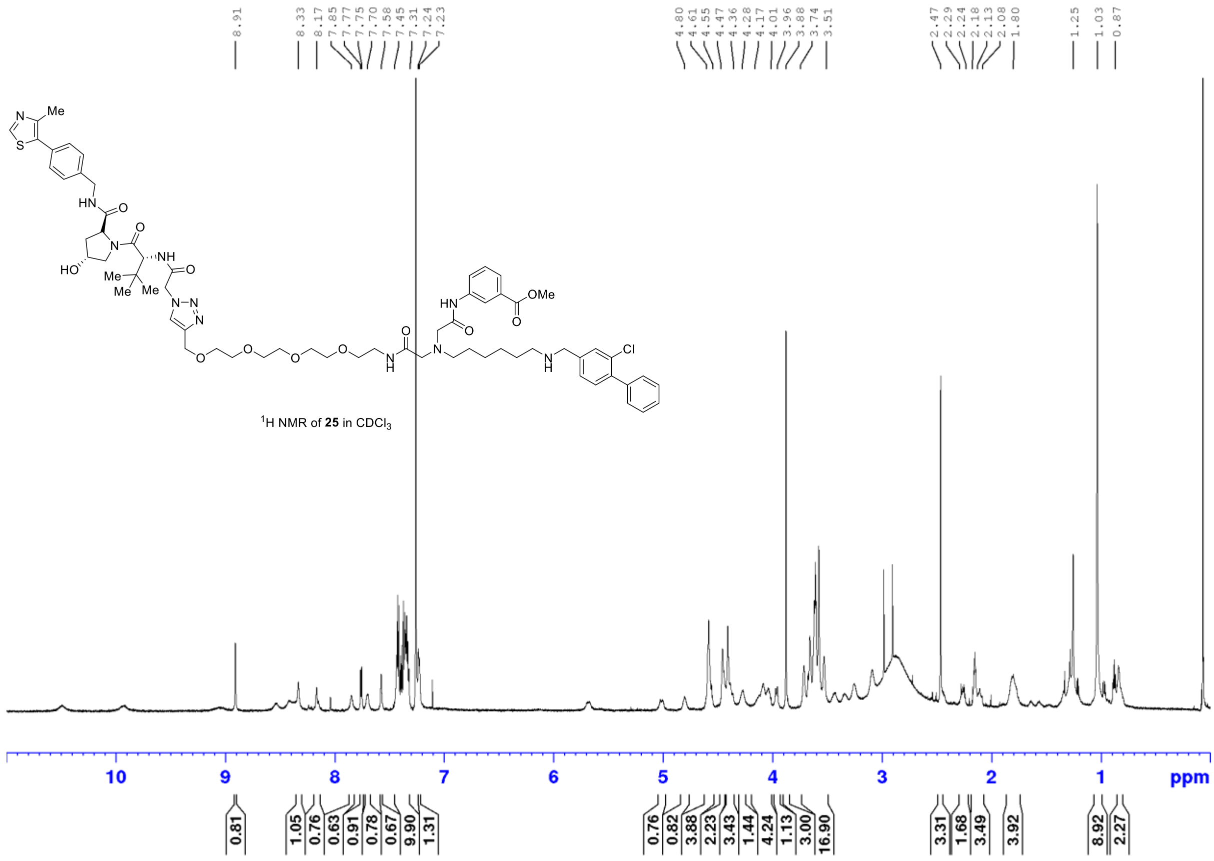

^{1}H NMR of **25** in $CDCl_3$

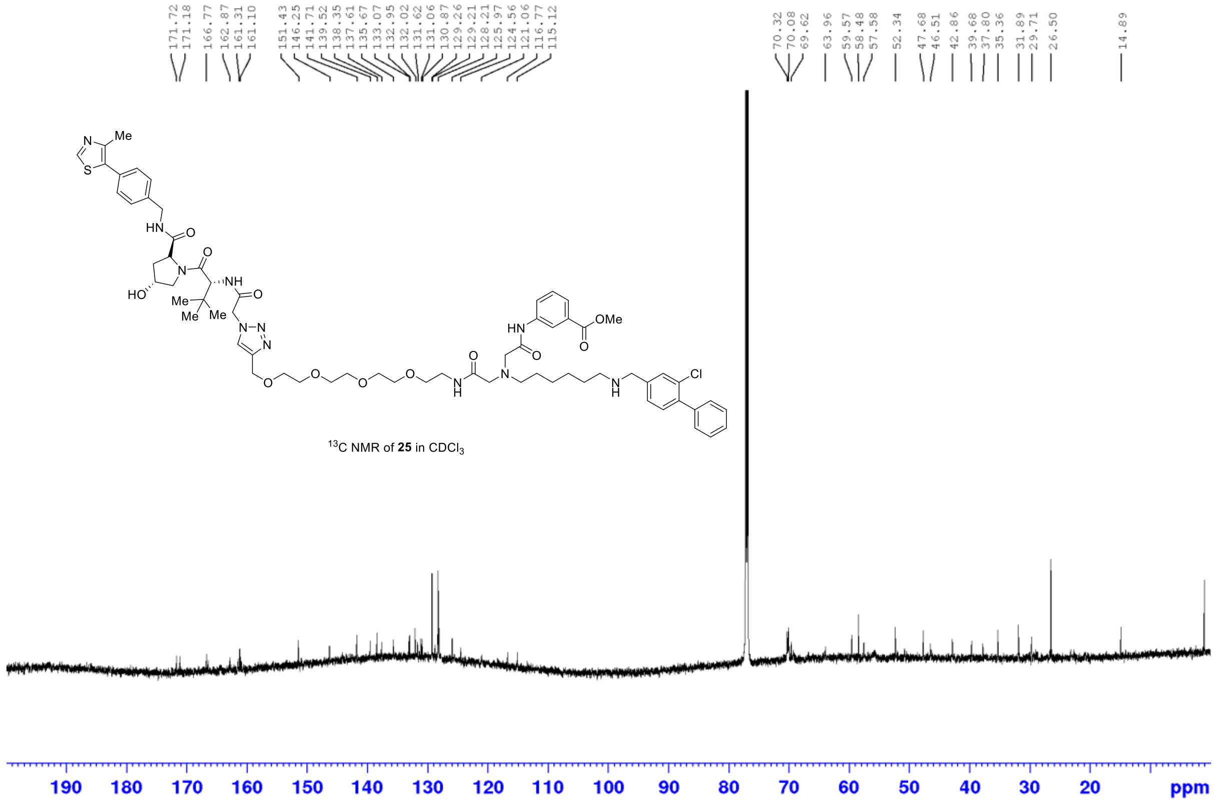

^{13}C NMR of **25** in $CDCl_3$

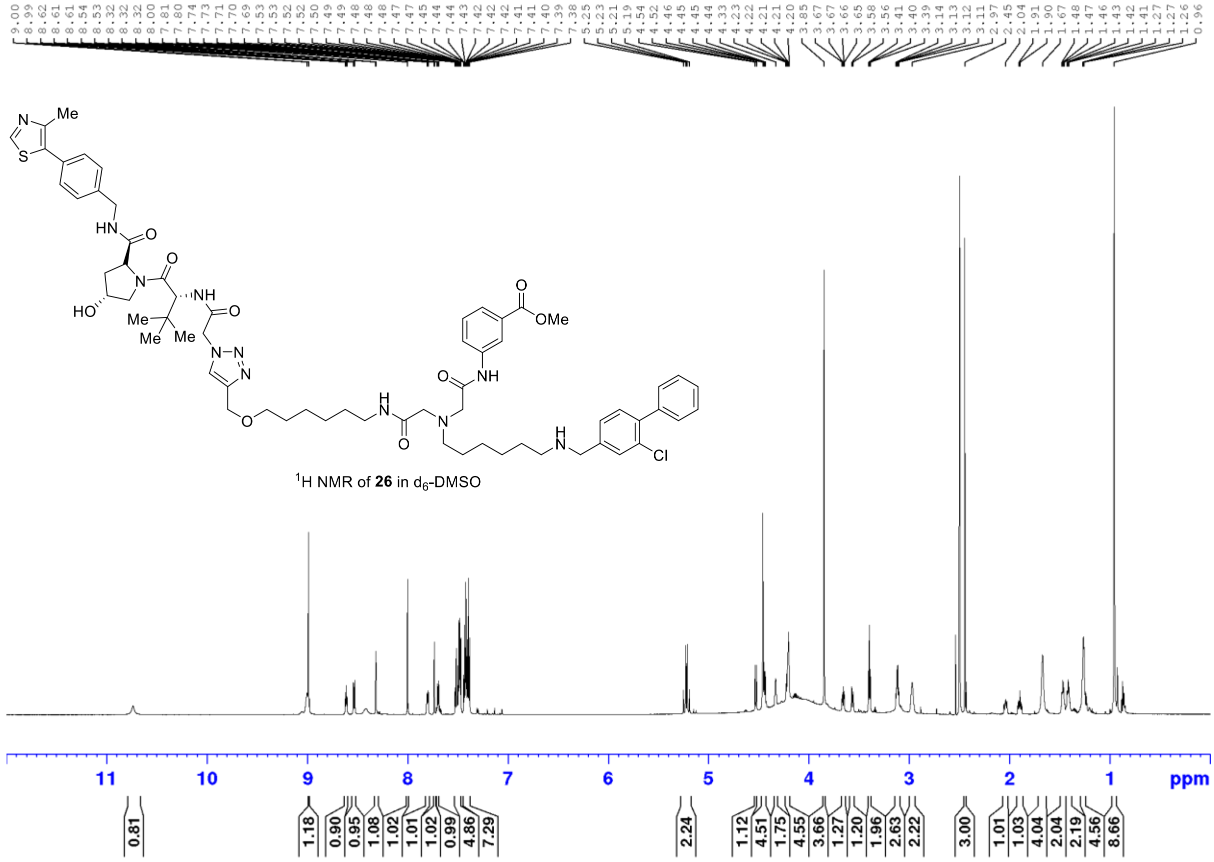

^{1}H NMR of **26** in d_6-DMSO

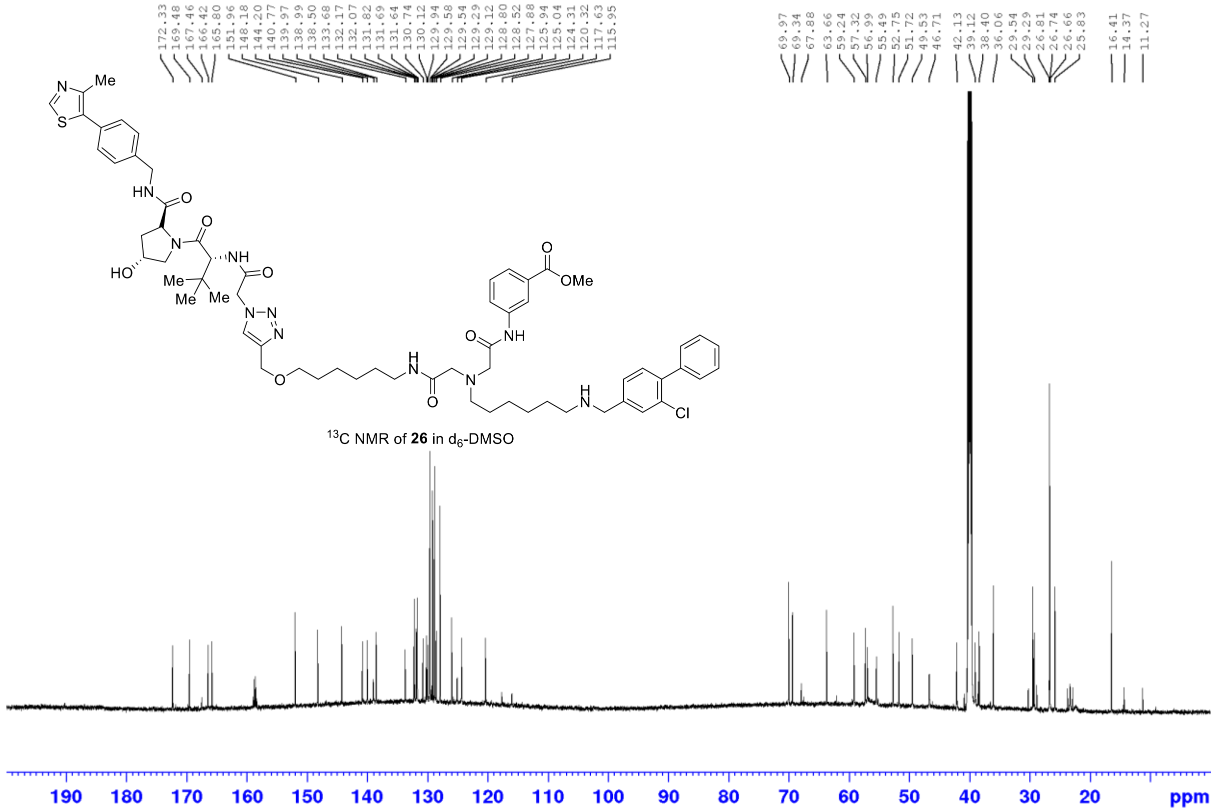

^{13}C NMR of **26** in d_6-DMSO

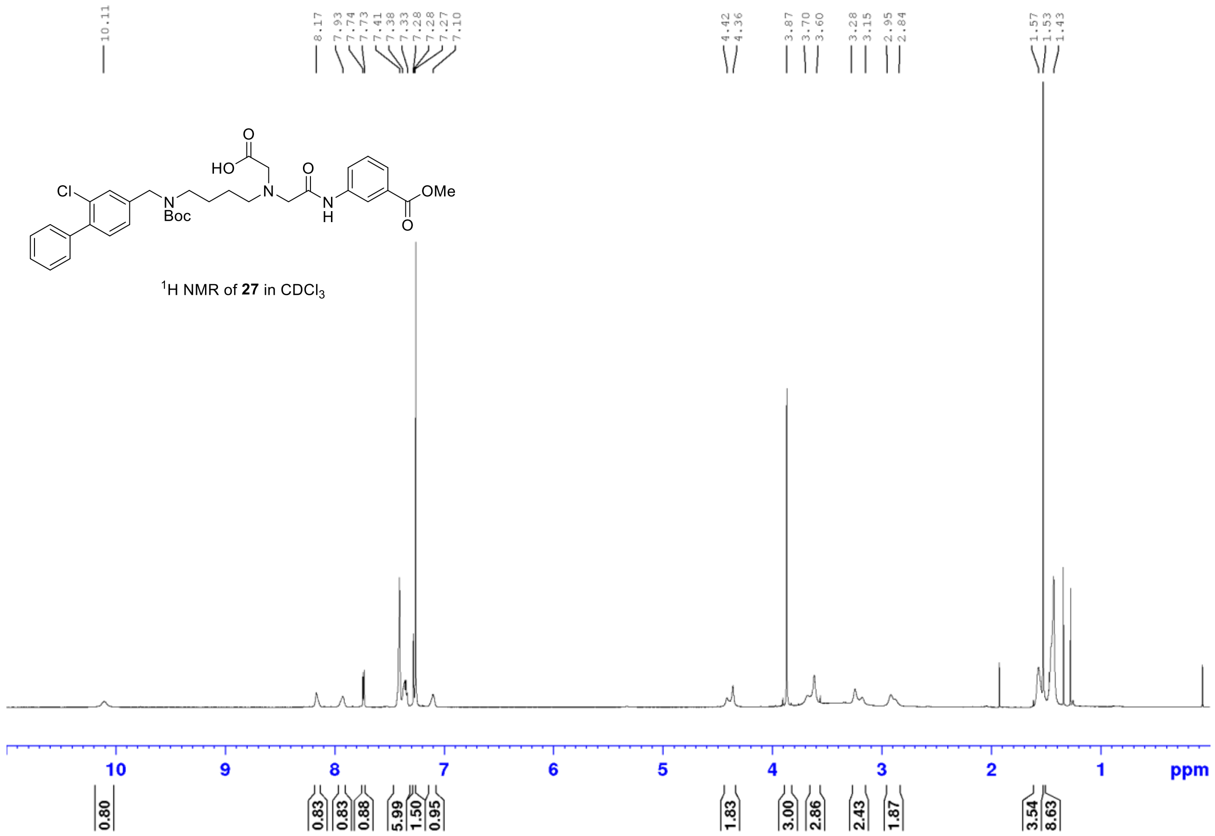

^{1}H NMR of **27** in $CDCl_3$

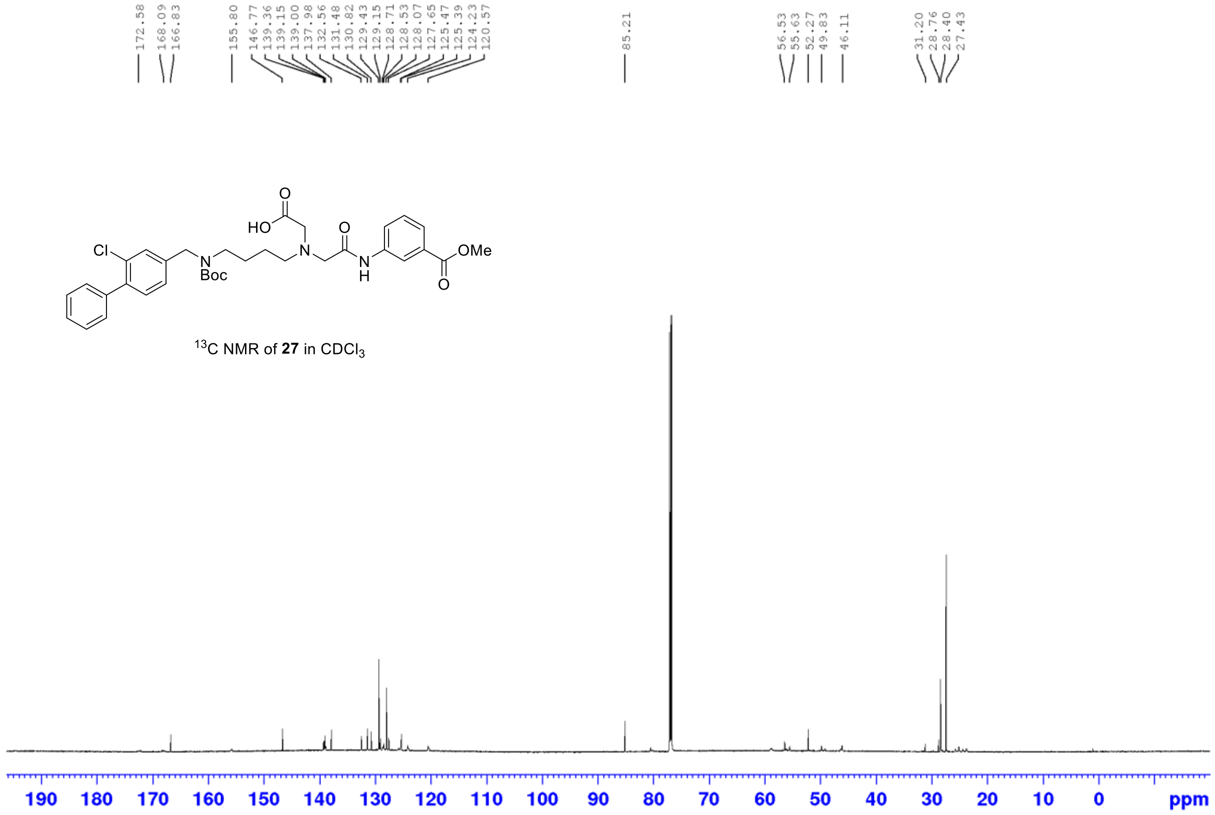

^{13}C NMR of **27** in $CDCl_3$

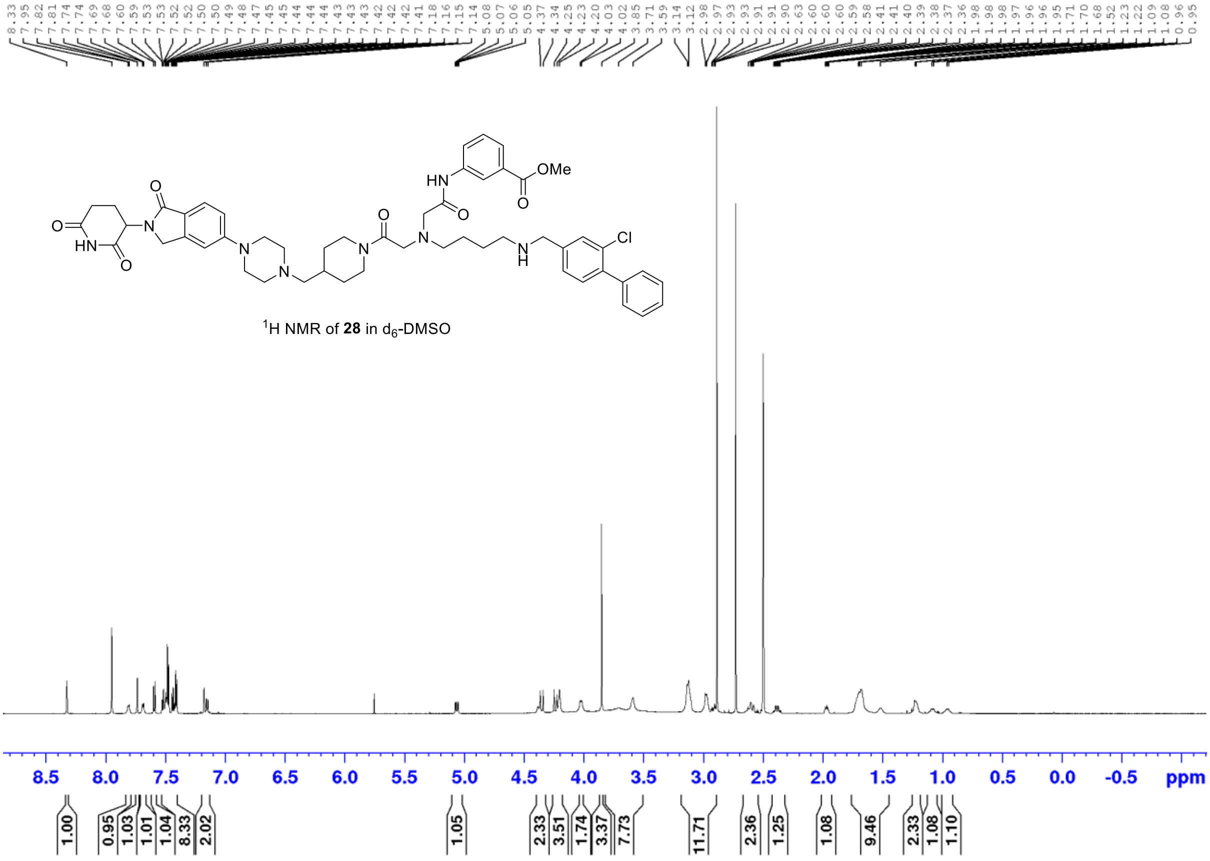

^{1}H NMR of **28** in d_6-DMSO

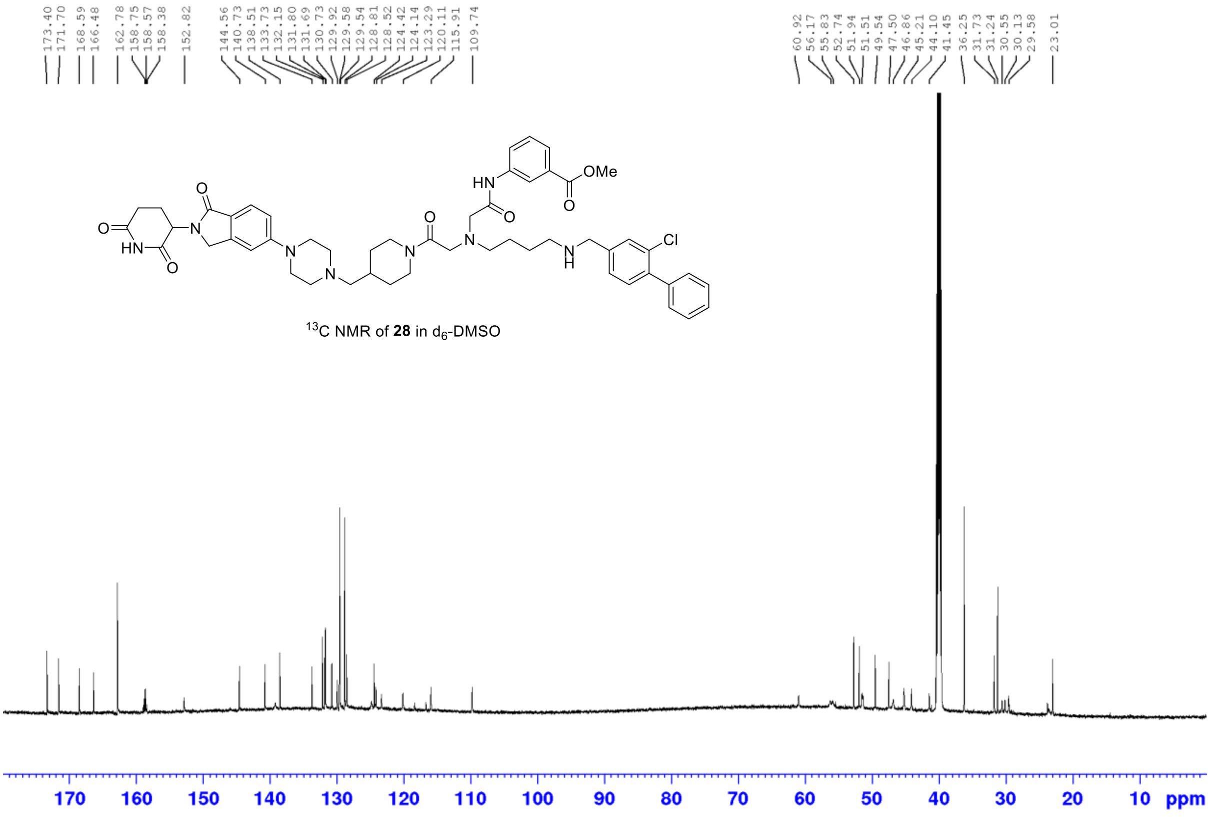

^{13}C NMR of **28** in d_6-DMSO

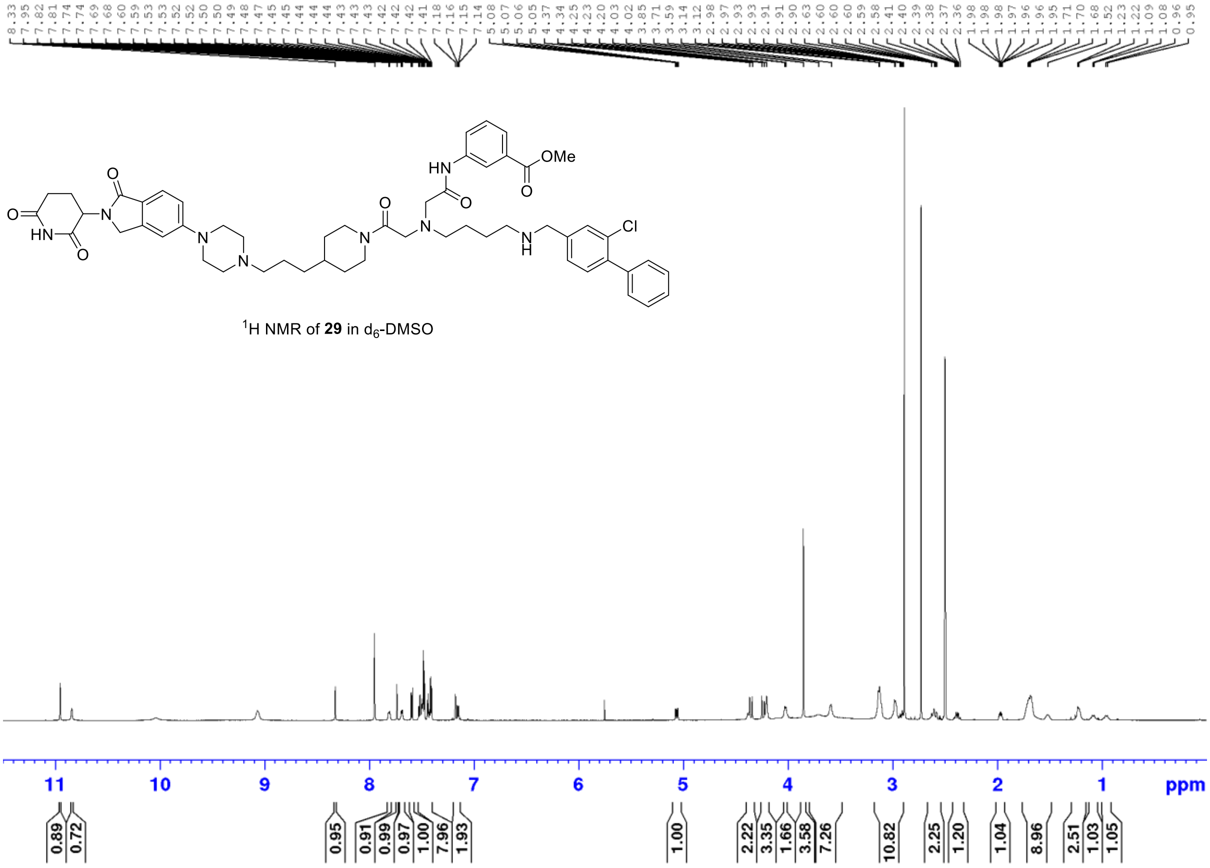

^{1}H NMR of **29** in d_6-DMSO

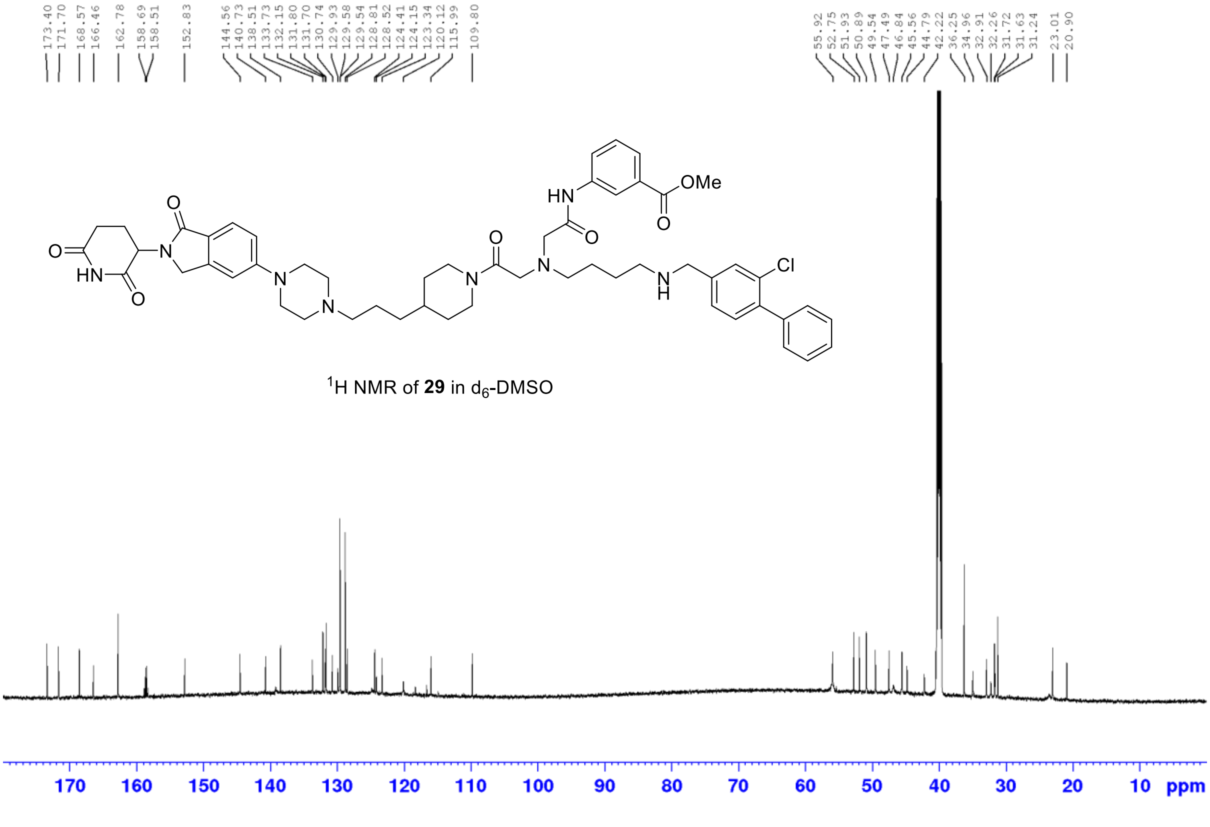

^{1}H NMR of **29** in d_6-DMSO
